# Comparative effectiveness of pharmacological interventions to prevent postoperative delirium: a network meta-analysis

**DOI:** 10.1038/s41598-021-91314-z

**Published:** 2021-06-07

**Authors:** Sun-Kyung Park, Taeyoon Lim, Hyeyeon Cho, Hyun-Kyu Yoon, Ho-Jin Lee, Ji-Hyun Lee, Seokha Yoo, Jin-Tae Kim, Won Ho Kim

**Affiliations:** Department of Anesthesiology and Pain Medicine, Seoul National University Hospital, Seoul National University College of Medicine, 101 Daehak-ro, Jongno-gu, Seoul, 03080 Republic of Korea

**Keywords:** Preventive medicine, Disorders of consciousness

## Abstract

Many pharmacologic agents were investigated for the effect to prevent delirium. We aimed to comprehensively compare the effect of the pharmacological interventions to prevent postoperative delirium. A Bayesian network meta-analysis of randomized trials was performed using random effects model. PubMed, the Cochrane Central Register of Controlled Trials, and Embase were searched on 20 January 2021. Randomized trials comparing the effect of a drug to prevent postoperative delirium with another drug or placebo in adult patients undergoing any kind of surgery were included. Primary outcome was the postoperative incidence of delirium. Eighty-six trials with 26,992 participants were included. Dexmedetomidine, haloperidol, and atypical antipsychotics significantly decreased the incidence of delirium than placebo [dexmedetomidine: odds ratio 0.51, 95% credible interval (CrI) 0.40–0.66, moderate quality of evidence (QOE); haloperidol: odds ratio 0.59, 95% CrI 0.37–0.95, moderate QOE; atypical antipsychotics: odds ratio 0.27, 95% CrI 0.14–0.51, moderate QOE]. Dexmedetomidine and atypical antipsychotics had the highest-ranking probabilities to be the best. However, significant heterogeneity regarding diagnostic time window as well as small study effects precludes firm conclusion.

## Introduction

Delirium is a frequent postoperative complication that is associated with prolonged hospital stay and poor patient outcomes^1^. Many pharmacologic agents were evaluated to prevent delirium after cardiac or non-cardiac surgery in previous studies. The agents included dexmedetomidine^2–4^, clonidine, other sedatives such as midazolam, ketamine and propofol, typical antipsychotics including haloperidol^5–7^, atypical antipsychotics including olanzapine and risperidone^8^, gabapentin^9^, pregabalin, steroid such as dexamethasone, and acetylcholinesterase inhibitor such as rivastigmine^10^. However, previous studies reported varying results from beneficial effect to potential harm. Ideal drugs to prevent postoperative delirium remains unestablished and comparative effectiveness of these agents are unclear.

Network meta-analysis is a useful statistical option that allows to simultaneously compare different interventions or drugs that have not been directly compared through adequately powered head-to-head randomized controlled trials^11–14^. Even without previous clinical trial, we can compare two interventions through a third common comparator^11,15^, with direct and indirect comparisons integrated to a single network comprising multiple interventions. In addition, it is possible to determine which arms are superior to the other arms according to statistical inference. Therefore, we could estimate the relative ranking of all interventions of the network.

The primary aim of this network meta-analysis was to assess the comparative efficacy of perioperative pharmacologic interventions to prevent delirium in the surgical setting. For that aim, we conducted a comprehensive network meta-analysis of randomized trials comparing any pharmacologic interventions to prevent postoperative delirium.

## Results

**Figure 1:**
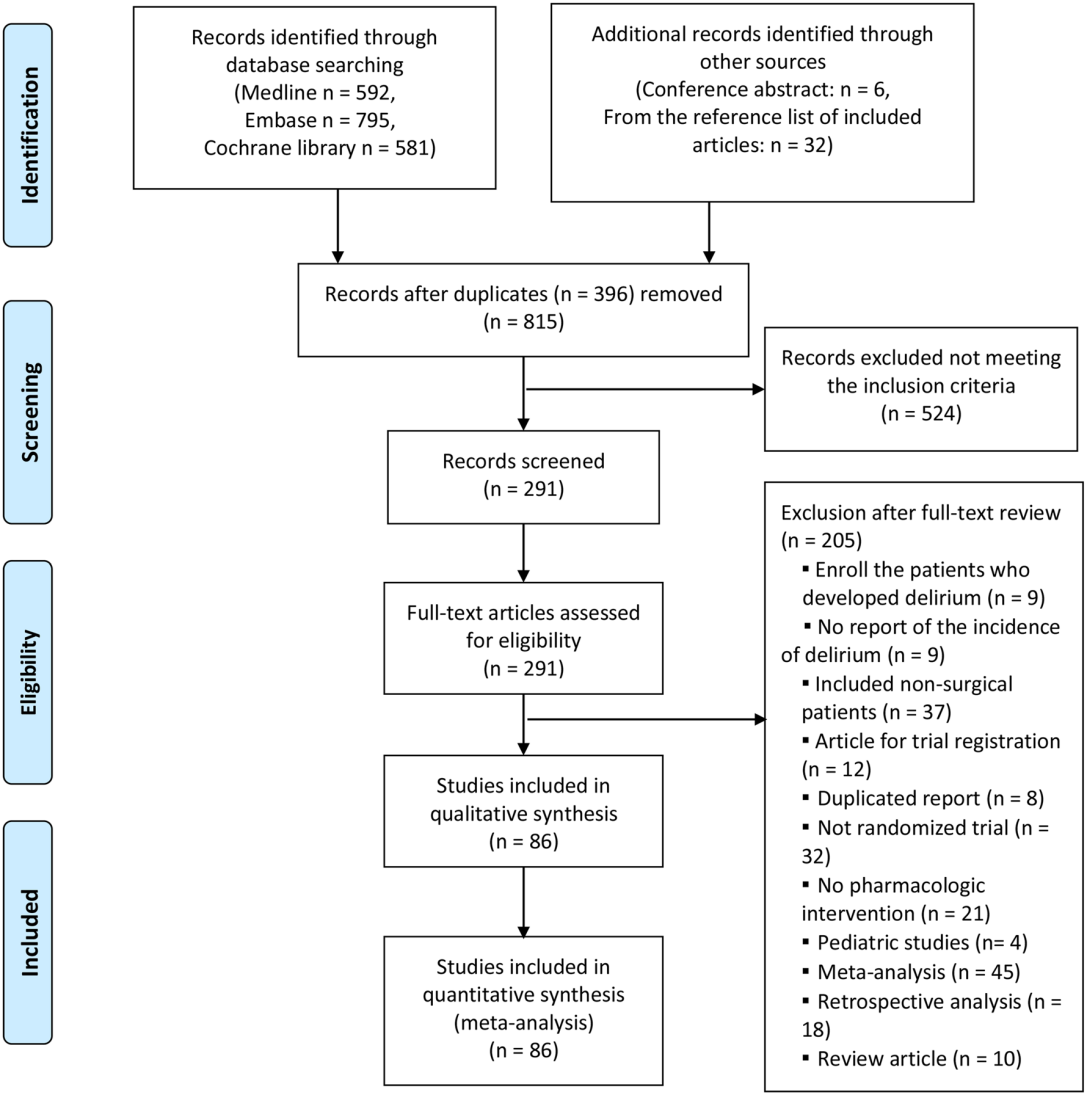
Flow diagram of our network meta-analysis.

Figure 1 shows the database search results and the number of exclusions from the current study. Initially, 1652 titles were screened, and an additional search was conducted from the clinical trial registration website, conference abstract, or reference list of included studies. Then we excluded 303 duplicate articles and 477 studies that do not meet our inclusion criteria. We carefully reviewed the full text of the remaining 247 articles. Then, we excluded 161 articles due to the reasons described in Fig. 1. Finally, 86 studies were included (Supplemental Text S1).

The characteristics of studies included in our analysis are shown in Supplemental Table S1. These 86 studies included 26,992 patients and numbers of patients and studies according to the different eighteen pharmacologic agent groups included in our network are shown in Table 1. We included twenty-one studies published before the year 2011 and 76 studies published between 2011 and 2020. Most studies reported the incidence of delirium for seven days after surgery. Three studies reported the incidence up to 6 weeks^5,16,17^, and 14 studies reported the incidence during hospital or ICU stay without reporting any specific period (Supplemental Table S1).

**Table 1:** Number of included studies and enrolled patients according to the individual interventions.

Interventions	Studies (n)	Intervention group (n)	Published year
Acetaminophen	1	66	2018
Acetylcholinesterase inhibitor	5	152	2005–2016
Benzodiazepine	8	421	2002–2018
Clonidine	2	66	2010
Dexmedetomidine	45	3959	2005–2020
Steroid	6	6476	2012–2018
Gabapentin, Pregabalin	4	526	2006–2018
Haloperidol	5	648	1999–2018
Ketamine	5	549	2009–2017
Lidocaine	2	55	2017–2019
Melatonin	4	285	2010–2014
Atypical antipsychotics (Olanzapine, Risperidone)	3	310	2007–2012
Propofol	13	665	2005–2019
Nimodipine	1	30	2017
Ondansetron	1	51	2014
Parecoxib	1	310	2017
Opioid	2	222	2009–2014
Volatile anesthetics	3	167	2011–2018
Placebo	66	12,034	1999–2019
Total	86	26,992	1999–2019

**Figure 2:**
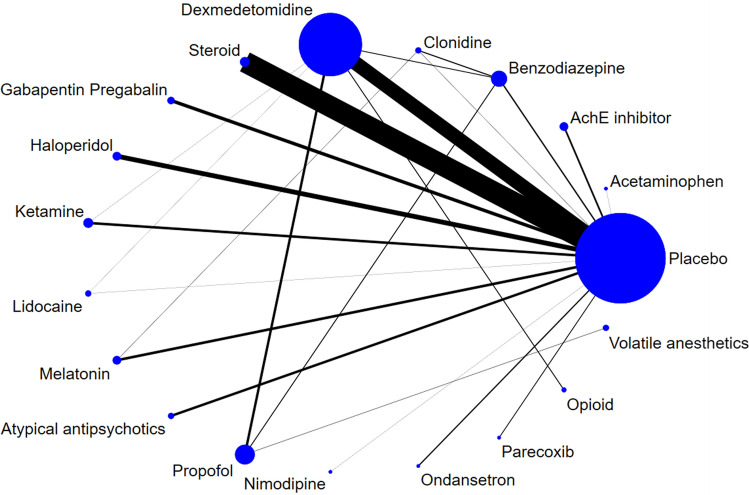
Network plot of our network of postoperative delirium. Nodes are weighted according to the number of patients with the respective interventions. Edges are weighted according to the number of patients included the comparison between the two connected modalities. No connection between any pair of the comparison means the comparison was made from the indirect comparison.

**Figure 3:**
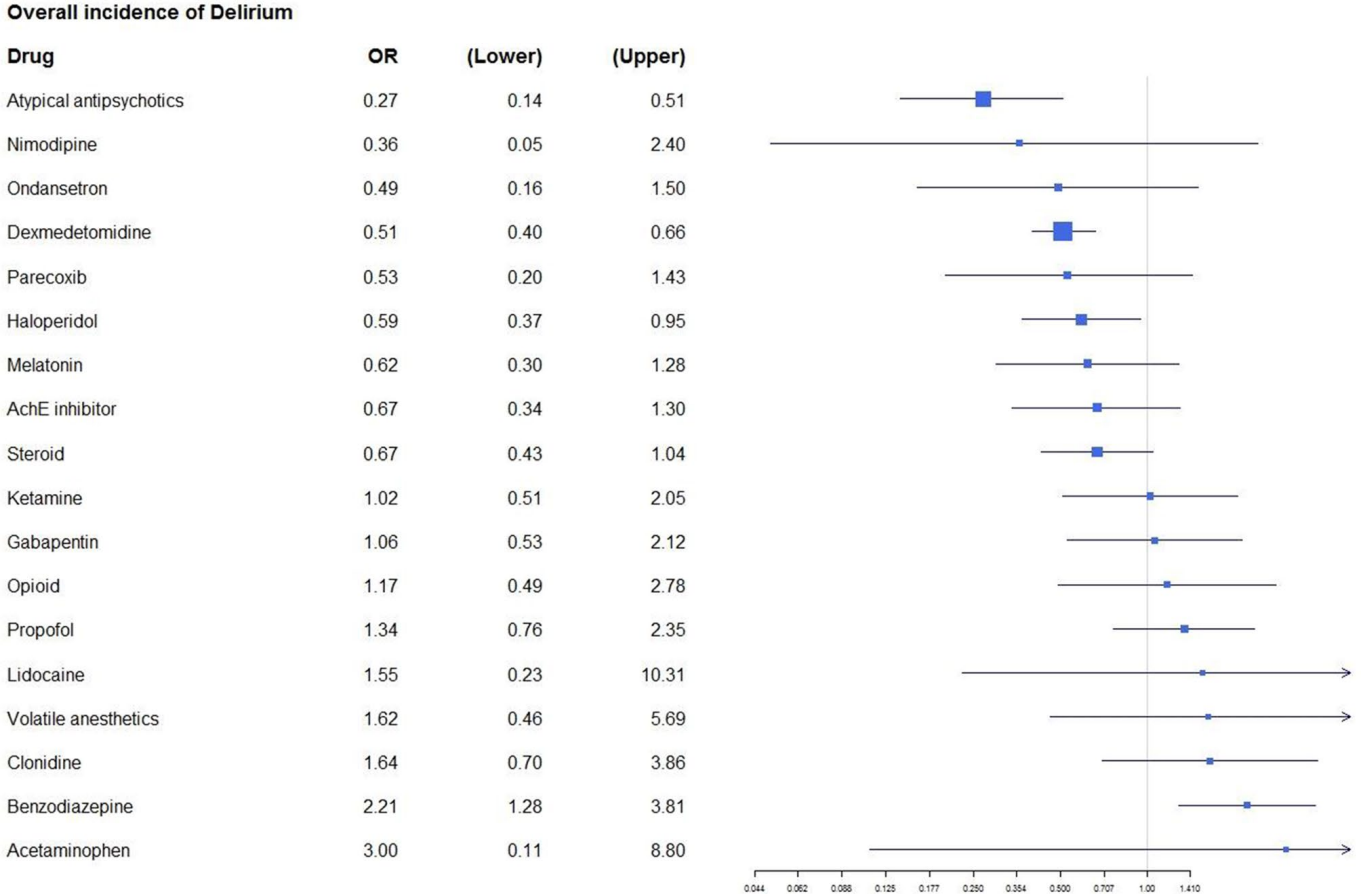
Predictive interval plots of the postoperative delirium network comparing each drug of interest with the placebo control. The blue square means the points estimates of odds ratio to prevent posteperative delirium. The solid black lines represent the credible intervals for summary odds ratio for each comparison. The vertical line corresponds to the line of no difference (odds ratio equals to 1).

Figure 2 shows the geometric view of our network for the incidence of postoperative delirium. Figure 3 shows the network effect size of all drugs compared to the control group for our primary outcome. Table 2 shows the network effect sizes of all possible pairs of drug comparisons. Compared to the placebo control, dexmedetomidine, haloperidol, and atypical antipsychotics significantly decreased the incidence of postoperative delirium [dexmedetomidine: OR 0.51, 95% CrI 0.40–0.66, SUCRA = 92.1, moderate quality of evidence (QOE); haloperidol: OR 0.59, 95% CrI 0.37–0.95, SUCRA = 67.4, moderate QOE; Atypical antipsychotics: OR 0.27, 95% CrI 0.14–0.51, SUCRA = 74.1, moderate QOE]. Among the comparisons between specific drugs, dexmedetomidine and haloperidol significantly decreased the incidence of delirium compared to benzodiazepine and clonidine. Atypical antipsychotics significantly decreased the incidence of delirium compared to benzodiazepine, clonidine, and ketamine.

**Table 2:** Network pooled estimates for the incidence of delirium in all pairs of comparisons.

Agents	Placebo	Acetaminophen	Acetylcholinesterase inhibitor	Benzodiazepine	Clonidine	Dexmedetomidine	Steroid	Gabapentin, Pregabalin	Haloperidol
Acetaminophen	3.00 (0.11–82.8)								
Acetylcholinesterase inhibitor	0.67 (0.34–1.30)	0.22 (0.01–6.58)							
Benzodiazepine	2.21 (1.28–3.81)	0.74 (0.03–21.25)	3.30 (1.40–7.79)						
Clonidine	1.64 (0.70–3.86)	0.55 (0.02–16.82)	2.45 (0.83–7.25)	0.74 (0.30–1.85)					
Dexmedetomidine	**0.51 (0.40**–0.66) *	0.17 (0.01–4.78)	0.77 (0.38–1.56)	**0.23 (0.14**–**0.40)**	**0.31 (0.13**–**0.75)**				
Steroid	0.67 (0.43–1.04)	0.22 (0.01–6.32)	1.00 (0.45–2.20)	**0.30 (0.15**–**0.60)**	0.41 (0.16–1.06)	1.30 (0.79–2.13)			
Gabapentin, Pregabalin	1.06 (0.53–2.12)	0.35 (0.01–10.48)	1.58 (0.60–4.15)	0.48 (0.20–1.16)	0.65 (0.21–1.95)	2.06 (0.99–4.30)	1.59 (0.70–3.60)		
Haloperidol	**0.59 (0.37**–**0.95)**	0.20 (0.01–5.60)	0.88 (0.39–1.99)	**0.27 (0.13**–**0.55)**	**0.36 (0.13**–**0.96)**	1.14 (0.67–1.95)	0.88 (0.46–1.68)	0.56 (0.24–1.29)	
Ketamine	1.02 (0.51–2.05)	0.34 (0.01–10.08)	1.52 (0.58–4.00)	0.46 (0.19–1.11)	0.62 (0.21–1.88)	1.98 (0.95–4.12)	1.53 (0.67–3.48)	0.96 (0.36–2.58)	1.73 (0.74–4.04)
Lidocaine	1.55 (0.23–10.31)	0.52 (0.01–23.55)	2.31 (0.31–17.27)	0.70 (0.10–5.02)	0.94 (0.12–7.56)	3.01 (0.45–20.25)	2.32 (0.33–16.25)	1.46 (0.19–11.01)	2.63 (0.37–18.60)
Melatonin	0.62 (0.30–1.28)	0.21 (0.01–6.17)	0.93 (0.35–2.47)	**0.28 (0.12**–**0.65)**	0.38 (0.14–1.06)	1.20 (0.57–2.56)	0.93 (0.41–2.12)	0.58 (0.21–1.59)	1.05 (0.44–2.50)
Atypical antipsychotics	**0.27 (0.14**–**0.51)**	0.09 (0.00–2.63)	0.40 (0.16–1.02)	**0.12 (0.05**–**0.28)**	**0.16 (0.06**–**0.48)**	0.52 (0.26–1.04)	0.40 (0.18–0.88)	0.25 (0.10–0.65)	0.46 (0.20–1.02)
Propofol	1.34 (0.76–2.35)	0.45 (0.02–12.89)	2.00 (0.83–4.77)	0.60 (0.30–1.20)	0.81 (0.30–2.22)	2.60 (1.55–4.34)	2.00 (0.98–4.07)	1.26 (0.52–3.08)	2.27 (1.09–4.74)
Nimodipine	0.36 (0.05–2.40)	0.12 (0.00–5.46)	0.53 (0.07–4.02)	0.16 (0.02–1.17)	0.22 (0.03–1.76)	0.69 (0.10–4.74)	0.54 (0.08–3.78)	0.34 (0.04–2.56)	0.61 (0.09–4.32)
Ondansetron	0.49 (0.16–1.50)	0.16 (0.00–5.41)	0.73 (0.20–2.69)	**0.22 (0.06**–**0.77)**	0.30 (0.07–1.22)	0.95 (0.30–3.00)	0.73 (0.22–2.44)	0.46 (0.12–1.73)	0.83 (0.25–2.81)
Parecoxib	0.53 (0.20–1.43)	0.18 (0.01–5.64)	0.79 (0.24–2.62)	**0.24 (0.08**–**0.75)**	0.32 (0.09–1.20)	1.03 (0.37–2.87)	0.79 (0.27–2.36)	0.50 (0.15–1.68)	0.90 (0.30–2.71)
Opioid	1.17 (0.49–2.78)	0.39 (0.01–12.05)	1.75 (0.59–5.21)	0.53 (0.20–1.42)	0.72 (0.21–2.39)	2.28 (1.00–5.22)	1.76 (0.67–4.62)	1.11 (0.37–3.35)	1.99 (0.74–5.34)
Volatile anesthetics	1.62 (0.46–5.69)	0.54 (0.02–18.77)	2.42 (0.59–10.01)	0.73 (0.20–2.73)	0.99 (0.22–4.44)	3.15 (0.92–10.81)	2.43 (0.65–9.09)	1.53 (0.36–6.41)	2.76 (0.72–10.53)

Agents	Ketamine	Lidocaine	Melatonin	Atypical antipsychotics	Propofol	Nimodipine	Ondansetron	Parecoxib	Opioid
Acetaminophen									
Acetylcholinesterase inhibitor									
Benzodiazepine									
Clonidine									
Dexmedetomidine									
Steroid									
Gabapentin, Pregabalin									
Haloperidol									
Ketamine									
Lidocaine	1.52 (0.20–11.46)								
Melatonin	0.61 (0.22–1.66)	0.40 (0.05–3.06)							
Atypical antipsychotics	**0.26 (0.10**–**0.68)**	0.17 (0.02–1.29)	0.43 (0.16–1.15)						
Propofol	1.31 (0.54–3.20)	0.86 (0.12–6.22)	2.16 (0.87–5.32)	4.98 (2.11–11.75)					
Nimodipine	0.35 (0.05–2.67)	0.23 (0.02–3.40)	0.58 (0.08–4.43)	1.33 (0.18–9.95)	0.27 (0.04–1.95)				
Ondansetron	0.48 (0.13–1.80)	0.32 (0.03–2.87)	0.79 (0.21–3.01)	1.82 (0.50–6.65)	0.37 (0.10–1.28)	1.37 (0.15–12.48)			
Parecoxib	0.52 (0.15–1.76)	0.34 (0.04–2.92)	0.86 (0.25–2.94)	1.98 (0.60–6.47)	0.40 (0.13–1.24)	1.48 (0.17–12.72)	1.08 (0.24–4.85)		
Opioid	1.15 (0.38–3.48)	0.76 (0.09–6.07)	1.89 (0.62–5.81)	4.37 (1.49–12.86)	0.88 (0.33–2.32)	3.28 (0.41–26.57)	2.40 (0.58–9.87)	2.21 (0.59–8.26)	
Volatile anesthetics	1.59 (0.38–6.67)	1.05 (0.11–10.16)	2.62 (0.63–10.93)	6.04 (1.47–24.86)	1.21 (0.40–3.72)	4.54 (0.46–44.44)	3.31 (0.62–17.85)	3.06 (0.62–15.18)	1.38 (0.31–6.11)

Data are shown as odds ratio (95% credible interval). Column intervention (numerator) was compared to row interventions (denominator) for the incidence of delirium after surgery. For example, *dexmedetomidine versus placebo for the incidence of delirium.

Global I^2^ was 56.6%. Loop-specific consistency was shown in the inconsistency plot (Supplemental Figure S1). The ROR from direct and indirect comparison shows significant inconsistency in our network effect sizes except the loop of control-dexmedetomidine-ketamine (ROR = 1.68, 95% CI 1.00–146.83, τ^2^ = 1.007, *P* = 0.467). All direct and indirect effect sizes for all pairs of comparison were shown in Supplemental Table S2.

The transitivity assumption of network meta-analysis was evaluated by reviewing the individual study baseline characteristics. The demographic information of each included study is shown in Supplemental Table S1. None of the regression coefficients of our meta-regression analysis was found to be statistically significant (Age: r =  − 0.007, 95% CI − 0.03 to 0.02, *P* = 0.556; Proportion of male: r =  − 0.003, 95% CI − 0.02 to 0.01, *P* = 0.628) (Supplemental Figure S2).

**Figure 4:**
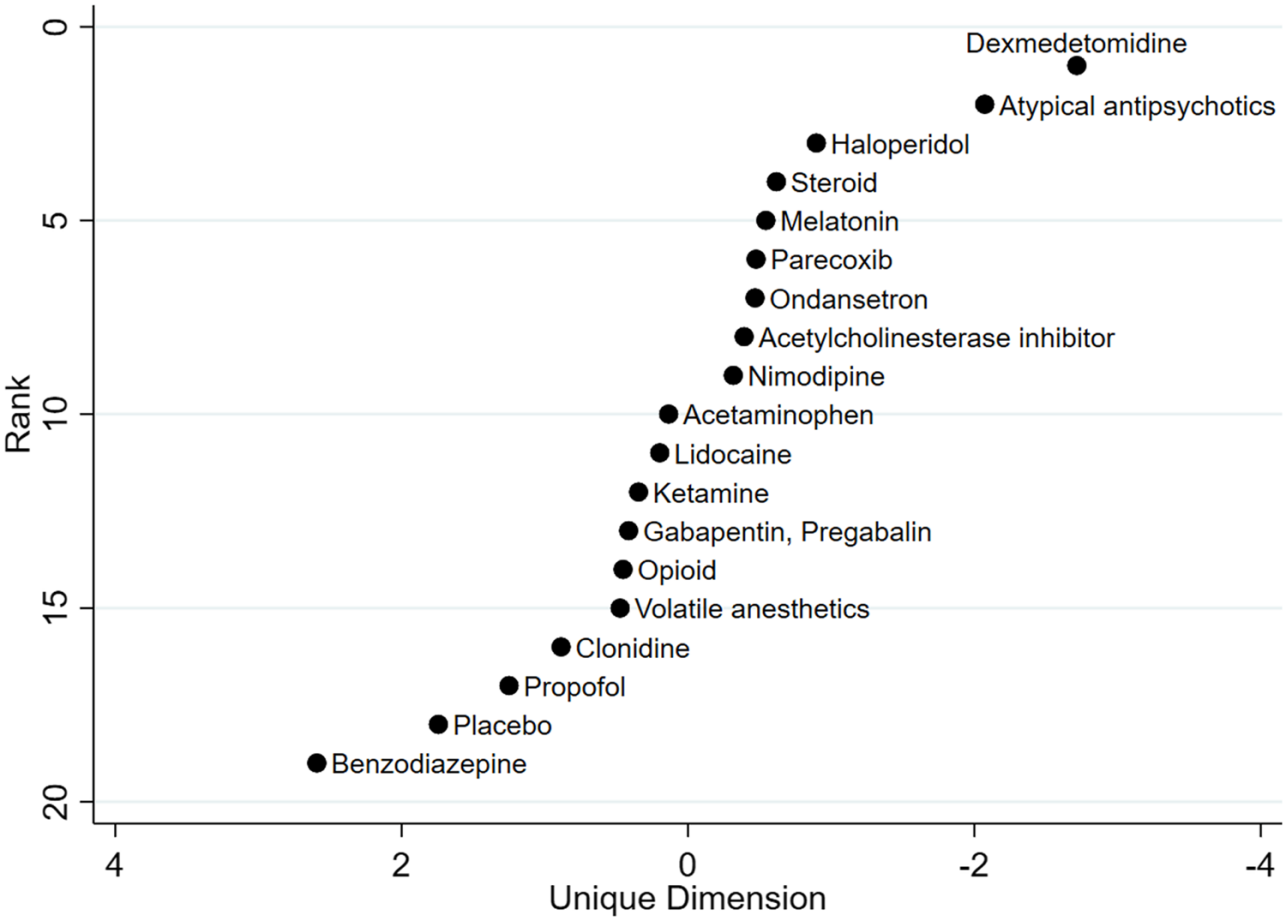
Relative ranking plots of drugs based on multidimensional scaling approach. The upper located interventions are ranked higher than the intervention located lower.

The comparative effectiveness of eighteen drugs of our network regarding our primary outcome was ranked. Supplemental Figures S3 shows the cumulative ranking plot of the individual drugs, which showed atypical antipsychotics showed highest cumulative probability to be ranked higher. Supplemental Figure S4 shows the rankogram which shows the same results. However, dexmedetomidine had the highest SUCRA value and the highest probability to be the best in all study populations (Supplemental Table S3). The relative ranking plot to reduce the incidence of the postoperative delirium was depicted based on the SUCRA values (Fig. 4). Dexmedetomidine, atypical antipsychotics, and haloperidol were ranked highly.

Supplemental Figure S5 shows the comparison-adjusted funnel plots that show the assessment of small-study effects according to each pair of comparison of our network. Our funnel plot showed asymmetry suggesting the small study effects and outliers for nimodipine, haloperidol and gabapentin.

The trials investigating the effect of acetaminophen, acetylcholine esterase inhibitor, benzodiazepine, dexmedetomidine, steroid, gabapentin, melatonin, propofol, nimodipine, and volatile anesthetics included studies at unclear or high risk of bias (Supplemental Figure S6). Supplemental Table S2 summarizes the GRADE QOE of the network estimates of all pharmacologic agents compared with placebo. Overall, the QOE from available studies ranged from high to low. Regarding network evidence, arms of haloperidol, atypical antipsychotics, and parecoxib showed high quality of evidence, while arms of acetaminophen, benzodiazepine, clonidine, propofol, nimodipine, and volatile anesthetics showed low quality of evidence. There was a serious imprecision in our network estimates because the 95% credible intervals crossed unity and were wide in ten among eighteen arms.

In the subgroup of patients receiving cardiac surgery (28 studies; Supplemental Figure S7), atypical antipsychotics had highest probability to be ranked higher (Supplemental Figure S8), which is consistent with the results of the full dataset. Atypical antipsychotics significantly reduced the incidence of delirium compared to the placebo group (OR 0.29, 95% CI 0.12–0.69). The relative ranking plot showed that atypical antipsychotics was ranked to be the best in patients receiving cardiac surgery (Supplemental Figure S9). Supplemental Figure S10 showed the network plot of the subgroup of the patients receiving non-cardiac surgery (56 studies). The cumulative ranking plots showed results similar to the full analysis (Supplemental Figure S11). The relative ranking plot showed that dexmedetomidine, atypical antipsychotics, and steroid were ranked highly (Supplemental Figure S12). Dexmedetomidine was ranked to be the best in the subgroup of non-cardiac surgery. In the subgroup analysis of the studies using CAM or CAM-ICU as diagnostic criteria for delirium (48 studies; Supplemental Figure S13), the cumulative ranking plots showed that atypical antipsychotics had the highest probability to be ranked higher (Supplemental Figure S14). Dexmedetomidine and atypical antipsychotics were ranked highly in the relative ranking plot in this subgroup (Supplemental Figure S15). Supplemental Figure S16 shows the network plot of the old-age subgroup (31 studies). Supplemental Figure S17 shows the cumulative ranking plots of the old-age subgroup. The relative ranking plot shows that steroid, atypical antipsychotics, and dexmedetomidine were ranked highly in the old-age subgroup (Supplemental Figure S18). The credibility of our network meta-regression analysis and subgroup analyses using ICEMAN tool was reported in Supplemental Text S2.

## Discussion

We conducted a comprehensive systematic review and network meta-analysis from 86 randomized trials enrolling 26,992 patients and compared eighteen pharmacologic agents for preventing postoperative delirium. The major findings of our study were as follows: (1) pooled estimates of dexmedetomidine, atypical antipsychotics and haloperidol showed significant benefits in decreasing the incidence of delirium. Relative ranking analysis showed that dexmedetomidine and atypical antipsychotics were ranked to be the best and the second than the other drugs. (2) postoperative delirium was reported in various criteria and were measured during various time window causing significant heterogeneity and (3) ten among eighteen arms of our network included studies at high or unclear risk of bias and six among eighteen arms showed low QOE according to our GRADE approach. Although we investigated the heterogeneity of the included clinical trials by performing the exploratory meta-regression for the potential effect modifiers such as patient demographics and subgroup analyses for cardiac and non-cardiac surgery, diagnostic criteria of CAM, and old-age group, heterogeneity issue still remains regarding the type of non-cardiac surgery and measurement time window. The readers should interpret our results carefully under these limitations.

Randomized trials included in our study and previous meta-analyses reported that dexmedetomidine showed a significant protective effect against postoperative delirium^2–4^. Several specific characteristics of dexmedetomidine could contribute to its effect to prevent postoperative delirium. Dexmedetomidine attenuates the inflammatory response, is lacking anticholinergic activity, and has an opioid-sparing effect^18^. Also, dexmedetomidine, unlike propofol, does not depress patients’ respiration and could shorten extubation times^18–20^. The most common adverse effects of dexmedetomidine are bradycardia and hypotension^19^. A previous meta-analysis reporting these side effects of dexmedetomidine showed that the incidence of hypotension was not significantly different, but the incidence of bradycardia was significantly higher compared to propofol sedation^20^, suggesting the necessity of close hemodynamic monitoring during dexmedetomidine use.

Several randomized trials reported that low-dose haloperidol could reduce the incidence of postoperative delirium^5–7^, while other studies reported no significant difference^21,22^. These discrepancies could be attributed the difference in the incidence of target population. Haloperidol blocks dopamine D2 receptor and releases acetylcholine. Preventing cholinergic deficiency is considered to be the mechanism of action by haloperidol^6^.

Relative ranking analysis revealed that atypical antipsychotics were ranked the first in our network analysis. Atypical antipsychotics block dopamine D2 receptor which inhibits the release of acetylcholine. As cholinergic deficiency was suggested to play a role in the pathophysiology of delirium^23^, enhanced release of acetylcholine resulting from the blockade of dopamine D2 receptor is associated is thought to be the mechanism of action of atypical antipsychotics.

To our knowledge, there was only one previous network meta-analysis comparing the effect of drugs to prevent postoperative delirium^24^. This network meta-analysis compared only the anesthetic agents including propofol, sevoflurane, desflurane, ketamine, midazolam, and dexmedetomidine. Therefore, their network is much smaller than us and proactive pharmacologic interventions to prevent delirium were not included. They concluded that dexmedetomidine could be the most effective sedative agent to reduce delirium and midazolam was associated with a higher incidence of delirium compared to other drugs.

The included clinical trials in a network meta-analysis should be sufficiently like each other regarding the patient baseline characteristics, surgical setting, and details of the intervention. This transitivity assumption is required to integrate the study outcomes quantitatively^25^. The data distribution of effect modifiers should be similar across the studies to ensure that a network meta-analysis is valid^26,27^. To address this transitivity issue, exploratory meta-regression analysis was performed for the available patient demographic factors. Although only age and sex were considered in this analysis due to availability, advanced age is considered to be an important predictor of postoperative delirium^28^. Differences in age across our target population could result in different study results. None of the regression coefficients of our meta-regression analyses was statistically significant.

Our study has several important limitations. Firstly, more than half of our network arms included randomized trials at unclear or higher risk of bias, resulting in low QOE. Secondly, significant heterogeneity regarding the type of surgery, target population, tool of delirium assessment and time window of postoperative period could impair transitivity assumption of network meta-analysis and result in less reliable network estimates. Significant heterogeneity in the criteria for diagnosing postoperative delirium was found, concerning not to meet the transitivity assumption of network meta-analysis. To address these heterogeneity issue, we performed subgroup analyses of the cardiac surgery, non-cardiac surgery, old-age patient, and the studies using CAM or CAM-ICU criteria. The results of our subgroup analyses showed similar results to the analysis of full dataset. Dexmedetomidine and atypical antipsychotics were ranked high consistently in these subgroup analyses. Thirdly, since seven drugs have only one to two clinical trials in their arms, wide credible intervals of network estimates caused serious imprecision and small study effects could affect our network analysis^29^. In addition, most of the randomized trials included in our network compared a drug with placebo or dexmedetomidine. As a result, many network estimates between the drugs came from indirect estimation and we could not assess the loop-specific consistency in all possible loop. Furthermore, significant inconsistency between direct and indirect evidence was observed in several loops. Finally, although most studies were published recently, studies included in our analyses were published over twenty years. As clinical practice changes and advances over this long period, our study endpoint could be affected.

In conclusion, our network meta-analysis of pharmacologic interventions to prevent postoperative delirium revealed that significant benefit of dexmedetomidine, atypical antipsychotics and haloperidol. Among eighteen drugs included in our network, relative ranking analysis showed that atypical antipsychotics was the best to prevent postoperative delirium. Our pooled network estimate of benzodiazepine could be more harmful than the placebo group. Dexmedetomidine has the highest SUCRA values and probability to be the best. Our subgroup analyses supported our results in the cardiac, non-cardiac surgery, old-age patients and studies using CAM or CAM-ICU criteria. However, significant heterogeneity regarding diagnostic time window as well as small study effects hinder firm conclusion. More than half of the drug arms have studies at high or unclear risk of bias. Our network meta-analysis provided the most comprehensive and up-to-date evidence regarding drugs preventing postoperative delirium. The discrepancy regarding the best drug between cumulative ranking and relative ranking warrant further randomized trial comparing dexmedetomidine and atypical antipsychotics.

## Methods

### Protocol and registration

Our review protocol was registered at PROPERO (CRD42018086852; principal investigator: Won Ho Kim; date of registration, January 29, 2018). This study was perfomed under the recommendations from the Cochrane Handbook for Systematic Reviews of Interventions^30,31^ and was reported according to the Preferred Reporting Items for Systemic Reviews and Meta-Analyses (PRISMA) extension statements for network meta-analysis (Supplemental Table S4)^32^.

### Eligibility criteria and study selection

We included randomized trials evaluating the effects of any of the following drugs used perioperatively to prevent delirium: dexmedetomidine, clonidine, midazolam, diazepam, morphine, pethidine, ketamine, propofol, haloperidol, olanzapine, risperidone, gabapentin, pregabalin, dexamethasone, donepezil, and rivastigmine. Any drug to prevent postoperative delirium was included in our network if there were at least one randomised trial comparing the drug with control or other specific drugs. The following drugs were added to our network after finishing the full searching to include all searched drugs investigated to prevent delirium: desflurane, sevoflurane, lidocaine, melatonin, nimodipine, ondansetron, and parecoxib. If two or more drugs can be integrated into a single drug group due to the similar category of mechanism of action, these drugs were integrated as a single group in our network because the network analysis of many drugs with only a few studies could yield the effect size of wide credible interval and therefore less reliable results. We grouped donepezil and rivastigmine as acetylcholine esterase inhibitor (AchE inhibitor), midazolam and diazepam as benzodiazepine, olanzapine and risperidone as atypical antipsychotics, gabapentin and pregabalin as a single group, desflurane and sevoflurane as volatile anesthetics, morphine and pethidine as opioid, and methylprednisolone and dexamethasone as steroid. As a result, eighteen drug groups were included in our network.

Eligible participants were adult patients who underwent any kind of surgery including cardiac and non-cardiac surgery. Cluster-randomized or quasi-randomized trials were not included. We excluded the trials that used the pharmacologic intervention as a treatment, not as a prevention of postoperative delirium. We also excluded the trials that involved patients with postoperative emergence delirium and delirium tremens.

### Information sources and search

Two investigators (SKP, WHK) independently searched Medline via Embase databases, PubMed interface, and the Cochrane Central Register of Controlled Trials (Central, Issue 12 of 2017) from its inception to December 2017. The search was updated on 20 January 2021. The two investigators independently reviewed the titles and abstracts of all searched studies to identify eligible trials. The search strategy for Embase, PubMed, and Cochrane central registry is reported in the Supplemental Text S3. An additional search was conducted by a bibliographic search of the studies included in our network meta-analysis or previous meta-analyses including any of the drugs of our delirium network.

### Data collection process and data items

Data were independently extracted from the included randomized trials by two investigators (SKP, WHK) using a uniform data extraction sheet developed by our authors. We resolved any discrepancies in data collection through a consensus discussion. The authors of our included trials were contacted by us for missing outcome data or unclear information or further details of the study results. The following items was collected from each study: the first author and location of the study; publication year; the number of enrolled participants; the definition of postoperative delirium; and the granular data of our study outcome.

There were many heterogeneous criteria used to assess postoperative delirium in our included trials. The pre-specified primary endpoint was postoperative delirium defined by the following currently available criteria including: Confusion Assessment Method (CAM), Confusion Assessment Method for the Intensive Care Unit (CAM-ICU), Intensive Care Delirium Screening Checklist (ICDSC), Neelon and Champagne confusion scale (NEECHAM), Nursing delirium screening score (Nu-DESC), diagnostic and statistical manual of mental disorder fourth edition (DSM-IV), delirium symptom interview (DSI), delirium observation screening (DOS), Richmond Agitation and Sedation Scale (RASS), and Delirium Rating Scale (DRS)^33^. The time window of outcome assessment was not considered, and the maximal incidence of any time point during postoperative period up to 6 weeks was selected as our primary outcome.

### Risk of bias within and across individual studies

We assessed the risk of bias within individual trials using the bias domains suggested in the Cochrane Handbook for Systemic Reviews of Interventions, version 5.1.0^30,34^. We also used the Grades of Recommendation, Assessment, Development and Evaluation (GRADE) approach to evaluate the quality of our evidence of network meta-analysis^35–37^. In this GRADE approach, we start the rating of direct evidence from randomized trials at a high quality and can rate down considering the risk of bias within each trial, publication bias, inconsistency, imprecision, and indirectness to the levels of moderate, low and very low quality. Secondly, to rate the indirect estimates, we started at the lowest rating of the two pairwise estimates which contributed to that indirect estimate as the first-order loops. We could rate down further for intransitivity or imprecision. Thirdly, if indirect and direct estimates of the GRADE approach were similar, we could assign the higher rating to the network meta-analysis estimates. To assess the presence of small-study effects in the network meta-analysis, we depicted a ‘comparison-adjusted’ funnel plot to examine any asymmetry for each pair of comparison^38^.

### Statistical analysis

Stata/SE version 14.0 (StataCorp, College Station, Texas, USA) was used to perform network meta-analysis using STATA command ‘networkplot’, ‘ifplot’, ‘netfunnel’, ‘network setup’, ‘network meta’, ‘netleague’, ‘network rank’, and ‘mdsrank’. Model fit was tested using “gemtc” package of R version 3.4.1. (R Foundation for Statistical Computing). Review Manager 5.3 (RevMan, The Cochrane Collaboration, Oxford, United Kingdom) was used to depict risk of bias assessment of individual studies. We used ‘mvmeta’ module of STATA to simultaneously compare the effects of different drugs based on the contrast-based model of Salanti et al.^39,40^ Direct comparisons between two pharmacological agents within any clinical trial as well as indirect comparisons of different drugs by a common comparator were integrated using the mixed technique comparison framework. We evaluated the network meta-analysis model fit by calculating the posterior residual deviance. We assessed the goodness of model fit by comparing Dbar, leverage, and deviance information criterion (DIC) between random effects and fixed effect models^41^. Random effects model with smaller DIC was preferred than the fixed effect model (Supplemental Table S5).

We presented our network by graphically depicting the pairwise associations between each drug arm. Network estimates of our primary endpoint were reported as odds ratios (OR) with 95% credible intervals (CrIs).

### Assessment of inconsistency

We evaluated the two important assumptions of network meta-analysis of consistency and transitivity that are related to the validity of network mixed estimates^26^. We assessed the plausibility of consistency assumption at three levels including network-specific, loop-specific, and at pairwise level. We calculated global I^2^ for network-specific level. Loop-specific consistency was evaluated within each closed triangle or quadratic loop. We calculated the inconsistency factor of the ratio of two odds ratios (ROR) from direct and indirect evidence in the loop in all triangle or quadratic loop. We estimated the 95% confidence interval of ROR as the absolute difference between indirect and direct effect size for each pair of comparison of the loop^42^. The ROR value of one means that direct and indirect effect size are in complete agreement and ROR values of two means that the difference between the direct and indirect network estimates is double. The heterogeneity of the indirect comparison was also investigated in term of τ^2^ that assesses between-study heterogeneity (A smaller value suggests less heterogeneity). For pairwise level, we compared difference between all direct and indirect effect estimates for all pairs of comparison.

We investigated the validity of transitivity assumption by reviewing the individual study characteristics. Network level exploratory meta-regression analysis were performed to evaluate the effect modification of patient demographic factors including patient sex and age.

### Additional analysis

To rank the drugs of our network regarding the preventive effects for our primary endpoint, the comparative effect size of all drugs to prevent delirium was estimated from a multidimensional scaling approach with a unique dimension^43^. We depicted relative ranking plots with this unique dimension. To show the comparative effectiveness of the drugs of our network, we also depicted cumulative ranking plots based on the analysis of the surface under the cumulative ranking (SUCRA) probabilities calculated by both the model with and without adjustment for small-study effects. The SUCRA value is defined by the percentage of effect obtained by a drug compared to an ideal imaginary drug that is always the best. For example, a SUCRA of 70 means that the corresponding drug is expected to have 70% of the effectiveness of the best imaginary drug.

We performed the following three subgroup analyses to address the heterogeneity of our included studies regarding the patient age, type of surgery and diagnostic criteria for delirium and to answer the question of which drug is more effective in the high-risk population with old age and undergoing cardiac surgery. The subgroup analyses in the cardiac and non-cardiac surgery were performed to test a priori hypothesis. The other subgroup analyses were added during the manuscript revision process. First, we performed a subgroup analyses of cardiac^10,18,44–69^ and non-cardiac surgery^5–9,16,17,21,22,70–116^. Second, we conducted a subgroup analysis for the studies which used CAM or CAM-ICU^6,9,10,17,18,21,22,48,49,51–53,55,57,59,61,62,65,66,69,70,74–80,83–85,88,89,92–102,106,107,115,117^. Third, we also performed a subgroup analysis for the studies which enrolled only old patients with age more than 70 years^5–10,17,18,22,55,57,59,66,72,73,77–80,85,88,93,94,97,98,101,102,104–106,116^. The credibility of our network meta-regression analysis and subgroup analyses were assessed using the Instrument to assess the Credibility of Effect Modification Analyses (ICEMAN)^118^.

## Supplementary information

Supplementary Informations.

## Data Availability

All other data is available in the Supplementary Information files. Any further information is available upon request from the corresponding author.

## References

[1] McCusker J, Cole M, Abrahamowicz M, Primeau F, Belzile E (2002). Delirium predicts 12-month mortality. Arch. Intern. Med..

[2] Sezai A (2011). Results of low-dose human atrial natriuretic peptide infusion in nondialysis patients with chronic kidney disease undergoing coronary artery bypass grafting: the NU-HIT (Nihon University working group study of low-dose HANP Infusion Therapy during cardiac surgery) trial for CKD. J. Am. Coll. Cardiol..

[3] Mori Y, Kamada T, Ochiai R (2014). Reduction in the incidence of acute kidney injury after aortic arch surgery with low-dose atrial natriuretic peptide: a randomised controlled trial. Eur. J. Anaesthesiol..

[4] Mitaka C, Kudo T, Haraguchi G, Tomita M (2011). Cardiovascular and renal effects of carperitide and nesiritide in cardiovascular surgery patients: a systematic review and meta-analysis. Crit. Care.

[5] Fukata S (2017). Haloperidol prophylaxis for preventing aggravation of postoperative delirium in elderly patients: a randomized, open-label prospective trial. Surg. Today.

[6] Wang W (2012). Haloperidol prophylaxis decreases delirium incidence in elderly patients after noncardiac surgery: a randomized controlled trial. Crit. Care Med..

[7] Kaneko T (1999). Prophylactic consecutive administration of haloperidol can reduce the occurrence of postoperative delirium in gastrointestinal surgery. Yonago Acta Med..

[8] Larsen KA (2010). Administration of olanzapine to prevent postoperative delirium in elderly joint-replacement patients: a randomized, controlled trial. Psychosomatics.

[9] Leung JM (2017). Perioperative gabapentin does not reduce postoperative delirium in older surgical patients: a randomized clinical trial. Anesthesiology.

[10] Gamberini M (2009). Rivastigmine for the prevention of postoperative delirium in elderly patients undergoing elective cardiac surgery—a randomized controlled trial. Crit. Care Med..

[11] Baker SG, Kramer BS (2002). The transitive fallacy for randomized trials: if A bests B and B bests C in separate trials, is A better than C?. BMC Med. Res. Methodol..

[12] Mihara T (2017). A network meta-analysis of the clinical properties of various types of supraglottic airway device in children. Anaesthesia.

[13] Norskov AK, Rosenstock CV, Leahy J, Walsh C (2017). Closing in on the best supraglottic airway for paediatric anaesthesia?. Anaesthesia.

[14] Choi SW, Lam DM (2016). Trials and tribulations of a meta-analyst. Anaesthesia.

[15] Song F, Altman DG, Glenny AM, Deeks JJ (2003). Validity of indirect comparison for estimating efficacy of competing interventions: empirical evidence from published meta-analyses. BMJ Clin. Res. Ed..

[16] Liptzin B, Laki A, Garb J, Fingeroth R, Krushell R (2005). Donepezil in the prevention and treatment of post-surgical delirium. Am. J. Geriatr. Psychiatry.

[17] Marcantonio ER, Palihnich K, Appleton P, Davis RB (2011). Pilot randomized trial of donepezil hydrochloride for delirium after hip fracture. J. Am. Geriatr. Soc..

[18] Djaiani G (2016). Dexmedetomidine versus propofol sedation reduces delirium after cardiac surgery: a randomized controlled trial. Anesthesiology.

[19] Lin YY, He B, Chen J, Wang ZN (2012). Can dexmedetomidine be a safe and efficacious sedative agent in post-cardiac surgery patients? A meta-analysis. Crit. Care.

[20] Liu X (2017). Dexmedetomidine vs propofol sedation reduces delirium in patients after cardiac surgery: a meta-analysis with trial sequential analysis of randomized controlled trials. J. Crit. Care.

[21] Khan BA (2018). Preventing postoperative delirium after major noncardiac thoracic surgery—a randomized clinical trial. J. Am. Geriatr. Soc..

[22] Kalisvaart KJ (2005). Haloperidol prophylaxis for elderly hip-surgery patients at risk for delirium: a randomized placebo-controlled study. J. Am. Geriatr. Soc..

[23] Hshieh TT, Fong TG, Marcantonio ER, Inouye SK (2008). Cholinergic deficiency hypothesis in delirium: a synthesis of current evidence. J. Gerontol. A Biol. Sci. Med. Sci..

[24] Cui Y, Li G, Cao R, Luan L, Kla KM (2020). The effect of perioperative anesthetics for prevention of postoperative delirium on general anesthesia: a network meta-analysis. J. Clin. Anesth..

[25] Choi SW, Lam DM (2017). Heterogeneity in meta-analyses. Comparing apples and oranges?. Anaesthesia.

[26] Salanti G (2012). Indirect and mixed-treatment comparison, network, or multiple-treatments meta-analysis: many names, many benefits, many concerns for the next generation evidence synthesis tool. Res. Synth. Methods.

[27] Jansen JP, Naci H (2013). Is network meta-analysis as valid as standard pairwise meta-analysis? It all depends on the distribution of effect modifiers. BMC Med..

[28] Aldecoa C (2017). European Society of Anaesthesiology evidence-based and consensus-based guideline on postoperative delirium. Eur. J. Anaesthesiol..

[29] Zhang Z, Xu X, Ni H (2013). Small studies may overestimate the effect sizes in critical care meta-analyses: a meta-epidemiological study. Crit. Care.

[30] Higgins, J. P. T. & (Eds), G. S. *Cochrane Handbook for Systemic Reviews of interventions (Version 5.1.0) [Updated March 2011]. The Cochrane Collaboration*, Available from www.cochrane-handbook.org. (2011).

[31] Smith AF, Carlisle J (2015). Reviews, systematic reviews and anaesthesia. Anaesthesia.

[32] Hutton B (2015). The PRISMA extension statement for reporting of systematic reviews incorporating network meta-analyses of health care interventions: checklist and explanations. Ann. Intern. Med..

[33] Neto AS (2012). Delirium screening in critically ill patients: a systematic review and meta-analysis. Crit. Care Med..

[34] Detweiler BN, Kollmorgen LE, Umberham BA, Hedin RJ, Vassar BM (2016). Risk of bias and methodological appraisal practices in systematic reviews published in anaesthetic journals: a meta-epidemiological study. Anaesthesia.

[35] Puhan MA (2014). A GRADE Working Group approach for rating the quality of treatment effect estimates from network meta-analysis. BMJ Clin. Res. Ed..

[36] Jelting Y (2017). Patient-controlled analgesia with remifentanil versus alternative parenteral methods for pain management in labour: a Cochrane systematic review. Anaesthesia.

[37] Karam O, Gebistorf F, Wetterslev J, Afshari A (2017). The effect of inhaled nitric oxide in acute respiratory distress syndrome in children and adults: a Cochrane systematic review with trial sequential analysis. Anaesthesia.

[38] Trinquart L, Chatellier G, Ravaud P (2012). Adjustment for reporting bias in network meta-analysis of antidepressant trials. BMC Med. Res. Methodol..

[39] Salanti G, Higgins JP, Ades AE, Ioannidis JP (2008). Evaluation of networks of randomized trials. Stat. Methods Med. Res..

[40] White IR, Barrett JK, Jackson D, Higgins JP (2012). Consistency and inconsistency in network meta-analysis: model estimation using multivariate meta-regression. Res. Synth. Methods.

[41] Dias S, Sutton AJ, Ades AE, Welton NJ (2013). Evidence synthesis for decision making 2: a generalized linear modeling framework for pairwise and network meta-analysis of randomized controlled trials. Med. Decis. Mak..

[42] Bucher HC, Guyatt GH, Griffith LE, Walter SD (1997). The results of direct and indirect treatment comparisons in meta-analysis of randomized controlled trials. J. Clin. Epidemiol..

[43] Chaimani A, Higgins JP, Mavridis D, Spyridonos P, Salanti G (2013). Graphical tools for network meta-analysis in STATA. PLoS ONE.

[44] Rubino AS (2010). Impact of clonidine administration on delirium and related respiratory weaning after surgical correction of acute type-A aortic dissection: results of a pilot study. Interact. Cardiovasc. Thorac. Surg..

[45] Balkanay OO, Goksedef D, Omeroglu SN, Ipek G (2015). The dose-related effects of dexmedetomidine on renal functions and serum neutrophil gelatinase-associated lipocalin values after coronary artery bypass grafting: a randomized, triple-blind, placebo-controlled study. Interact. Cardiovasc. Thorac. Surg..

[46] Corbett SM (2005). Dexmedetomidine does not improve patient satisfaction when compared with propofol during mechanical ventilation. Crit. Care Med..

[47] Kang F (2018). Effects of dexmedetomidine–isoflurane versus isoflurane anesthesia on brain injury after cardiac valve replacement surgery. J. Cardiothorac. Vasc. Anesth..

[48] Li X (2017). Impact of dexmedetomidine on the incidence of delirium in elderly patients after cardiac surgery: a randomized controlled trial. PLoS ONE.

[49] Liu X (2016). Dexmedetomidine versus propofol sedation improves sublingual microcirculation after cardiac surgery: a randomized controlled trial. J. Cardiothorac. Vasc. Anesth..

[50] Maldonado JR (2009). Dexmedetomidine and the reduction of postoperative delirium after cardiac surgery. Psychosomatics.

[51] Massoumi G, Mansouri M, Khamesipour S (2019). Comparison of the incidence and severity of delirium and biochemical factors after coronary artery bypass grafting with dexmedetomidine: a randomized double-blind placebo-controlled clinical trial study. ARYA Atheroscler..

[52] Park J (2014). Efficacy and safety of dexmedetomidine for postoperative delirium in adult cardiac surgery on cardiopulmonary bypass. Korean J. Thorac. Cardiovasc. Surg..

[53] Susheela AT (2017). The use of dexmedetomidine and intravenous acetaminophen for the prevention of postoperative delirium in cardiac surgery patients over 60 years of age: a pilot study. F1000Research.

[54] Priye S, Jagannath S, Singh D, Shivaprakash S, Reddy DP (2015). Dexmedetomidine as an adjunct in postoperative analgesia following cardiac surgery: a randomized, double-blind study. Saudi J. Anaesth..

[55] Shehabi Y (2009). Prevalence of delirium with dexmedetomidine compared with morphine based therapy after cardiac surgery: a randomized controlled trial (DEXmedetomidine compared to morphine-DEXCOM study). Anesthesiology.

[56] Sheikh TA, Dar BA, Akhter N, Ahmad N (2018). A comparative study evaluating effects of intravenous sedation by dexmedetomidine and propofol on patient hemodynamics and postoperative outcomes in cardiac surgery. Anesth. Essays Res..

[57] Shi C (2019). Effect of perioperative administration of dexmedetomidine on delirium after cardiac surgery in elderly patients: a double-blinded, multi-center, randomized study. Clin. Interv. Aging.

[58] Shu A, Liu X, Wang Q, Chen X, Zhan L (2017). Study on cerebral protective effect of dexmedetomidine during anesthesia in cardiac valve replacement surgery. Int. J. Clin. Exp. Med..

[59] Avidan MS (2017). Intraoperative ketamine for prevention of postoperative delirium or pain after major surgery in older adults: an international, multicentre, double-blind, randomised clinical trial. Lancet.

[60] Hudetz JA (2009). Ketamine attenuates delirium after cardiac surgery with cardiopulmonary bypass. J. Cardiothorac. Vasc. Anesth..

[61] Whitlock RP (2015). Methylprednisolone in patients undergoing cardiopulmonary bypass (SIRS): a randomised, double-blind, placebo-controlled trial. Lancet.

[62] Royse CF (2011). The influence of propofol or desflurane on postoperative cognitive dysfunction in patients undergoing coronary artery bypass surgery. Anaesthesia.

[63] Moscarelli M (2018). A trial of two anesthetic regimes for minimally invasive mitral valve repair. J. Cardiothorac. Vasc. Anesth..

[64] Hakim S, Othman A, Naoum D (2012). Early treatment with risperidone for subsyndromal delirium after on-pump cardiac surgery in the elderly: a randomized trial. Anesthesiology.

[65] Prakanrattana U, Prapaitrakool S (2007). Efficacy of risperidone for prevention of postoperative delirium in cardiac surgery. Anaesth. Intensive Care.

[66] Subramaniam B (2019). Effect of intravenous acetaminophen vERSUs placebo combined with propofol or dexmedetomidine on postoperative delirium among older patients following cardiac surgery: The DEXACET randomized clinical trial. JAMA.

[67] Dieleman JM (2012). Intraoperative high-dose dexamethasone for cardiac surgery: a randomized controlled trial. JAMA.

[68] Mardani D, Bigdelian H (2012). The effect of dexamethasone prophylaxis on postoperative delirium after cardiac surgery: a randomized trial. J. Res. Med. Sci..

[69] Sauer AM (2014). Intraoperative dexamethasone and delirium after cardiac surgery: a randomized clinical trial. Anesth. Analg..

[70] Lee H (2020). Effect of perioperative low-dose dexmedetomidine on postoperative delirium after living-donor liver transplantation: a randomized controlled trial. Transplant. Proc..

[71] Andjelković L, Novak-Jankovič V, Požar-Lukanovič N, Bosnić Z, Spindler-Vesel A (2018). Influence of dexmedetomidine and lidocaine on perioperative opioid consumption in laparoscopic intestine resection: a randomized controlled clinical trial. J. Int. Med. Res..

[72] Cheng XQ (2019). A multicentre randomised controlled trial of the effect of intra-operative dexmedetomidine on cognitive decline after surgery. Anaesthesia.

[73] Huyan T (2019). Perioperative dexmedetomidine reduces delirium in elderly patients after lung cancer surgery. Psychiatr. Danub..

[74] Kim JA (2019). Intraoperative use of dexmedetomidine for the prevention of emergence agitation and postoperative delirium in thoracic surgery: a randomized-controlled trial. Can. J. Anaesth..

[75] Sun Y (2019). Impact of postoperative dexmedetomidine infusion on incidence of delirium in elderly patients undergoing major elective noncardiac surgery: a randomized clinical trial. Drug Des. Dev. Ther..

[76] Greenberg S (2018). Postoperative intravenous acetaminophen for craniotomy patients: a randomized controlled trial. World Neurosurg..

[77] Chang YF (2018). Comparison of dexmedetomidine versus propofol on hemodynamics in surgical critically ill patients. J. Surg. Res..

[78] He F, Shen L, Zhong J (2018). A study of dexmedetomidine in the prevention of postoperative delirium in elderly patients after vertebral osteotomy. Int. J. Clin. Exp. Med..

[79] Lee C, Lee CH, Lee G, Lee M, Hwang J (2018). The effect of the timing and dose of dexmedetomidine on postoperative delirium in elderly patients after laparoscopic major non-cardiac surgery: a double blind randomized controlled study. J. Clin. Anesth..

[80] Mei B (2018). Intraoperative sedation with dexmedetomidine is superior to propofol for elderly patients undergoing hip arthroplasty: a prospective randomized controlled study. Clin. J. Pain.

[81] Mishina T (2018). Comparison between dexmedetomidine and midazolam as a sedation agent with local anesthesia in inguinal hernia repair: randomized controlled trial. Hernia.

[82] Tang CL (2018). Neuroprotective effect of bispectral index-guided fast-track anesthesia using sevoflurane combined with dexmedetomidine for intracranial aneurysm embolization. Neural Regen. Res..

[83] Xie S, Xie M (2018). Effect of dexmedetomidine on postoperative delirium in elderly patients undergoing hip fracture surgery. Pak. J. Pharm. Sci..

[84] Xuan Y (2018). Effects of dexmedetomidine for postoperative delirium after joint replacement in elderly patients: a randomized, double-blind, and placebo-controlled trial. Int. J. Clin. Exp. Med..

[85] Clemmesen CG, Lunn TH, Kristensen MT, Palm H, Foss NB (2018). Effect of a single pre-operative 125 mg dose of methylprednisolone on postoperative delirium in hip fracture patients; a randomised, double-blind, placebo-controlled trial. Anaesthesia.

[86] Farlinger C, Clarke H, Wong CL (2018). Perioperative pregabalin and delirium following total hip arthroplasty: a post hoc analysis of a double-blind randomized placebo-controlled trial. Can. J. Anaesth..

[87] Chang J (2017). Intraoperative and postoperative infusion of dexmedetomidine combined with butorphanol for intravenous patient-controlled analgesia after radical mastectomy: a double-blind, randomized clinical trial. Int. J. Clin. Exp. Med..

[88] Deiner S (2017). Intraoperative infusion of dexmedetomidine for prevention of postoperative delirium and cognitive dysfunction in elderly patients undergoing major elective noncardiac surgery: a randomized clinical trial. JAMA Surg..

[89] Yu D-N, Zhu Y, Ma J, Sun Q (2017). Comparison of post-anesthesia delirium in elderly patients treated with dexmedetomidine and midazolam maleate after thoracic surgery. Biomed. Res. (India).

[90] Dewinter G (2017). Systemic lidocaine fails to improve postoperative morphine consumption, postoperative recovery and quality of life in patients undergoing posterior spinal arthrodesis. A double-blind, randomized, placebo-controlled trial. Br. J. Anaesth..

[91] Li YN (2017). Effects of nimodipine on postoperative delirium in elderly under general anesthesia: a prospective, randomized, controlled clinical trial. Medicine.

[92] Chawla LS (2017). Acute kidney disease and renal recovery: consensus report of the Acute Disease Quality Initiative (ADQI) 16 Workgroup. Nat. Rev. Nephrol..

[93] Youn Y (2016). Rivastigmine patch reduces the incidence of postoperative delirium in older patients with cognitive impairment. Int. J. Geriatr. Psychiatry.

[94] Liu Y, Ma L, Gao M, Guo W, Ma Y (2016). Dexmedetomidine reduces postoperative delirium after joint replacement in elderly patients with mild cognitive impairment. Aging Clin. Exp. Res..

[95] Naik BI (2016). The effect of dexmedetomidine on postoperative opioid consumption and pain after major spine surgery. Anesth. Analg..

[96] Su X (2016). Dexmedetomidine for prevention of delirium in elderly patients after non-cardiac surgery: a randomised, double-blind, placebo-controlled trial. Lancet.

[97] Wu XH (2016). Low-dose dexmedetomidine improves sleep quality pattern in elderly patients after noncardiac surgery in the intensive care unit: a pilot randomized controlled trial. Anesthesiology.

[98] Guo Y, Sun LL, Chen ZF, Li QF, Jiang H (2015). Preventive effect of dexmedetomidine on postoperative delirium in elderly patients with oral cancer. Shanghai Kou Qiang Yi Xue.

[99] Wang K, Li C, Shi J, Wei H (2015). Effects of patient-controlled intravenous analgesia with dexmedetomidine and sufentanil on postoperative cognition in elderly patients after spine surgery. Zhonghua Yi Xue Za Zhi.

[100] Yang X, Li Z, Gao C, Liu R (2015). Effect of dexmedetomidine on preventing agitation and delirium after microvascular free flap surgery: a randomized, double-blind, control study. J. Oral Maxillofac. Surg..

[101] Šakić L, Tonković D, Godan BJ, Šakić K (2015). The influence of dexamethasone administration in spinal anesthesia for femur fracture on postoperative cognitive dysfunction. Period. Biol..

[102] Huang F (2014). Sedative effects of dexmedetomidine in post-operative elder patients on mechanical ventilation. Zhonghua Yi Xue Za Zhi.

[103] Dighe K, Clarke H, McCartney C, Wong C (2014). Perioperative gabapentin and delirium following total knee arthroplasty: a post-hoc analysis of a double-blind randomized placebo-controlled trial. Can. J. Anaesth..

[104] de Jonghe A (2014). Effect of melatonin on incidence of delirium among patients with hip fracture: a multicentre, double-blind randomized controlled trial. CMAJ.

[105] Yamaguchi Y, Mihara T, Taguri M, Yamaguchi O, Goto T (2014). Melatonin receptor agonist for the prevention of postoperative delirium in elderly patients: a randomized, double-blind, placebo-controlled trial. Intensive Care Med..

[106] Papadopoulos G (2014). The effect of ondansetron on postoperative delirium and cognitive function in aged orthopedic patients. Minerva Anestesiol..

[107] Ma PP, Piao MH, Wang YS, Ma HC, Feng CS (2013). Influence of dexmedetomidine and sub-anesthetic dose of ketamine on postoperative delirium in elderly orthopedic patients under total intravenous anesthesia. J. Jilin Univ. Med. Ed..

[108] Gupta K (2011). Dexmedetomidine premedication in relevance to ketamine anesthesia: a prospective study. Anesth. Essays Res..

[109] Wan L, Huang Q, Yue J, Lin L, Li S (2011). Comparison of sedative effect of dexmedetomidine and midazolam for post-operative patients undergoing mechanical ventilation in surgical intensive care unit. Chin. Crit. Care Med..

[110] Du J, Huang YG, Yu XR, Zhao N (2011). Effects of preoperative ketamine on the endocrine-metabolic and inflammatory response to laparoscopic surgery. Chin. Med. J..

[111] Nickkholgh A (2011). The use of high-dose melatonin in liver resection is safe: first clinical experience. J. Pineal Res..

[112] Gecaj-Gashi A, Hashimi M, Sada F, Salihu S, Terziqi H (2010). Prophylactic ketamine reduces incidence of postanaesthetic shivering. Niger. J. Med..

[113] Sultan SS (2010). Assessment of role of perioperative melatonin in prevention and treatment of postoperative delirium after hip arthroplasty under spinal anesthesia in the elderly. Saudi J. Anaesth..

[114] Sampson EL (2007). A randomized, double-blind, placebo-controlled trial of donepezil hydrochloride (Aricept) for reducing the incidence of postoperative delirium after elective total hip replacement. Int. J. Geriatr. Psychiatry.

[115] Leung JM (2006). Pilot clinical trial of gabapentin to decrease postoperative delirium in older patients. Neurology.

[116] Aizawa K (2002). A novel approach to the prevention of postoperative delirium in the elderly after gastrointestinal surgery. Surg. Today.

[117] Tanaka P (2017). The effect of desflurane versus propofol anesthesia on postoperative delirium in elderly obese patients undergoing total knee replacement: a randomized, controlled, double-blinded clinical trial. J. Clin. Anesth..

[118] Zhou C (2016). Levosimendan for prevention of acute kidney injury after cardiac surgery: a meta-analysis of randomized controlled trials. Am. J. Kidney Dis..

