# Supplementary material for: Comparative effectiveness of pharmacological interventions to prevent postoperative delirium: a network meta-analysis
Source: Sci Rep. 2021 Jun 7;11:11922. doi: 10.1038/s41598-021-91314-z (PMC8184858; doi:10.1038/s41598-021-91314-z)
Supplement: Supplementary file 1 — Supplementary Informations. [file 41598_2021_91314_MOESM1_ESM.docx]

## Comparative effectiveness of pharmacological interventions to prevent postoperative delirium: a network meta-analysis

: **Supplementary Information files**

**Sun-Kyung Park**, MD^1^, **Taeyoon Lim**, MD^1^, **Hyeyeon Cho**, MD^1^, **Hyun-Kyu Yoon**, MD^1^, **Ho-Jin Lee**, MD^1^, **Ji-Hyun Lee**, MD, PhD^1^, **Seokha Yoo**, MD^1^, **Jin-Tae Kim**, MD,PhD^1^, **Won Ho Kim**, MD,PhD^1^

^1^Department of Anesthesiology and Pain Medicine, Seoul National University Hospital, Seoul National University College of Medicine, Seoul, Republic of Korea

Correspondence to: **Won Ho Kim**, MD, PhD.

**List of contents**

| Title | page |
| --- | --- |
| **Supplemental Text S1.** List of included studies. | 4 |
| **Supplemental Text S2.** Credibility assessment of network meta-regression and subgroup analyses using the Instrument for assessing the Credibility of Effect Modification Analyses (ICEMAN) tool. | 22 |
| **Supplemental Text S3.** Search strategy and results. | 27 |
| **Supplemental Figure S1**. Loop-specific inconsistency plot. | 30 |
| **Supplemental Figure S2.** Exploratory meta-regression plot regarding patient age, the proportion of male gender. | 31 |
| **Supplemental Figure S3.** Cumulative ranking plots of the pharmacologic agents to prevent delirium. | 32 |
| **Supplemental Figure S4.** Rankogram of the pharmacologic agents to prevent delirium. | 33 |
| **Supplemental Figure S5.** Comparison-adjusted funnel plots for assessing small-study effects. | 34 |
| **Supplemental Figure S6**. Assessment of risk of bias in all included individual studies and their summary. | 35 |
| **Supplemental Figure S7**. Network plot of our network of postoperative delirium in the subgroup of patients receiving cardiac surgery. | 37 |
| **Supplemental Figure S8**. Cumulative ranking plots of the pharmacologic agents in the subgroup of patients receiving cardiac surgery. | 38 |
| **Supplemental Figure S9**. Relative ranking plot of the incidence of postoperative delirium in the subgroup of patients receiving cardiac surgery. | 39 |
| **Supplemental Figure S10**. Network plot of our network of postoperative delirium in the subgroup of patients receiving non-cardiac surgery. | 40 |
| **Supplemental Figure S11**. Cumulative ranking plots of the pharmacologic agents in the subgroup of patients receiving non-cardiac surgery. | 41 |
| **Supplemental Figure S12**. Relative ranking plot of the incidence of postoperative delirium in the subgroup of patients receiving non-cardiac surgery. | 42 |
| **Supplemental Figure S13**. Network plot of our network of postoperative delirium in the subgroup analysis for studies used CAM or CAM-ICU criteria. | 43 |
| **Supplemental Figure S14**. Cumulative ranking plots of the pharmacologic agents in the subgroup analysis for studies used CAM or CAM-ICU criteria. | 44 |
| **Supplemental Figure S15**. Relative ranking plot of the incidence of postoperative delirium in the subgroup analysis for studies used CAM or CAM-ICU criteria. | 45 |
| **Supplemental Figure S16**. Network plot of our network of postoperative delirium in the subgroup of patients aged ≥70 years. | 46 |
| **Supplemental Figure S17**. Cumulative ranking plots of the pharmacologic agents in the subgroup of patients aged ≥70 years. | 47 |
| **Supplemental Figure S18**. Relative ranking plot of the incidence of postoperative delirium in the subgroup of patients aged ≥70 years. | 48 |
| **Supplemental Table S1.** Characteristics of included studies. | 49 |
| **Supplemental Table S2**. Quality of evidence evaluated by GRADE approach for the incidence of delirium. | 59 |
| **Supplemental Table S3**. Adjusted SUCRA values and probability to the best and worst. | 62 |
| **Supplemental Table S4.** PRISMA network meta-analysis checklist. | 63 |
| **Supplemental Table S5.** Evaluation of the model fit. | 67 |

**Supplemental Text S1.** List of included studies.

**(1) Acetaminophen**

1. Greenberg S, Murphy GS, Avram MJ, Shear T, Benson J, Parikh KN, Patel A, Newmark R, Patel V, Bailes J, Szokol JW. Postoperative Intravenous Acetaminophen for Craniotomy Patients: A Randomized Controlled Trial. World Neurosurgery 2018;**109**:e554-e662

**(2) Acetylcholinesterase inhibitor (donepezil, rivastigmine)**

2. Liptzin B, Laki A, Garb J, Fingeroth R, Krushell R. Donepezil in the prevention and treatment of post-surgical delirium. American journal of geriatric psychiatry, 2005;**13**:1100-1106

3. Marcantonio ER, Palihnich K, Appleton P, Davis RB. Pilot randomized trial of donepezil hydrochloride for delirium after hip fracture. J Am Geriatr Soc 2011;**59** Suppl 2:S282-288

4. Sampson EL, Raven PR, Ndhlovu PN, Vallance A, Garlick N, Watts J, Blanchard MR, Bruce A, Blizard R, Ritchie CW. A randomized, double-blind, placebo-controlled trial of donepezil hydrochloride (Aricept) for reducing the incidence of postoperative delirium after elective total hip replacement. Int J Geriatr Psychiatry 2007;**22**:343-349

5. Gamberini M, Bolliger D, Buse GAL, Burkhart CS, Grapow M, Gagneux A, Filipovic M, Seeberger MD, Pargger H, Siegemund M, Carrel T, Seiler WO, Berres M, Strebel SP, Monsch AU, Steiner LA. Rivastigmine for the prevention of postoperative delirium in elderly patients undergoing elective cardiac surgery-A randomized controlled trial. Critical Care Medicine 2009;**37**:1762-1768

6. Youn Y, Shin H-W, Choi B-S, Kim S, Lee J-Y, Ha Y-C. Rivastigmine patch reduces the incidence of postoperative delirium in older patients with cognitive impairment International journal of geriatric psychiatry, 2016;**32**:1079-1084

**(3) Dexmedetomidine**

9. Andjelkovic L, Novak-Jankovic V, Pozar-Lukanovic N, Bosnic Z, Spindler-Vesel A. Influence of dexmedetomidine and lidocaine on perioperative opioid consumption in laparoscopic intestine resection: a randomized controlled clinical trial. Journal of international medical research 2018;**46**:5143‐5154

10. Azeem TMA, Yosif NE, Alansary AM, Esmat IM, Mohamed AK. Dexmedetomidine vs morphine and midazolam in the prevention and treatment of delirium after adult cardiac surgery; a randomized, double-blinded clinical trial. Saudi J Anaesth 2018;**12**:190-197

11. Balkanay OO, Goksedef D, Omeroglu SN, Ipek G. The dose-related effects of dexmedetomidine on renal functions and serum neutrophil gelatinase-associated lipocalin values after coronary artery bypass grafting: a randomized, triple-blind, placebo-controlled study. Interact Cardiovasc Thorac Surg 2015;**20**:209-214

12. Chang J, Wang X, Xin D, Sun Q, Jiang M, Ma J. Intraoperative and postoperative infusion of dexmedetomidine combined with butorphanol for intravenous patient-controlled analgesia after radical mastectomy: A double-blind, randomized clinical trial. International Journal of Clinical and Experimental Medicine 2017;**10**:2505-2513

13. Chang YF, Chao A, Shih PY, Hsu YC, Lee CT, Tien YW, Yeh YC, Chen LW. Comparison of dexmedetomidine versus propofol on hemodynamics in surgical critically ill patients. J Surg Res 2018;**228**:194-200

14. Cheng XQ, Mei B, Zuo YM, Wu H, Peng XH, Zhao Q, Liu XS, Gu E. A multicentre randomised controlled trial of the effect of intra-operative dexmedetomidine on cognitive decline after surgery. Anaesthesia 2019;**74**:741-750

15. Corbett SM, Rebuck JA, Greene CM, Callas PW, Neale BW, Healey MA, Leavitt BJ. Dexmedetomidine does not improve patient satisfaction when compared with propofol during mechanical ventilation. Critical Care Medicine 2005;**33**:940-945

16. Deiner S, Luo X, Lin HM, Sessler DI, Saager L, Sieber FE, Lee HB, Sano M, Jankowski C, Bergese SD, Candiotti K, Flaherty JH, Arora H, Shander A, Rock P. Intraoperative Infusion of Dexmedetomidine for Prevention of Postoperative Delirium and Cognitive Dysfunction in Elderly Patients Undergoing Major Elective Noncardiac Surgery: A Randomized Clinical Trial. JAMA Surg 2017;**152**:e171505

17. Djaiani G, Silverton N, Fedorko L, Carroll J, Styra R, Rao V, Katznelson R. Dexmedetomidine versus Propofol Sedation Reduces Delirium after Cardiac Surgery: A Randomized Controlled Trial. Anesthesiology 2016;**124**:362-368

18. Guo Y, Sun LL, Chen ZF, Li QF, Jiang H. [Preventive effect of dexmedetomidine on postoperative delirium in elderly patients with oral cancer]. Shanghai Kou Qiang Yi Xue 2015;**24**:236-239

19. Gupta K, Gupta A, Gupta PK, Rastogi B, Agarwal S, Lakhanpal M. Dexmedetomidine premedication in relevance to ketamine anesthesia: A prospective study. Anesth Essays Res 2011;**5**:87-91

20. He F, Shen L, Zhong J. A study of dexmedetomidine in the prevention of postoperative delirium in elderly patients after vertebral osteotomy. International Journal of Clinical and Experimental Medicine 2018;**11**:4984-4890

21. Huang F, Wang J, Yang X, Xu H, Kong J, Liu S, Jin J. Sedative effects of dexmedetomidine in post-operative elder patients on mechanical ventilation. Zhonghua yi xue za zhi 2014;**94**:3211-3215

22. Huyan T, Hu X, Peng H, Zhu Z, Li Q, Zhang W. Perioperative Dexmedetomidine Reduces Delirium in Elderly Patients after Lung Cancer Surgery. Psychiatr Danub 2019;**31**:95-101

23. Kang F, Tang C, Han M, Chai X, Huang X, Li J. Effects of Dexmedetomidine-Isoflurane versus Isoflurane Anesthesia on Brain Injury After Cardiac Valve Replacement Surgery. Journal of Cardiothoracic and Vascular Anesthesia 2018;**32**:1581-1586

24. Kim JA, Ahn HJ, Yang M, Lee SH, Jeong H, Seong BG. Intraoperative use of dexmedetomidine for the prevention of emergence agitation and postoperative delirium in thoracic surgery: a randomized-controlled trial. Can J Anaesth 2019;**66**:371-379

25. Lee C, Lee CH, Lee G, Lee M, Hwang J. The effect of the timing and dose of dexmedetomidine on postoperative delirium in elderly patients after laparoscopic major non-cardiac surgery: A double blind randomized controlled study. J Clin Anesth 2018;**47**:27-32

26. Li X, Yang J, Nie XL, Zhang Y, Li XY, Li LH, Wang DX, Ma D. Impact of dexmedetomidine on the incidence of delirium in elderly patients after cardiac surgery: A randomized controlled trial. PLoS One 2017;**12**:e0170757

27. Liu X, Zhang K, Wang W, Xie G, Cheng B, Wang Y, Hu Y, Fang X. Dexmedetomidine Versus Propofol Sedation Improves Sublingual Microcirculation After Cardiac Surgery: a Randomized Controlled Trial. J Cardiothorac Vasc Anesth, 2016;**30**:1509-1515

28. Liu Y, Ma L, Gao M, Guo W, Ma Y. Dexmedetomidine reduces postoperative delirium after joint replacement in elderly patients with mild cognitive impairment. Aging Clin Exp Res 2016;**28**:729-36

29. Ma PP, Piao MH, Wang YS, Ma HC, Feng CS. Influence of dexmedetomidine and sub-anesthetic dose of ketamine on postoperative delirium in elderly orthopedic patients under total intravenous anesthesia. Journal of Jilin University Medicine Edition 2013;**39**:128-132

30. Maldonado JR, Wysong A, Van Der Starre PJA, Block T, Miller C, Reitz BA. Dexmedetomidine and the reduction of postoperative delirium after cardiac surgery. Psychosomatics 2009;**50**:206-217

31. Massoumi G, Mansouri M, Khamesipour S. Comparison of the incidence and severity of delirium and biochemical factors after coronary artery bypass grafting with dexmedetomidine: A randomized double-blind placebo-controlled clinical trial study. ARYA Atheroscler 2019;**15**:14-21

32. Mei B, Meng G, Xu G, Cheng X, Chen S, Zhang Y, Zhang M, Liu X, Gu E. Intraoperative Sedation With Dexmedetomidine is Superior to Propofol for Elderly Patients Undergoing Hip Arthroplasty: A Prospective Randomized Controlled Study. Clin J Pain 2018;**34**:811-817

33. Mishina T, Aiba T, Hiramatsu K, Shibata Y, Yoshihara M, Aoba T, Yamaguchi N, Kato T. Comparison between dexmedetomidine and midazolam as a sedation agent with local anesthesia in inguinal hernia repair: randomized controlled trial. Hernia 2018;**22**:471-478

34. Naik BI, Nemergut EC, Kazemi A, Fernández L, Cederholm SK, McMurry TL, Durieux ME. The Effect of Dexmedetomidine on Postoperative Opioid Consumption and Pain after Major Spine Surgery. Anesthesia and Analgesia 2016;**122**:1646-1653

35. Susheela AT, Packiasabapathy S, Gasangwa DV, Patxot M, O'Neal J, Marcantonio E, Subramaniam B. The use of dexmedetomidine and intravenous acetaminophen for the prevention of postoperative delirium in cardiac surgery patients over 60 years of age: a pilot study. F1000Res 2017;**6**:1842

36. Priye S, Jagannath S, Singh D, Shivaprakash S, Reddy DP. Dexmedetomidine as an adjunct in postoperative analgesia following cardiac surgery: A randomized, double-blind study. Saudi J Anaesth 2015;**9**:353-358

37. Park J, Bang S, Chee H, Kim J, Lee S, Shin J. Efficacy and Safety of Dexmedetomidine for Postoperative Delirium in Adult Cardiac Surgery on Cardiopulmonary Bypass. Korean J Thorac Cardiovasc Surg 2014;**47**:249-254

38. Shehabi Y, Grant P, Wolfenden H, Hammond N, Bass F, Campbell M, Chen J. Prevalence of delirium with dexmedetomidine compared with morphine based therapy after cardiac surgery: A randomized controlled trial (DEXmedetomidine compared to morphine-DEXCOM study). Anesthesiology 2009;**111**:1075-1084

39. Sheikh TA, Dar BA, Akhter N, Ahmad N. A Comparative Study Evaluating Effects of Intravenous Sedation by Dexmedetomidine and Propofol on Patient Hemodynamics and Postoperative Outcomes in Cardiac Surgery. Anesth Essays Res 2018;**12**:555-560

40. Shi C, Jin J, Qiao L, Li T, Ma J, Ma Z. Effect of perioperative administration of dexmedetomidine on delirium after cardiac surgery in elderly patients: a double-blinded, multi-center, randomized study. Clin Interv Aging 2019;**14**:571-575

41. Shu A, Liu X, Wang Q, Chen X, Zhan L. Study on cerebral protective effect of dexmedetomidine during anesthesia in cardiac valve replacement surgery. Int J Clin Exp Med 2017;**10**:1066-1072

42. Su X, Meng Z, Wu X, Cui F, Li H, Wang D, Zhu X, Zhu S, Maze M, Ma D. Dexmedetomidine for prevention of delirium in elderly patients after non-cardiac surgery: a randomised, double-blind, placebo-controlled trial. Lancet (london, england), 2016;**388**:1893-1902

43. Sun Y, Jiang M, Ji Y, Sun Y, Liu Y, Shen W. Impact of postoperative dexmedetomidine infusion on incidence of delirium in elderly patients undergoing major elective noncardiac surgery: A randomized clinical trial. Drug Des Devel Ther 2019;**13**:2911-2922

44. Tang CL, Li J, Zhang ZT, Zhao B, Wang SD, Zhang HM, Shi S, Zhang Y, Xia ZY. Neuroprotective effect of bispectral index-guided fast-track anesthesia using sevoflurane combined with dexmedetomidine for intracranial aneurysm embolization. Neural Regen Res 2018;**13**:280-288

45. Wan L, Huang Q, Yue J, Lin L, Li S. Comparison of sedative effect of dexmedetomidine and midazolam for post-operative patients undergoing mechanical ventilation in surgical intensive care unit. Chinese Crit Care Med 2011;**23**:543-546

46. Wang K, Li C, Shi J, Wei H. Effects of patient-controlled intravenous analgesia with dexmedetomidine and sufentanil on postoperative cognition in elderly patients after spine surgery. Zhonghua Yi Xue Za Zhi 2015;**95**:2437-2441

47. Wu XH, Cui F, Zhang C, Meng ZT, Wang DX, Ma J, Wang GF, Zhu SN, Ma D. Low-dose Dexmedetomidine Improves Sleep Quality Pattern in Elderly Patients after Noncardiac Surgery in the Intensive Care Unit: A Pilot Randomized Controlled Trial. Anesthesiology 2016;**125**:979-991

48. Xie S, Xie M. Effect of dexmedetomidine on postoperative delirium in elderly patients undergoing hip fracture surgery. Pak J Pharm Sci 2018;**31**:2277-2281

49. Xuan Y, Fan R, Chen J, Wang Y, Wu J, Yang J, Luo Y. Effects of dexmedetomidine for postoperative delirium after joint replacement in elderly patients: a randomized, double-blind, and placebo-controlled trial. Int J Clin Exp Med 2018;**11**:13147‐57

50. Yang X, Li Z, Gao C, Liu R. Effect of dexmedetomidine on preventing agitation and delirium after microvascular free flap surgery: a randomized, double-blind, control study. J Oral Maxillofac Surg 2015:**73**:1065-1072

51. Lee H, Yang SM, Chung J, Oh HW, Yi NJ, Suh KS, Oh SY, Ryu HG. Effect of Perioperative Low-Dose Dexmedetomidine on Postoperative Delirium After Living-Donor Liver Transplantation: A Randomized Controlled Trial. Transplant Proc 2020;**52**:239-245

52. Yu D-N, Yi Zhu, Jue Ma, Sun Q. Comparison of post-anesthesia delirium in elderly patients treated with dexmedetomidine and midazolam maleate after thoracic surgery. Biomedical research (india) 2017;**28**:6852‐6855

80. Subramaniam B, Shankar P, Shaefi S, Mueller A, O'Gara B, Banner-Goodspeed V, Gallagher J, Gasangwa D, Patxot M, Packiasabapathy S, Mathur P, Eikermann M, Talmor D, Marcantonio ER. Effect of Intravenous Acetaminophen vs Placebo Combined With Propofol or Dexmedetomidine on Postoperative Delirium Among Older Patients Following Cardiac Surgery: The DEXACET Randomized Clinical Trial. JAMA 2019;**321**:686-696

**(4) Benzodiazepine**

7. Aizawa K, Kanai T, Saikawa Y, Takabayashi T, Kawano Y, Miyazawa N, Yamamoto T. A novel approach to the prevention of postoperative delirium in the elderly after gastrointestinal surgery. Surg Today 2002;**32**:310-314

19. Gupta K, Gupta A, Gupta PK, Rastogi B, Agarwal S, Lakhanpal M. Dexmedetomidine premedication in relevance to ketamine anesthesia: A prospective study. Anesth Essays Res 2011;**5**:87-91

20. He F, Shen L, Zhong J. A study of dexmedetomidine in the prevention of postoperative delirium in elderly patients after vertebral osteotomy. Int J Clin Exp Med 2018;**11**:4984-4990

30. Maldonado JR, Wysong A, Van Der Starre PJA, Block T, Miller C, Reitz BA. Dexmedetomidine and the reduction of postoperative delirium after cardiac surgery. Psychosomatics 2009;**50**:206-217

33. Mishina T, Aiba T, Hiramatsu K, Shibata Y, Yoshihara M, Aoba T, Yamaguchi N, Kato T. Comparison between dexmedetomidine and midazolam as a sedation agent with local anesthesia in inguinal hernia repair: randomized controlled trial. Hernia 2018;**22**:471-478

45. Wan L, Huang Q, Yue J, Lin L, Li S. Comparison of sedative effect of dexmedetomidine and midazolam for post-operative patients undergoing mechanical ventilation in surgical intensive care unit. Chinese Crit Care Med 2011:**23**:543-546

52. Yu D-N, Yi Zhu, Jue Ma, Sun Q. Comparison of post-anesthesia delirium in elderly patients treated with dexmedetomidine and midazolam maleate after thoracic surgery. Biomedical research (india) 2017;**28**:6852‐6855

86. Sultan SS. Assessment of role of perioperative melatonin in prevention and treatment of postoperative delirium after hip arthroplasty under spinal anesthesia in the elderly. Saudi J Anaesth 2010;**4**:169-173

**(5) Clonidine**

8. Rubino AS, Onorati F, Caroleo S, Galato E, Nucera S, Amantea B, Santini F, Renzulli A. Impact of clonidine administration on delirium and related respiratory weaning after surgical correction of acute type-A aortic dissection: Results of a pilot study. Interact Cardiovasc Thorac Surg 2010;**10**:58-62

86. Sultan SS. Assessment of role of perioperative melatonin in prevention and treatment of postoperative delirium after hip arthroplasty under spinal anesthesia in the elderly. Saudi J Anaesth 2010;**4**:169-73

**(6) Steroid**

68. Clemmesen CG, Lunn TH, Kristensen MT, Palm H, Foss NB. Effect of a single pre-operative 125 mg dose of methylprednisolone on postoperative delirium in hip fracture patients; a randomised, double-blind, placebo-controlled trial. Anaesthesia 2018;**73**:1353-1360

69. Whitlock RP, Devereaux PJ, Teoh KH, Lamy A, Vincent J, Pogue J, Paparella D, Sessler DI, Karthikeyan G, Villar JC, Zuo Y, Avezum A, Quantz M, Tagarakis GI, Shah PJ, Abbasi SH, Zheng H, Pettit S, Chrolavicius S, Yusuf S. Methylprednisolone in patients undergoing cardiopulmonary bypass (SIRS): a randomised, double-blind, placebo-controlled trial. Lancet 2015;**386**:1243-1253

81. Dieleman JM, Nierich AP, Rosseel PM, van der Maaten JM, Hofland J, Diephuis JC, Schepp RM, Boer C, Moons KG, van Herwerden LA, Tijssen JG, Numan SC, Kalkman CJ, van Dijk D. Intraoperative high-dose dexamethasone for cardiac surgery: a randomized controlled trial. JAMA 2012;**308**:1761-1767

82. Mardani D, Bigdelian H. The effect of dexamethasone prophylaxis on postoperative delirium after cardiac surgery: A randomized trial. Journal of research in medical sciences, 2012;**17**:S113-S1119

83. Šakić L, Tonković D, Godan BJ, Šakić K. The influence of dexamethasone administration in spinal anesthesia for femur fracture on postoperative cognitive dysfunction. Periodicum Biologorum 2015;**117**:281-285

84. Sauer AM, Slooter AJ, Veldhuijzen DS, van Eijk MM, Devlin JW, van Dijk D. Intraoperative dexamethasone and delirium after cardiac surgery: a randomized clinical trial. Anesth Analg 2014;**119**:1046-1052

**(7) Gabapentin, Pregabalin**

53. Dighe K, Clarke H, McCartney C, Wong C. Perioperative gabapentin and delirium following total knee arthroplasty: a post-hoc analysis of a double-blind randomized placebo-controlled trial Can J Anaesth 2014;**61**:1136-1137

54. Leung JM, Sands LP, Rico M, Petersen KL, Rowbotham MC, Dahl JB, Ames C, Chou D, Weinstein P. Pilot clinical trial of gabapentin to decrease postoperative delirium in older patients. Neurology 2006;**67**:1251-1253

55. Leung JM, Sands LP, Chen N, Ames C, Berven S, Bozic K, Burch S, Chou D, Covinsky K, Deviren V, Kinjo S, Kramer JH, Ries M, Tay B, Vail T, Weinstein P, Chang S, Meckler G, Newman S, Tsai T, Voss V, Youngblom E. Perioperative Gabapentin Does Not Reduce Postoperative Delirium in Older Surgical Patients: A Randomized Clinical Trial. Anesthesiology 2017;**127**:633-644

74. Farlinger C, Clarke H, Wong CL. Perioperative pregabalin and delirium following total hip arthroplasty: a post hoc analysis of a double-blind randomized placebo-controlled trial. Can J Anaesth 2018;**65**:1269-1270

**(8) Haloperidol**

56. Fukata S, Kawabata Y, Fujishiro K, Kitagawa Y, Kuroiwa K, Akiyama H, Takemura M, Ando M, Hattori H. Haloperidol prophylaxis for preventing aggravation of postoperative delirium in elderly patients: a randomized, open-label prospective trial. Surgery Today 2016;**47**:815-826

57. Kalisvaart KJ, De Jonghe JFM, Bogaards MJ, Vreeswijk R, Egberts TCG, Burger BJ, Eikelenboom P, Van Gool WA. Haloperidol prophylaxis for elderly hip-surgery patients at risk for delirium: A randomized placebo-controlled study. J Am Geriatr Soc 2005;**53**:1658-1666

58. Kaneko T, Cai J, Ishikura T, Kobayashi M, Naka T, Kaibara N. Prophylactic consecutive administration of haloperidol can reduce the occurrence of postoperative delirium in gastrointestinal surgery Yonago acta medica, 1999;**43**:179-184

59. Khan BA, Perkins AJ, Campbell NL, Gao S, Khan SH, Wang S, Fuchita M, Weber DJ, Zarzaur BL, Boustani MA, Kesler K. Preventing Postoperative Delirium After Major Noncardiac Thoracic Surgery-A Randomized Clinical Trial. J Am Geriatr Soc 2018;**66**:2289-2297

60. Wang W, Li H, Wang D, Zhu X, Li S, Yao G, Chen K, Gu X, Zhu S. Haloperidol prophylaxis decreases delirium incidence in elderly patients after noncardiac surgery: a randomized controlled trial. Crit Care Med 2012;**40**:731-739

**(9) Ketamine**

29. Ma PP, Piao MH, Wang YS, Ma HC, Feng CS. Influence of dexmedetomidine and sub-anesthetic dose of ketamine on postoperative delirium in elderly orthopedic patients under total intravenous anesthesia. Journal of Jilin University Medicine Edition 2013;**39**:128-132

61. Avidan MS, Maybrier HR, Abdallah AB, Jacobsohn E, Vlisides PE, Pryor KO, Veselis RA, Grocott HP, Emmert DA, Rogers EM, Downey RJ, Yulico H, Noh GJ, Lee YH, Waszynski CM, Arya VK, Pagel PS, Hudetz JA, Muench MR, Fritz BA, Waberski W, Inouye SK, Mashour GA. Intraoperative ketamine for prevention of postoperative delirium or pain after major surgery in older adults: An international, multicentre, double-blind, randomised clinical trial. Lancet 2017; **390**: 267-275

62. Du J, Huang YG, Yu XR, Zhao N. Effects of preoperative ketamine on the endocrine-metabolic and inflammatory response to laparoscopic surgery. Chin Med J 2011;**124**:3721-3725

63. Gecaj-Gashi A, Hashimi M, Sada F, Salihu S, Terziqi H. Prophylactic ketamine reduces incidence of postanaesthetic shivering. Niger J Med 2010;**19**:267-270

64. Hudetz JA, Patterson KM, Iqbal Z, Gandhi SD, Byrne AJ, Hudetz AG, Warltier DC, Pagel PS. Ketamine Attenuates Delirium After Cardiac Surgery With Cardiopulmonary Bypass. J Cardiothorac Vasc Anesth 2009;**23**:651-657

**(10) Lidocaine**

9. Andjelkovic L, Novak-Jankovic V, Pozar-Lukanovic N, Bosnic Z, Spindler-Vesel A. Influence of dexmedetomidine and lidocaine on perioperative opioid consumption in laparoscopic intestine resection: a randomized controlled clinical trial. J Int Med Res 2018;**46**:5143‐5154

65. Dewinter G, Moens P, Fieuws S, Vanaudenaerde B, Van De Velde M, Rex S. Systemic lidocaine fails to improve postoperative morphine consumption, postoperative recovery and quality of life in patients undergoing posterior spinal arthrodesis. A double-blind, randomized, placebo-controlled trial. Br J Anaesth 2017;**118**:576‐585

**(11) Melatonin**

66. de Jonghe A, van Munster BC, Goslings JC, Kloen P, van Rees C, Wolvius R, van Velde R, Levi M, de Haan RJ, de Rooij SE. Effect of melatonin on incidence of delirium among patients with hip fracture: a multicentre, double-blind randomized controlled trial. CMAJ 2014;**186**:E547-556

67. Yamaguchi Y, Mihara T, Taguri M, Yamaguchi O, Goto T. Melatonin receptor agonist for the prevention of postoperative delirium in elderly patients: A randomized, double-blind, placebo-controlled trial. Intensive care medicine, 2014:**40**:S246

85. Nickkholgh A, Schneider H, Sobirey M, Venetz WP, Hinz U, Pelzl LH, Gotthardt DN, Cekauskas A, Manikas M, Mikalauskas S, Mikalauskene L, Bruns H, Zorn M, Weigand MA, Büchler MW, Schemmer P. The use of high-dose melatonin in liver resection is safe: First clinical experience. J Pineal Res 2011;**50**:381-388

86. Sultan SS. Assessment of role of perioperative melatonin in prevention and treatment of postoperative delirium after hip arthroplasty under spinal anesthesia in the elderly. Saudi J Anaesth 2010;**4**:169-173

**(12) Atypical Antipsychotics (Olanzapine, Risperidone)**

71. Larsen KA, Kelly SE, Stern TA, Bode Jr RH, Price LL, Hunter DJ, Gulczynski D, Bierbaum BE, Sweeney GA, Hoikala KA, Cotter JJ, Potter AW. Administration of olanzapine to prevent postoperative delirium in elderly joint-replacement patients: A randomized, controlled trial. Psychosomatics 2010;**51**:409-418

78. Hakim S, Othman A, Naoum D. Early treatment with risperidone for subsyndromal delirium after on-pump cardiac surgery in the elderly: a randomized trial Anesthesiology 2012;**116**:987-997

79. Prakanrattana U, Prapaitrakool S. Efficacy of risperidone for prevention of postoperative delirium in cardiac surgery. Anaesth Intensive Care 2007;**35**:714-719

**(13) Propofol**

13. Chang YF, Chao A, Shih PY, Hsu YC, Lee CT, Tien YW, Yeh YC, Chen LW. Comparison of dexmedetomidine versus propofol on hemodynamics in surgical critically ill patients. J Surg Res 2018;**228**:194-200

15. Corbett SM, Rebuck JA, Greene CM, Callas PW, Neale BW, Healey MA, Leavitt BJ. Dexmedetomidine does not improve patient satisfaction when compared with propofol during mechanical ventilation. Crit Care Med 2005;**33**:940-945

17. Djaiani G, Silverton N, Fedorko L, Carroll J, Styra R, Rao V, Katznelson R. Dexmedetomidine versus Propofol Sedation Reduces Delirium after Cardiac Surgery: A Randomized Controlled Trial. Anesthesiology 2016;**124**:362-368

21. Huang F, Wang J, Yang X, Xu H, Kong J, Liu S, Jin J. Sedative effects of dexmedetomidine in post-operative elder patients on mechanical ventilation. Natl Med J China 2014;**94**:3211-3215

27. Liu X, Zhang K, Wang W, Xie G, Cheng B, Wang Y, Hu Y, Fang X. Dexmedetomidine Versus Propofol Sedation Improves Sublingual Microcirculation After Cardiac Surgery: a Randomized Controlled Trial. J Cardiothorac Vasc Anesth 2016;**30**:1509-1515

30. Maldonado JR, Wysong A, Van Der Starre PJA, Block T, Miller C, Reitz BA. Dexmedetomidine and the reduction of postoperative delirium after cardiac surgery. Psychosomatics 2009;**50**:206-217

32. Mei B, Meng G, Xu G, Cheng X, Chen S, Zhang Y, Zhang M, Liu X, Gu E. Intraoperative Sedation With Dexmedetomidine is Superior to Propofol for Elderly Patients Undergoing Hip Arthroplasty: A Prospective Randomized Controlled Study. Clin J Pain 2018;**34**:811-817

35. Susheela AT, Packiasabapathy S, Gasangwa DV, Patxot M, O'Neal J, Marcantonio E, Subramaniam B. The use of dexmedetomidine and intravenous acetaminophen for the prevention of postoperative delirium in cardiac surgery patients over 60 years of age: a pilot study. F1000Res 2017;**6**:1842

39. Sheikh TA, Dar BA, Akhter N, Ahmad N. A Comparative Study Evaluating Effects of Intravenous Sedation by Dexmedetomidine and Propofol on Patient Hemodynamics and Postoperative Outcomes in Cardiac Surgery. Anesth Essays Res 2018;**12**:555-560

75. Royse CF, Andrews DT, Newman SN, Stygall J, Williams Z, Pang J, Royse AG. The influence of propofol or desflurane on postoperative cognitive dysfunction in patients undergoing coronary artery bypass surgery. Anaesthesia 2011;**66**:455-464

76. Tanaka P, Goodman S, Sommer B, Maloney W, Huddleston J, Lemmens H. The effect of desflurane versus propofol anesthesia on postoperative delirium in elderly obese patients undergoing total knee replacement: a randomized, controlled, double-blinded clinical trial. J Clin Anesth 2017;**39**:17-22

77. Moscarelli M, Terrasini N, Nunziata A, Punjabi P, Angelini G, Solinas M, Buselli A, Sarto PD, Haxhiademi D. A Trial of Two Anesthetic Regimes for Minimally Invasive Mitral Valve Repair. J Cardiothorac Vasc Anesth 2018;**32**:2562-2569

80. Subramaniam B, Shankar P, Shaefi S, Mueller A, O'Gara B, Banner-Goodspeed V, Gallagher J, Gasangwa D, Patxot M, Packiasabapathy S, Mathur P, Eikermann M, Talmor D, Marcantonio ER. Effect of Intravenous Acetaminophen vs Placebo Combined With Propofol or Dexmedetomidine on Postoperative Delirium Among Older Patients Following Cardiac Surgery: The DEXACET Randomized Clinical Trial. JAMA 2019;**321**:686-696

**(14) Nimodipine**

70. Li YN, Zhang Q, Yin CP, Guo YY, Huo SP, Wang L, Wang QJ. Effects of nimodipine on postoperative delirium in elderly under general anesthesia: A prospective, randomized, controlled clinical trial. Medicine (Baltimore) 2017;**96**:e6849

**(15) Ondansetron**

72. Papadopoulos G, Pouangare M, Papathanakos G, Arnaoutoglou E, Petrou A, Tzimas P. The effect of ondansetron on postoperative delirium and cognitive function in aged orthopedic patients. Minerva Anestesiol 2014;**80**:444-451

**(16) Parecoxib**

73. Mu DL, Zhang DZ, Wang DX, Wang G, Li CJ, Meng ZT, Li YW, Liu C, Li XY. Parecoxib supplementation to morphine analgesia decreases incidence of delirium in elderly patients after hip or knee replacement surgery: A randomized controlled trial. Anesth Analg 2017;**124**:1992-2000

**(17) Opioid**

36. Priye S, Jagannath S, Singh D, Shivaprakash S, Reddy DP. Dexmedetomidine as an adjunct in postoperative analgesia following cardiac surgery: A randomized, double-blind study. Saudi J Anaesth 2015;**9**:353-358

38. Shehabi Y, Grant P, Wolfenden H, Hammond N, Bass F, Campbell M, Chen J. Prevalence of delirium with dexmedetomidine compared with morphine based therapy after cardiac surgery: A randomized controlled trial (DEXmedetomidine compared to morphine-DEXCOM study). Anesthesiology 2009;**111**:1075-1084

**(18) Volatile anesthetics**

75. Royse CF, Andrews DT, Newman SN, Stygall J, Williams Z, Pang J, Royse AG. The influence of propofol or desflurane on postoperative cognitive dysfunction in patients undergoing coronary artery bypass surgery. Anaesthesia 2011;**66**:455-464

76. Tanaka P, Goodman S, Sommer B, Maloney W, Huddleston J, Lemmens H. The effect of desflurane versus propofol anesthesia on postoperative delirium in elderly obese patients undergoing total knee replacement: a randomized, controlled, double-blinded clinical trial. J Clinical Anesthesia, 2017;**39**:17-22

77. Moscarelli M, Terrasini N, Nunziata A, Punjabi P, Angelini G, Solinas M, Buselli A, Sarto PD, Haxhiademi D. A Trial of Two Anesthetic Regimes for Minimally Invasive Mitral Valve Repair. J Cardiothorac Vasc Anesth 2018;**32**:2562-2569

**Supplemental Text S2**. Credibility assessment of network meta-regression and subgroup analyses using the Instrument for assessing the Credibility of Effect Modification Analyses (ICEMAN) tool.

**(1) Network meta-regression**

1: Is the analysis of effect modification based on comparison within rather than between trials?

[ ] Completely between [0] Mostly between or unclear [ ] Mostly within [ ] Completely within

2: For within-trial comparisons, is the effect modification similar from trial to trial?

[ ] Not applicable: no or one within-RCT comparison [ ] Definitely not similar [0] Probably not similar or unclear [ ] Mostly similar [ ] Definitely similar

3. For between-trial comparisons, is the number of trials large?

[ ] Not applicable: no between RCT comparison [ ] Very small [0] Rather small or unclear [ ] Rather large [ ] Large

4. Was the direction of effect modification correctly hypothesized a priori?

[ ] Definitely no [ ] Probably no or unclear [ ] Probably yes [0] Definitely yes

5. Does a test for interaction suggest that chance is an unlikely explanation of the apparent effect modification? (consider irrespective of number of effect modifiers)

[ ] Chance a very likely explanation [ ] Chance a likely explanation or unclear [ ] Chance may not explain [0] Chance an unlikely explanation

6: Did the authors test only a small number of effect modifiers or consider the number in their statistical analysis?

[ ] Definitely no [ ] Probably no or unclear [0] Probably yes [ ] Definitely yes

7. Did the authors use a random effects model?

[ ] Definitely no [ ] Probably no or unclear [ ] Probably yes [0] Definitely yes

8. If the effect modifier is a continuous variable, were arbitrary cut points avoided?

[ ] not applicable: not continuous [ ] Definitely no [ ] Probably no or unclear [ ] Probably yes [0] Definitely yes

9. Optional: Are there any additional considerations that may increase or decrease credibility? (manual section 3.9) [0] not applicable [ ] Yes, probably decrease [ ] Yes, probably increase

**(2) Subgroup analysis – Cardiac vs. Non-cardiac surgeries**

1: Is the analysis of effect modification based on comparison within rather than between trials?

[ ] Completely between [0] Mostly between or unclear [ ] Mostly within [ ] Completely within

2: For within-trial comparisons, is the effect modification similar from trial to trial?

[ ] Not applicable: no or one within-RCT comparison [0] Definitely not similar [ ] Probably not similar or unclear [ ] Mostly similar [ ] Definitely similar

3. For between-trial comparisons, is the number of trials large?

[ ] Not applicable: no between RCT comparison [ ] Very small [0] Rather small or unclear [ ] Rather large [ ] Large

4. Was the direction of effect modification correctly hypothesized a priori?

[ ] Definitely no [ ] Probably no or unclear [ ] Probably yes [0] Definitely yes

5. Does a test for interaction suggest that chance is an unlikely explanation of the apparent effect modification? (consider irrespective of number of effect modifiers)

[ ] Chance a very likely explanation [ ] Chance a likely explanation or unclear [ ] Chance may not explain [0] Chance an unlikely explanation

6: Did the authors test only a small number of effect modifiers or consider the number in their statistical analysis?

[ ] Definitely no [ ] Probably no or unclear [0] Probably yes [ ] Definitely yes

7. Did the authors use a random effects model?

[ ] Definitely no [ ] Probably no or unclear [ ] Probably yes [0] Definitely yes

8. If the effect modifier is a continuous variable, were arbitrary cut points avoided?

[ ] not applicable: not continuous [ ] Definitely no [ ] Probably no or unclear [ ] Probably yes [0] Definitely yes

9. Optional: Are there any additional considerations that may increase or decrease credibility? (manual section 3.9) [0] not applicable [ ] Yes, probably decrease [ ] Yes, probably increase

**(3) Subgroup analysis – trials using CAM or CAM-ICU criteria**

1: Is the analysis of effect modification based on comparison within rather than between trials?

[ ] Completely between [0] Mostly between or unclear [ ] Mostly within [ ] Completely within

2: For within-trial comparisons, is the effect modification similar from trial to trial?

[ ] Not applicable: no or one within-RCT comparison [ ] Definitely not similar [ ] Probably not similar or unclear [0] Mostly similar [ ] Definitely similar

3. For between-trial comparisons, is the number of trials large?

[ ] Not applicable: no between RCT comparison [ ] Very small [0] Rather small or unclear [ ] Rather large [ ] Large

4. Was the direction of effect modification correctly hypothesized a priori?

[ ] Definitely no [ ] Probably no or unclear [ ] Probably yes [0] Definitely yes

5. Does a test for interaction suggest that chance is an unlikely explanation of the apparent effect modification? (consider irrespective of number of effect modifiers)

[ ] Chance a very likely explanation [ ] Chance a likely explanation or unclear [ ] Chance may not explain [0] Chance an unlikely explanation

6: Did the authors test only a small number of effect modifiers or consider the number in their statistical analysis?

[ ] Definitely no [ ] Probably no or unclear [0] Probably yes [ ] Definitely yes

7. Did the authors use a random effects model?

[ ] Definitely no [ ] Probably no or unclear [ ] Probably yes [0] Definitely yes

8. If the effect modifier is a continuous variable, were arbitrary cut points avoided?

[ ] not applicable: not continuous [0] Definitely no [ ] Probably no or unclear [ ] Probably yes [ ] Definitely yes

9. Optional: Are there any additional considerations that may increase or decrease credibility? (manual section 3.9) [0] not applicable [ ] Yes, probably decrease [ ] Yes, probably increase

**(4) Subgroup analysis – Old age group**

1: Is the analysis of effect modification based on comparison within rather than between trials?

[ ] Completely between [0] Mostly between or unclear [ ] Mostly within [ ] Completely within

2: For within-trial comparisons, is the effect modification similar from trial to trial?

[ ] Not applicable: no or one within-RCT comparison [0] Definitely not similar [ ] Probably not similar or unclear [ ] Mostly similar [ ] Definitely similar

3. For between-trial comparisons, is the number of trials large?

[ ] Not applicable: no between RCT comparison [ ] Very small [0] Rather small or unclear [ ] Rather large [ ] Large

4. Was the direction of effect modification correctly hypothesized a priori?

[ ] Definitely no [ ] Probably no or unclear [ ] Probably yes [0] Definitely yes

5. Does a test for interaction suggest that chance is an unlikely explanation of the apparent effect modification? (consider irrespective of number of effect modifiers)

[ ] Chance a very likely explanation [ ] Chance a likely explanation or unclear [ ] Chance may not explain [0] Chance an unlikely explanation

6: Did the authors test only a small number of effect modifiers or consider the number in their statistical analysis?

[ ] Definitely no [ ] Probably no or unclear [0] Probably yes [ ] Definitely yes

7. Did the authors use a random effects model?

[ ] Definitely no [ ] Probably no or unclear [ ] Probably yes [0] Definitely yes

8. If the effect modifier is a continuous variable, were arbitrary cut points avoided?

[ ] not applicable: not continuous [ ] Definitely no [0] Probably no or unclear [ ] Probably yes [ ] Definitely yes

9. Optional: Are there any additional considerations that may increase or decrease credibility? (manual section 3.9) [0] not applicable [ ] Yes, probably decrease [ ] Yes, probably increase

**Supplemental Text S3**. Search strategies (updated on 20/January/2021).

**Pubmed**

Option: all fields

1. (Delirium) OR (Confusion) OR (Disorientation) OR (Bewilderment)

2. (Postoperative) OR (Surgery) OR (Surgical) OR (Perioperative) OR (Operation) OR (Postsurgical) OR (Post Surgical) OR (Anesthesia recovery period) OR (Anaesthesia recovery period) OR (Postanesthesia) OR (Postanaesthesia)

3. (Morphine) OR (Pethidine) OR (Dexamethasone) OR (Clonidine) OR (Dexmedetomidine) OR (Morphine) OR (Ketamine) OR (Propofol) OR (Midazolam) OR (Diazepam) OR (Risperidone) OR (Olanzapine) OR (Quetiapine) OR (Ziprasidone) OR (Rivastigmine) OR (Melatonin) OR (Haloperidol) OR (Benzodiazepine) OR (Antipsychotics) OR (Gabapentin) OR (Pregabalin) OR (Cimetidine) OR (Ranitidine) OR (Donepezil) OR (Rivastigmine) OR (Cholinesterase inhibitor)

4. (Randomized controlled trial) OR (controlled clinical trial) OR (Randomized) OR (Randomised) OR (Placebo) OR (Controlled) OR (Random*) OR (Trial) OR (Blind* OR Mask*)

5. 1 AND 2 AND 3 AND 4 (592)

**Embase**

Option: all fields

1. (Delirium) OR (Confusion) OR (Disorientation) OR (Bewilderment)

2. (Postoperative) OR (Surgery) OR (Surgical) OR (Perioperative) OR (Operation) OR (Postsurgical) OR (Post Surgical) OR (Anesthesia recovery period) OR (Anaesthesia recovery period) OR (Postanesthesia) OR (Postanaesthesia)

3. (Morphine) OR (Pethidine) OR (Dexamethasone) OR (Clonidine) OR (Dexmedetomidine) OR (Morphine) OR (Ketamine) OR (Propofol) OR (Midazolam) OR (Diazepam) OR (Risperidone) OR (Olanzapine) OR (Quetiapine) OR (Ziprasidone) OR (Rivastigmine) OR (Melatonin) OR (Haloperidol) OR (Benzodiazepine) OR (Antipsychotics) OR (Gabapentin) OR (Pregabalin) OR (Cimetidine) OR (Ranitidine) OR (Donepezil) OR (Rivastigmine) OR (Cholinesterase inhibitor)

4. (Randomized controlled trial) OR (controlled clinical trial) OR (Randomized) OR (Randomised) OR (Placebo) OR (Controlled) OR (Random*) OR (Trial) OR (Blind* OR Mask*)

5. 1 AND 2 AND 3 AND 4 (2448)

Filter:

Age: young adult, adult, middle aged, aged, very elderly

Study types: human, controlled study, randomized controlled trial, major clinical study, clinical trial, double blind procedure, controlled clinical trial, prospective study, clinical article, comparative study, randomized controlled trial topic, comparative effectiveness, single blind procedure, drug dose comparison, intermethod comparison, clinical study, multicenter study (2305)

Publication types: article, article in press (795)

**Cochrane central register** of Controlled Trials [Central, Issue 1 of 2020]

Option: title, abstract, keywords

1. (Delirium) OR (Confusion) OR (Disorientation) OR (Bewilderment)

2. (Postoperative) OR (Surgery) OR (Surgical) OR (Perioperative) OR (Operation) OR (Postsurgical) OR (Post Surgical) OR (Anesthesia recovery period) OR (Anaesthesia recovery period) OR (Postanesthesia) OR (Postanaesthesia)

3. (Morphine) OR (Pethidine) OR (Dexamethasone) OR (Clonidine) OR (Dexmedetomidine) OR (Morphine) OR (Ketamine) OR (Propofol) OR (Midazolam) OR (Diazepam) OR (Risperidone) OR (Olanzapine) OR (Quetiapine) OR (Ziprasidone) OR (Rivastigmine) OR (Melatonin) OR (Haloperidol) OR (Benzodiazepine) OR (Antipsychotics) OR (Gabapentin) OR (Pregabalin) OR (Cimetidine) OR (Ranitidine) OR (Donepezil) OR (Rivastigmine) OR (Cholinesterase inhibitor)

4. (Randomized controlled trial) OR (controlled clinical trial) OR (Randomized) OR (Randomised) OR (Placebo) OR (Controlled) OR (Random*) OR (Trial) OR (Blind* OR Mask*)

5. 1 AND 2 AND 3 AND 4 (845)

6. Option: Trials (581)

**Supplemental Figure S1.** Loop-specific inconsistency plot.


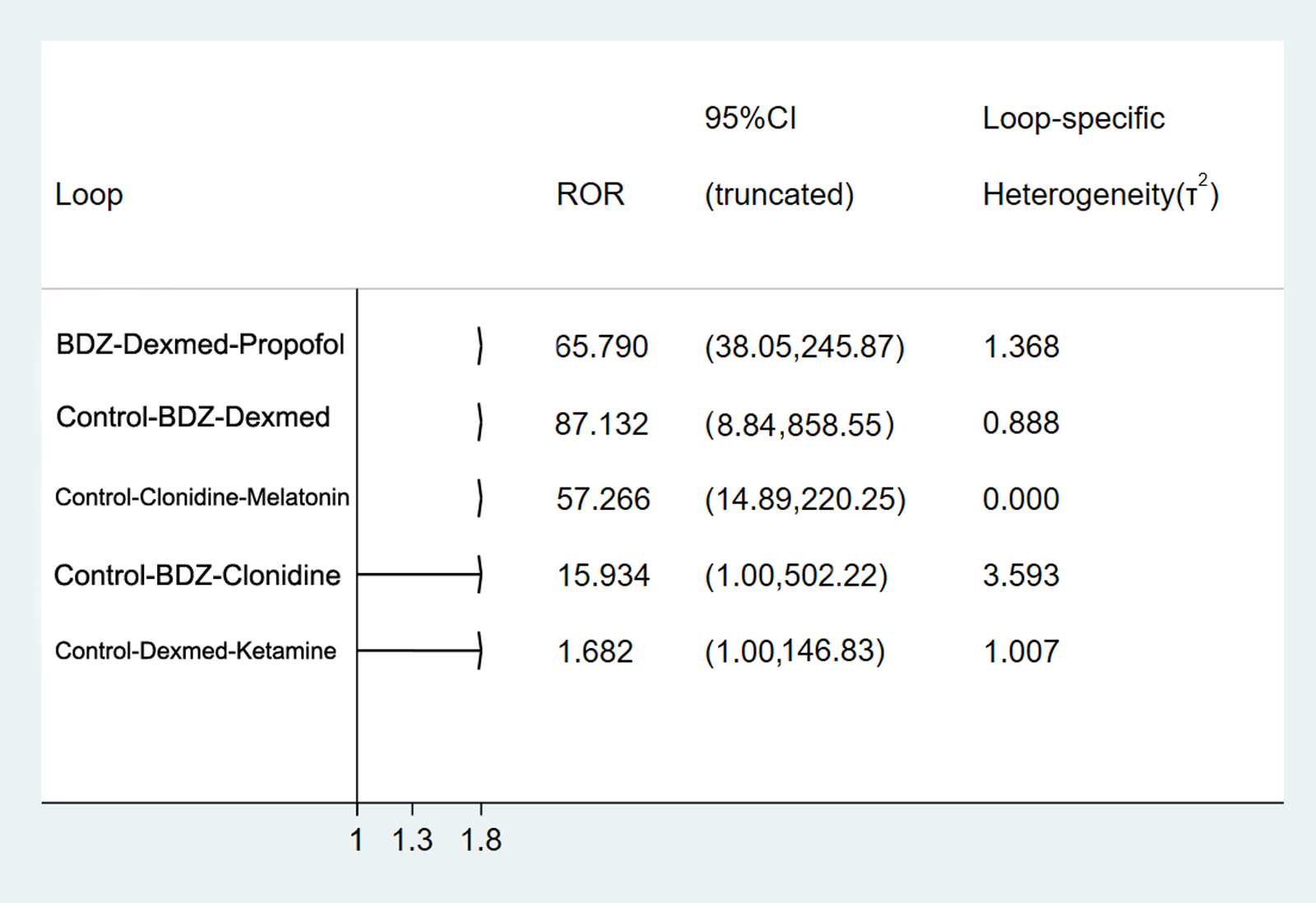


**Supplemental Figure S2.** Exploratory meta-regression plot regarding patient age, the proportion of male gender. The results showed that none of the regression coefficients of the meta-regression turned out to be statistically insignificant regarding the incidence of delirium (proportion of male: r= -0.003, 95% CI -0.02 to 0.01, *P*= 0.628; Age: r= -0.007, 95% CI -0.03 to 0.02, *P*= 556).


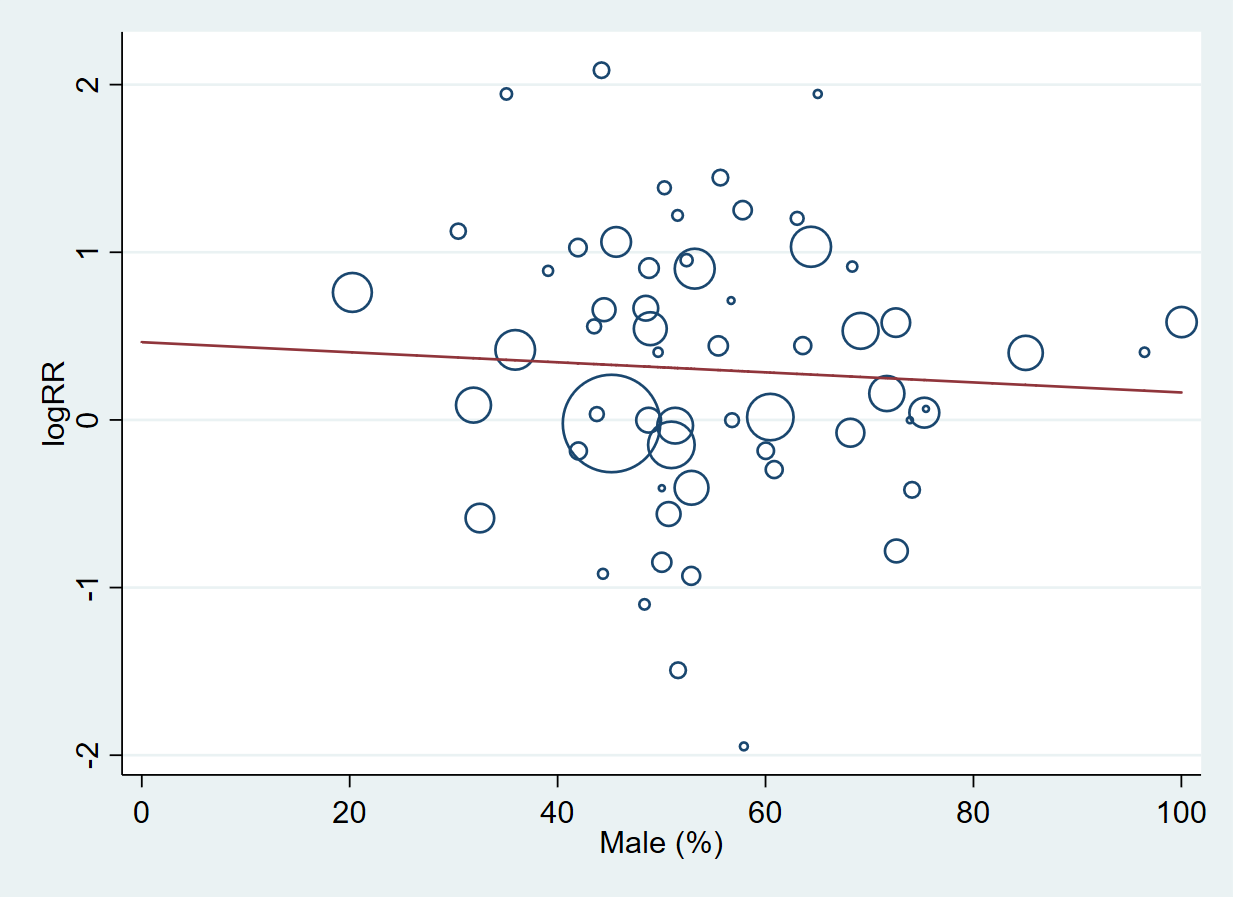

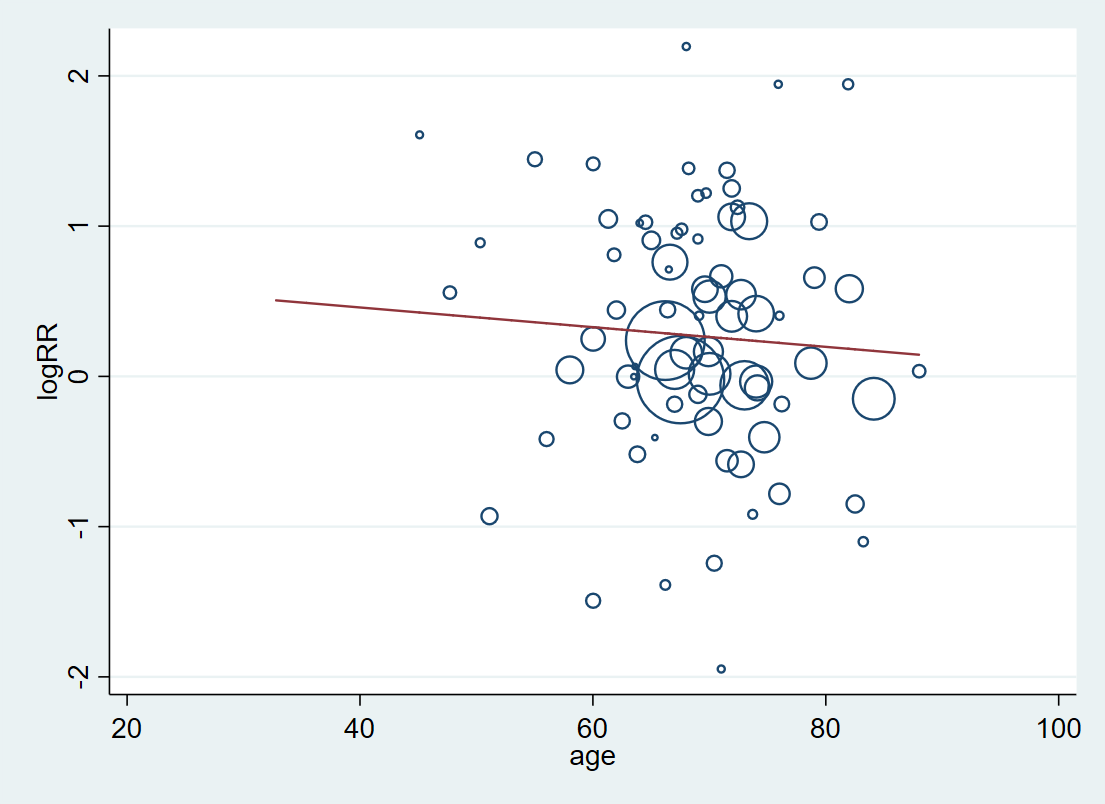


**Supplemental Figure S3.** Cumulative ranking plots of the pharmacologic agents to prevent delirium.


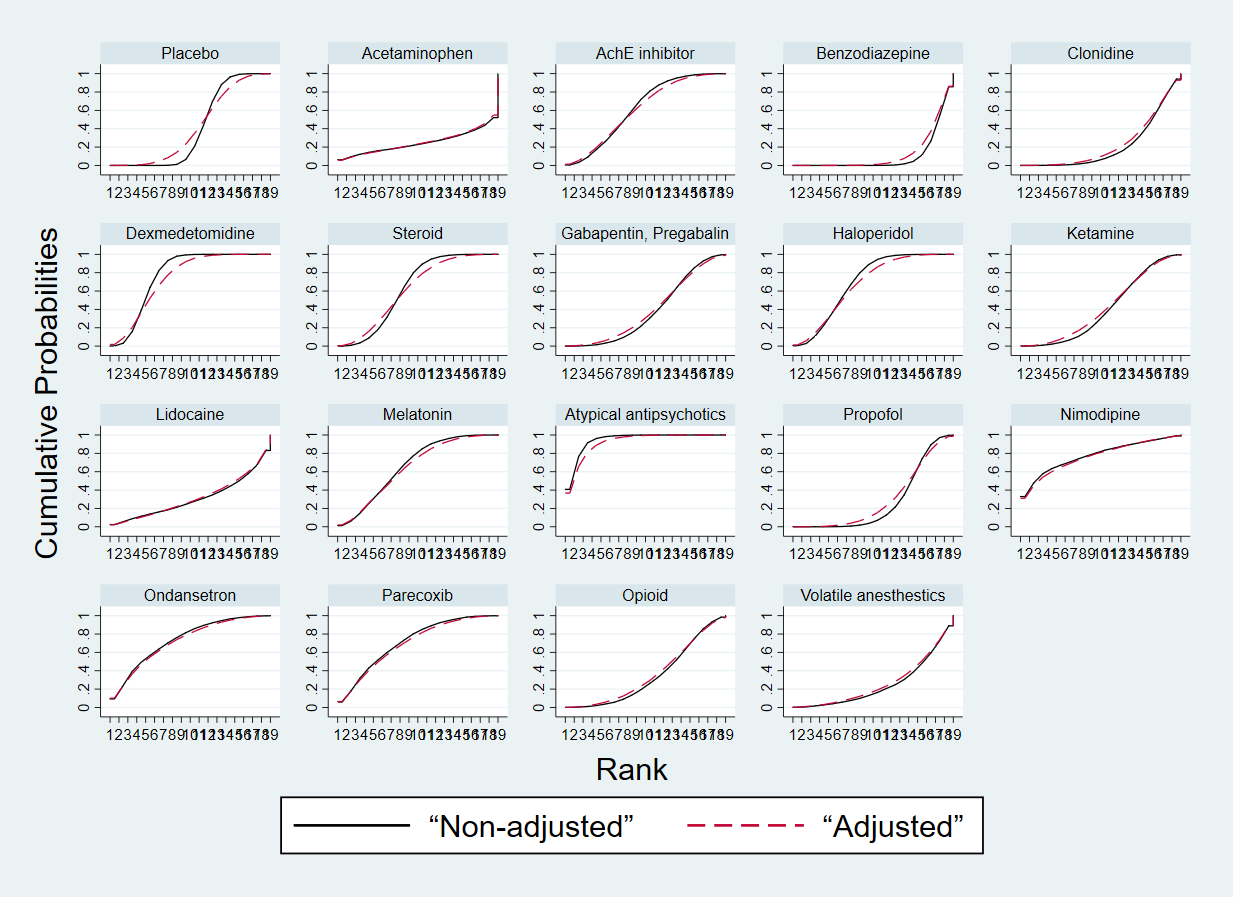


**Supplemental Figure S4**. Rankogram of the pharmacologic agents to prevent delirium.


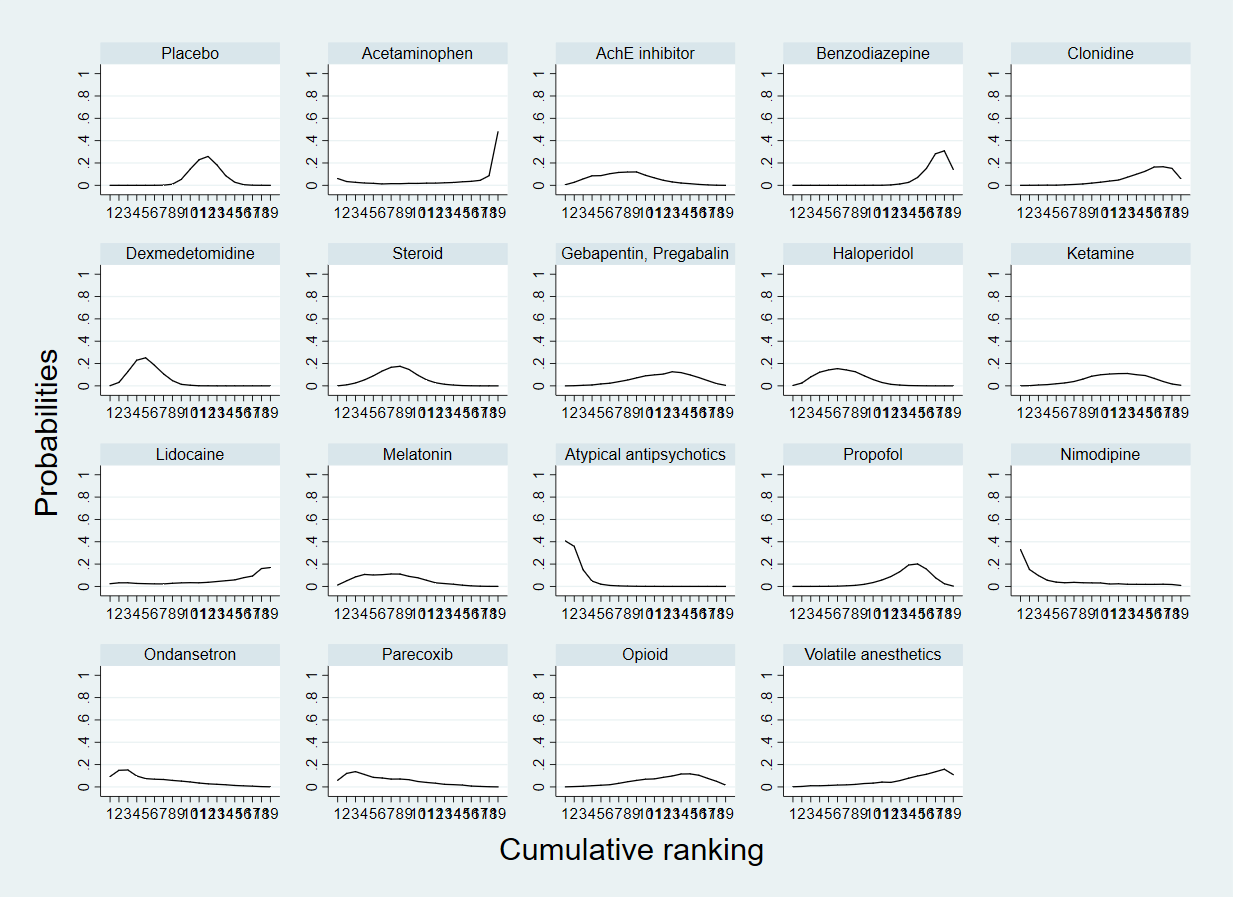


**Supplemental Figure S5**. Comparison-adjusted funnel plots for assessing small-study effects.


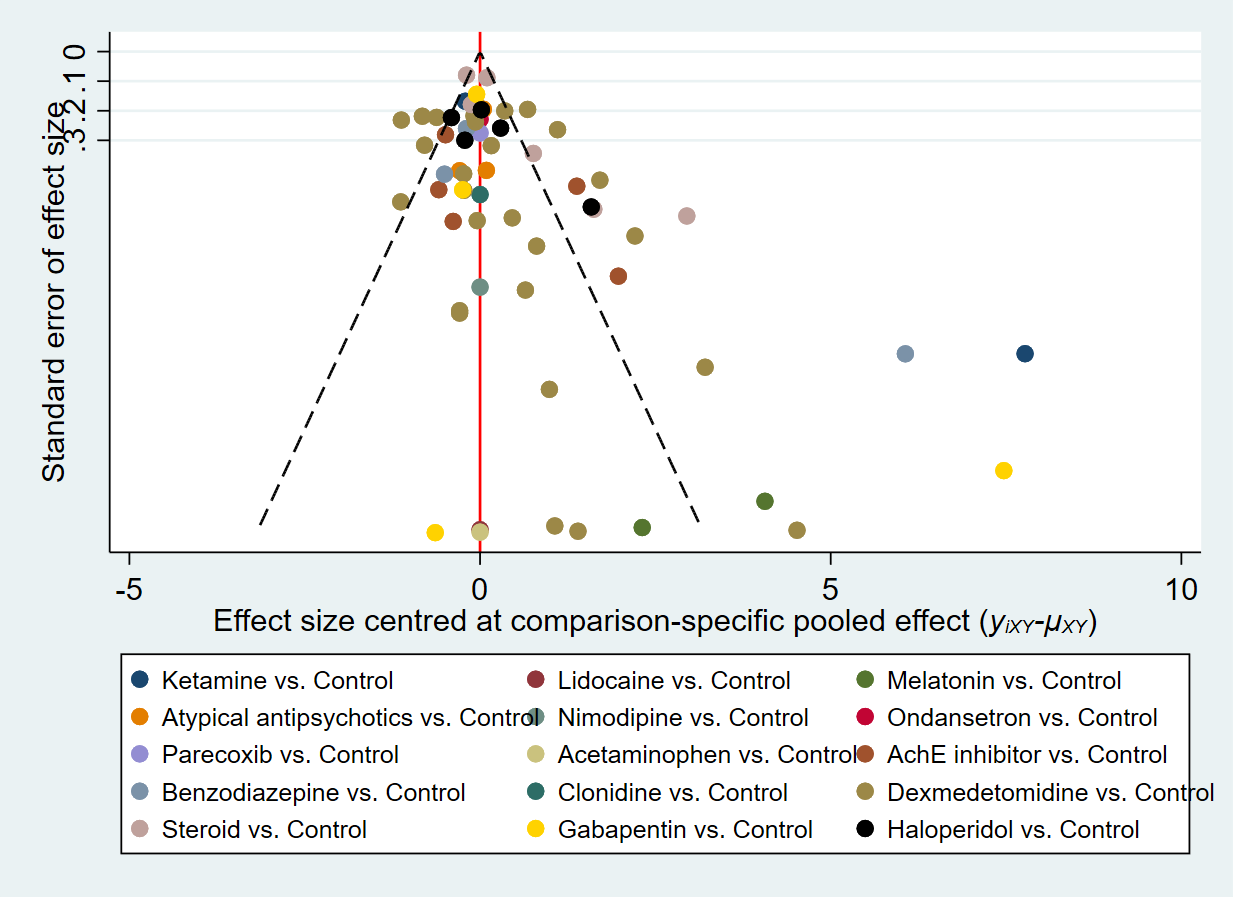


**Supplemental Figure S6**. Assessment of risk of bias in all included individual studies and their summary.

(part 2) (part 1)


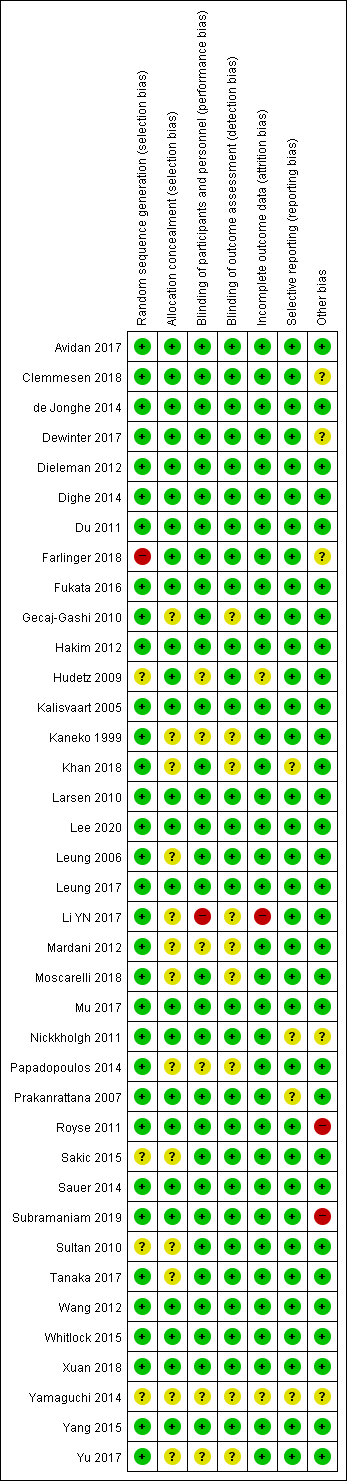

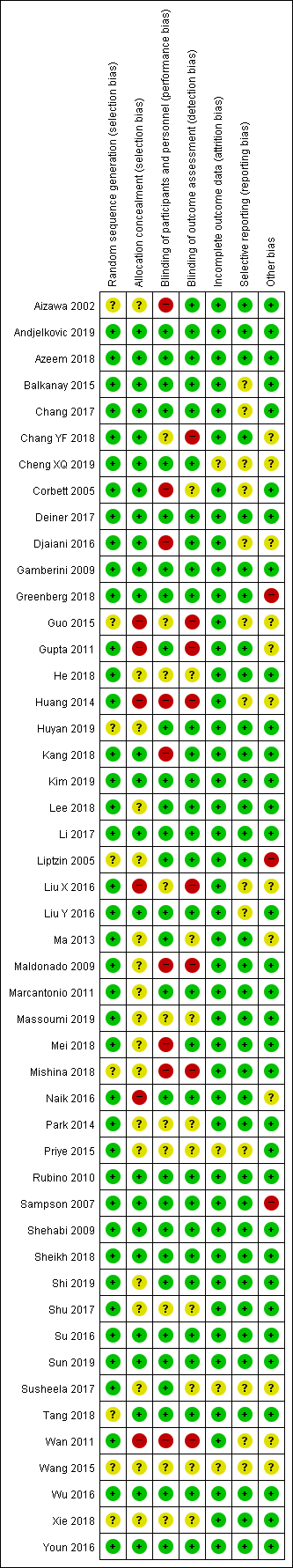

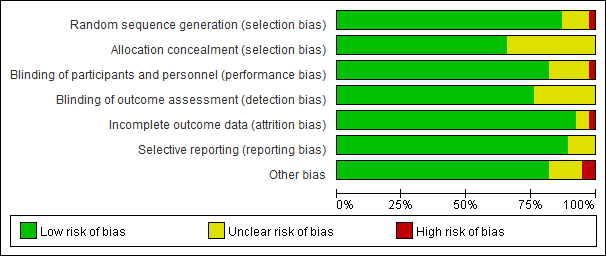


**Supplemental Figure S7**. Network plot of our network of postoperative delirium in the subgroup of patients receiving cardiac surgery.


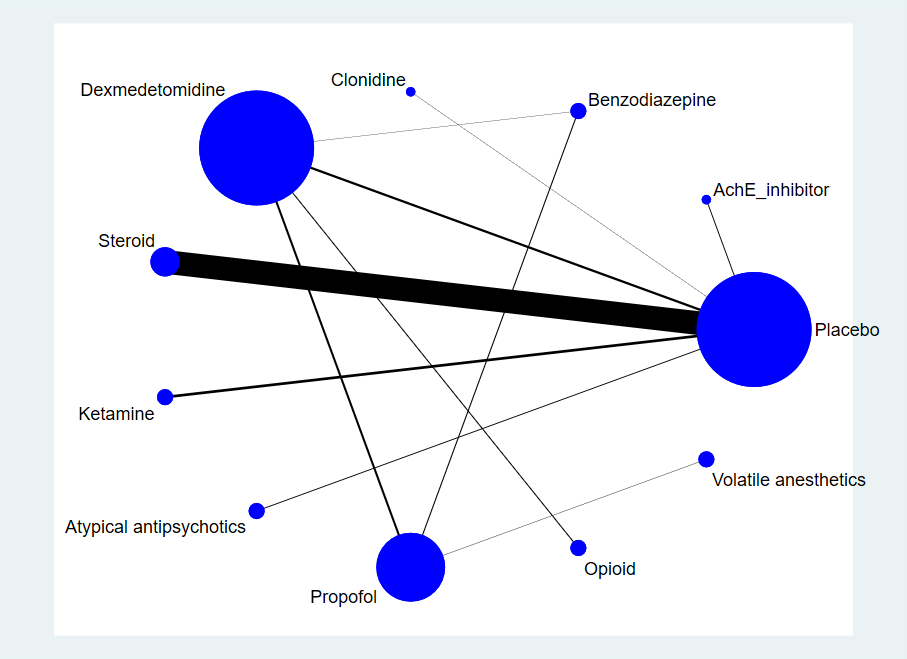


**Supplemental Figure S8**. Cumulative ranking plots of the pharmacologic agents in the subgroup of patients receiving cardiac surgery.
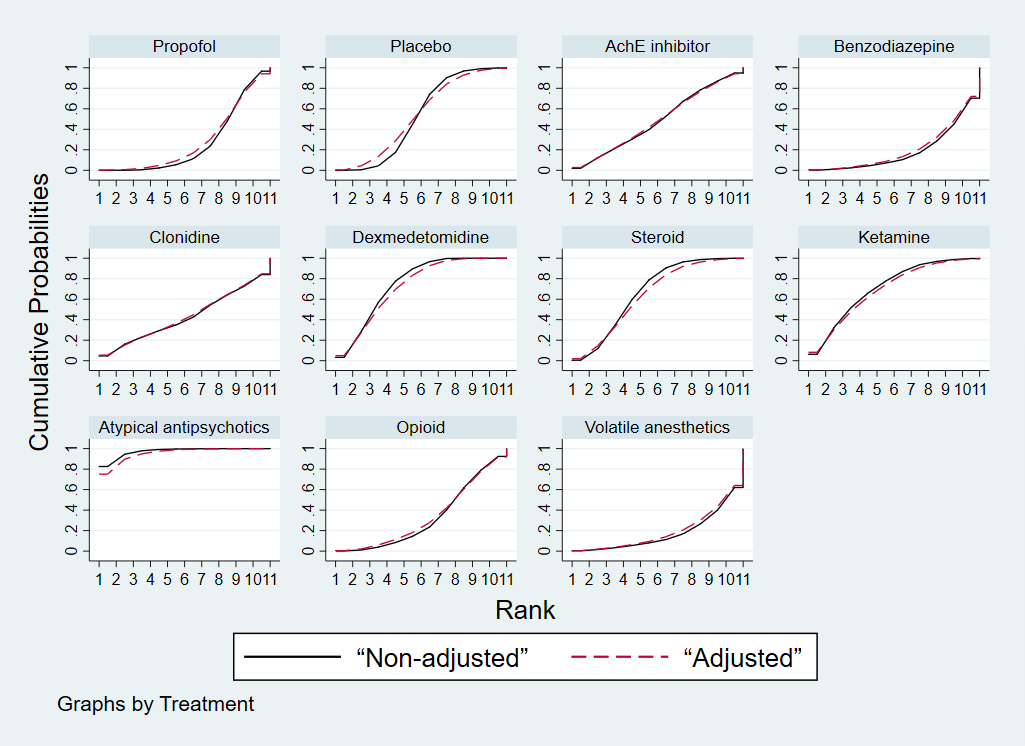


**Supplemental Figure S9**. Relative ranking plot of the incidence of postoperative delirium in the subgroup of patients receiving cardiac surgery.


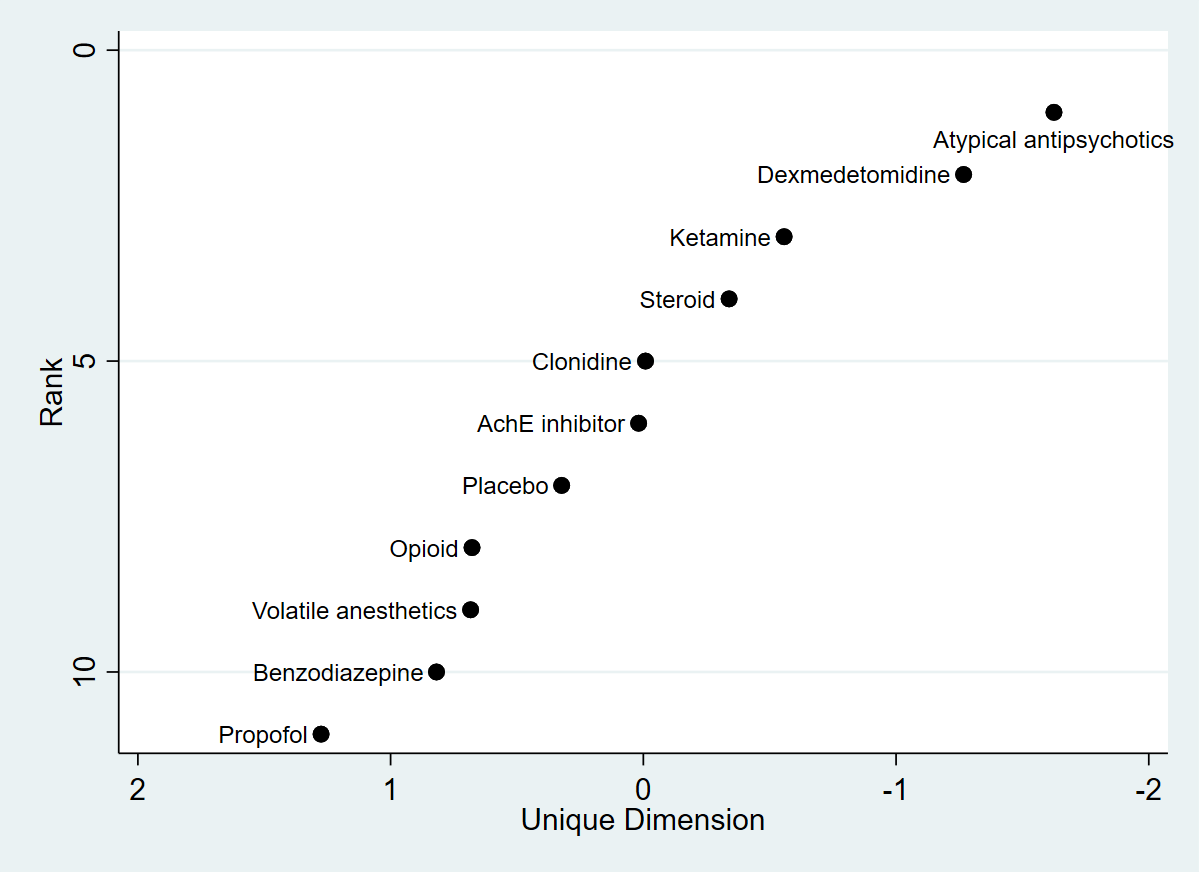


**Supplemental Figure S10**. Network plot of our network of postoperative delirium in the subgroup of patients receiving non-cardiac surgery.


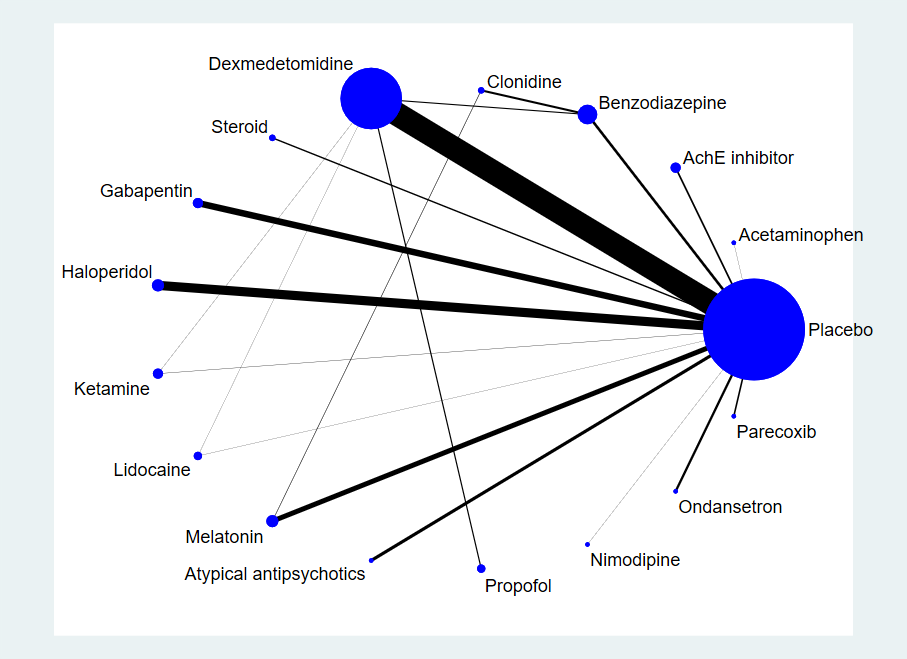


**Supplemental Figure S11**. Cumulative ranking plots of the pharmacologic agents in the subgroup of patients receiving non-cardiac surgery.


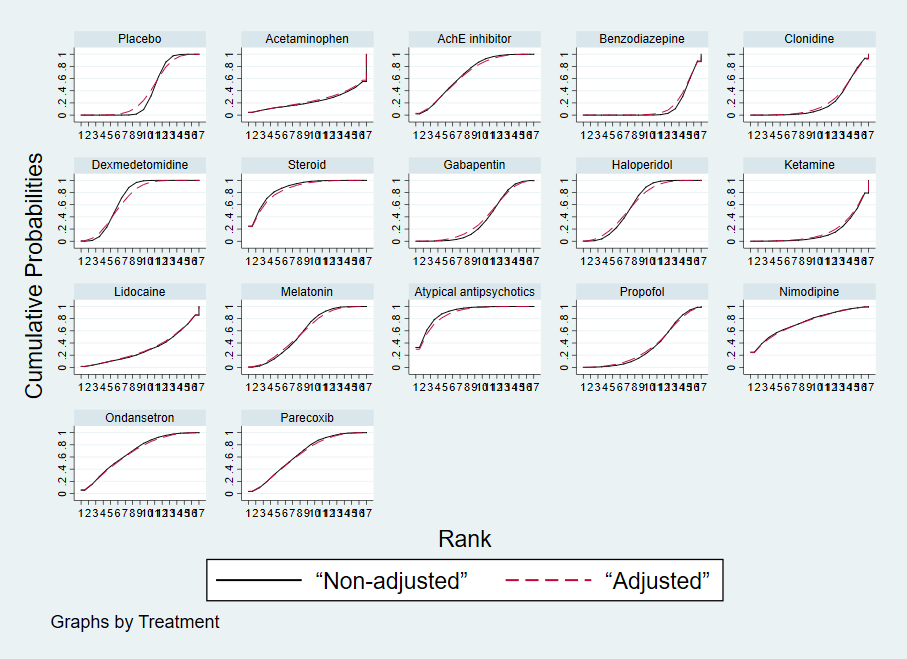


**Supplemental Figure S12**. Relative ranking plot of the incidence of postoperative delirium in the subgroup of patients receiving non-cardiac surgery.


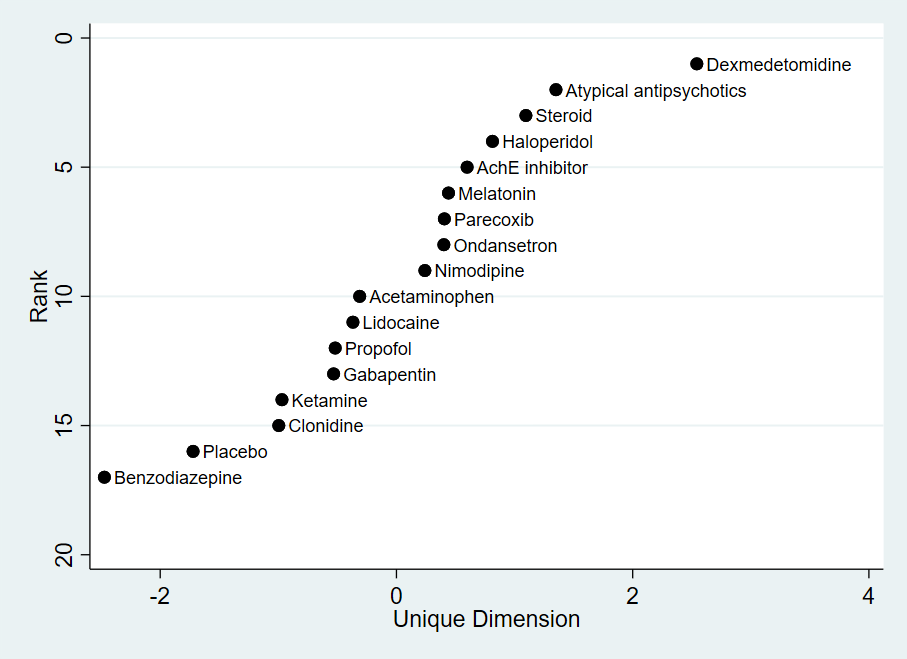


**Supplemental Figure S13**. Network plot of our network of postoperative delirium in the subgroup analysis for studies used CAM or CAM-ICU criteria.
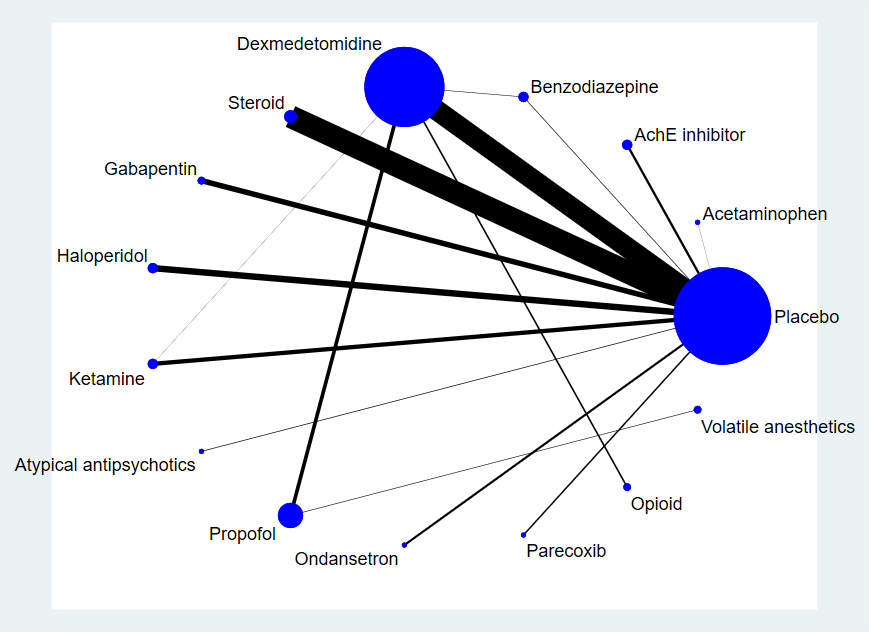


**Supplemental Figure S14**. Cumulative ranking plots of the pharmacologic agents in the subgroup analysis for studies used CAM or CAM-ICU criteria.

**
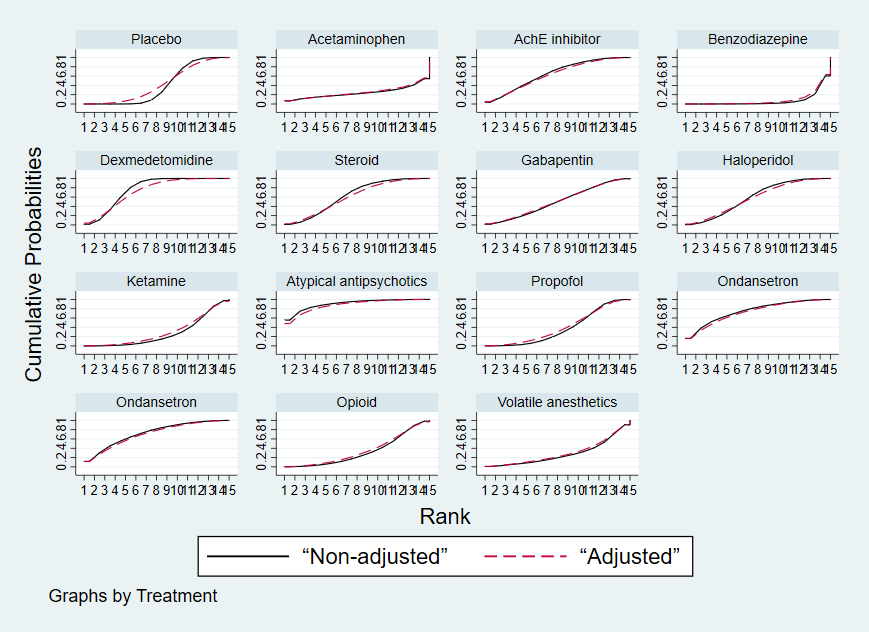
**

**Supplemental Figure S15**. Relative ranking plot of the incidence of postoperative delirium in the subgroup analysis for studies used CAM or CAM-ICU criteria.


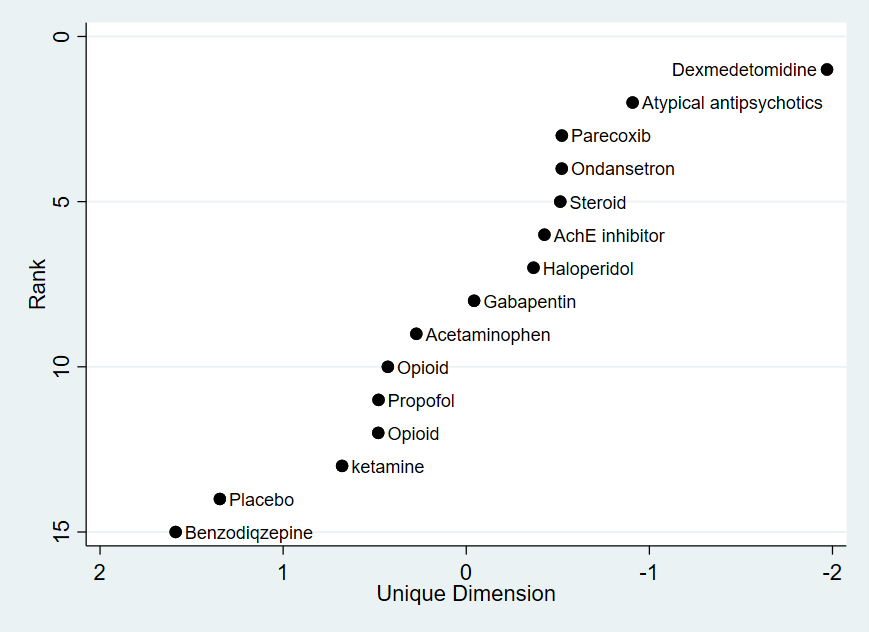


**Supplemental Figure S16**. Network plot of our network of postoperative delirium in the subgroup of patients aged ≥70 years.


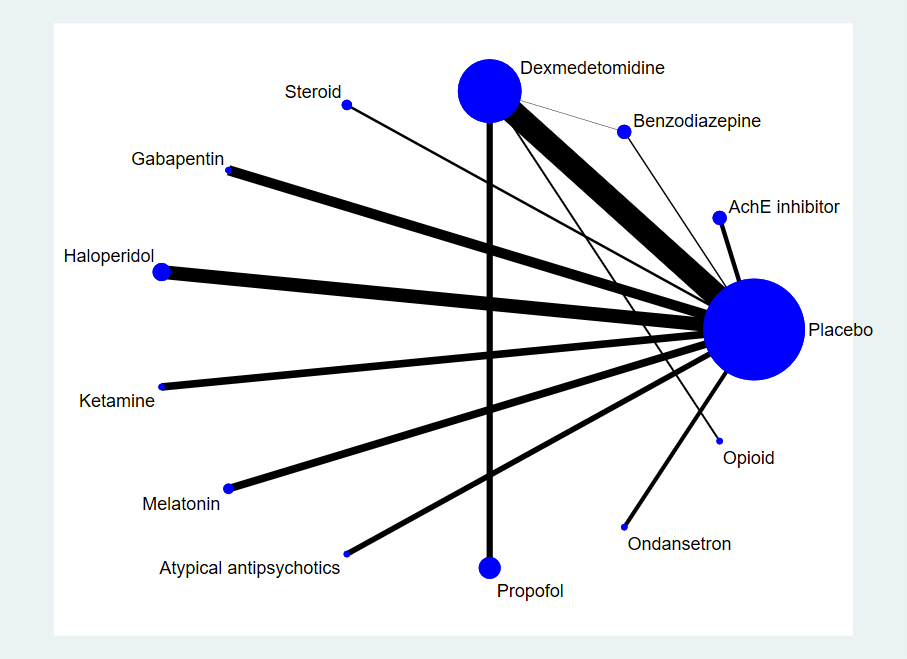


**Supplemental Figure S17**. Cumulative ranking plots of the pharmacologic agents in the subgroup of patients aged ≥70 years.


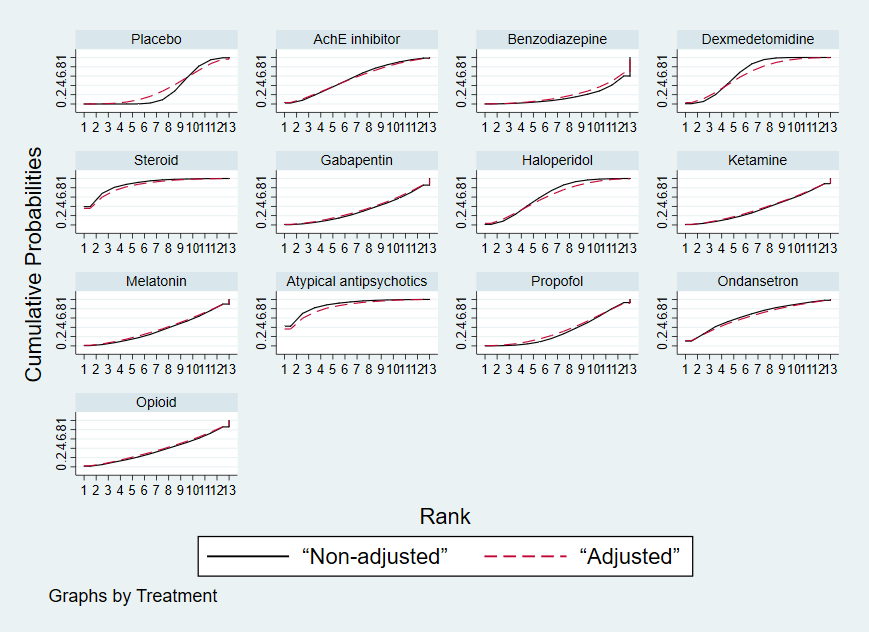


**Supplemental Figure S18**. Relative ranking plot of the incidence of postoperative delirium in the subgroup of patients aged ≥70 years.


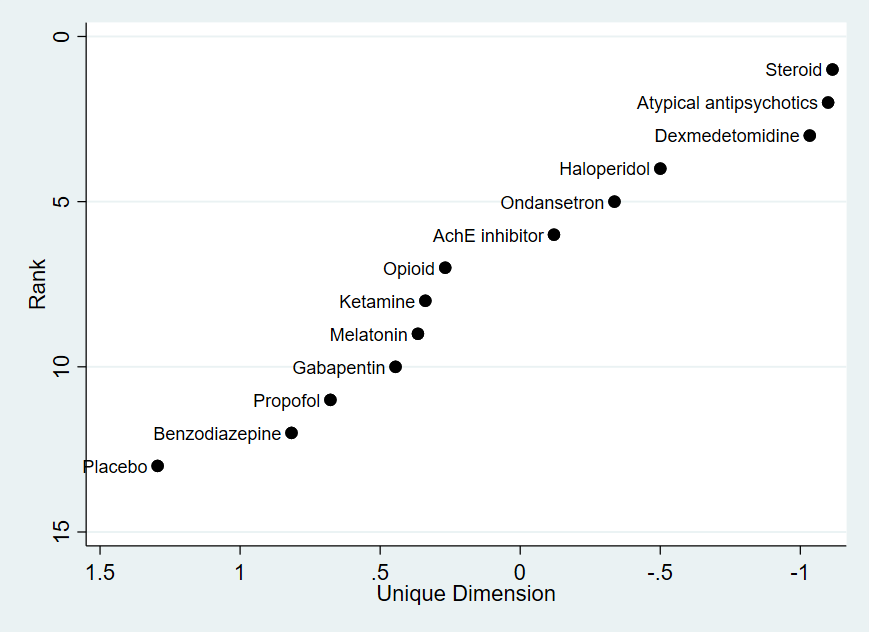


**Supplemental Table S1**. Characteristics of included studies.

| **ID** | | **Study** | | **Type of anesthesia** | | **Type of surgery** | | **Delirium assessment tool** | | **Delirium endpoint** | | **Interventions** | | **No in each arm** | | **M/F** | | **Age (year)** | | **Incidence of delirium, n (%)** | |
| --- | --- | --- | --- | --- | --- | --- | --- | --- | --- | --- | --- | --- | --- | --- | --- | --- | --- | --- | --- | --- | --- |
| 1 | | Greenberg 2018 | | GA | | Craniotomy | | CAM-ICU | | 24h | | Acetaminophen 1g IV upon surgical closure, and q6h for 18h postoperatively | | 66 | | 26/40 | | 59 (13) | | 1 (1.5%) | |
|  |  |  |  |  |  |  |  |  |  |  |  | placebo | | 65 | | 29/36 | | 56 (15) | | 0 (0%) | |
| 2 | | Liptzin 2005 | | NA | | Total knee or hip arthroplasty | | DSM-IV | | 14d | | Donepezil 5mg/d PO, for preoperative 14d and postoperative 14d | | 39 | | 14/26 | | 66.8 (8.9) | | 8 (20.5%) | |
|  |  |  |  |  |  |  |  |  |  |  |  | placebo | | 41 | | 20/21 | | 67.6 (8.6) | | 7 (17.1%) | |
| 3 | | Marcantonio 2011 | | NA | | Surgical repair of hip fracture | | CAM | | 6 weeks | | Donepezil 5mg/d PO for 30d | | 7 | | 2/5 | | 88 (5.2) | | 3 (42.9%) | |
|  |  |  |  |  |  |  |  |  |  |  |  | placebo | | 9 | | 5/4 | | 87 (3.7) | | 4 (44.4%) | |
| 4 | | Sampson 2007 | | NA | | Total hip replacement | | DSI | | 4d | | Donepezil 5mg/d PO preop and postop 3d | | 19 | | 11/8 | | 69.7 (8.4) | | 2 (9.5%) | |
|  |  |  |  |  |  |  |  |  |  |  |  | placebo | | 14 | | 6/8 | | 65.1 (11.1) | | 5 (35.7%) | |
| 5 | | Gamberini 2009 | | GA | | Cardiac surgery with CPB | | CAM | | 6d | | Rivastigmine 1.5mg tid PO | | 56 | | 37/19 | | 74.1 (5.2) | | 18(32%) | |
|  |  |  |  |  |  |  |  |  |  |  |  | placebo | | 57 | | 40/17 | | 74.4 (5.9) | | 17(30%) | |
| 6 | | Youn 2016 | | GA | | Femoral neck/intertrochanter fracture surgery | | CAM | | 7d | | Rivastigmine 4.6 mg patch preop 3d and postop 7d | | 31 | | 12/19 | | 79.4 (6.3) | | 5(16%) | |
|  |  |  |  |  |  |  |  |  |  |  |  | No treatment | | 31 | | 14/17 | | 79.2 (5.8) | | 14(45%) | |
| 7 | | Aizawa 2002 | | GA | | Resection of gastric or colorectal cancer | | DSM-IV | | 7d | | Diazepam 0.1 mg/kg IM, flunitrazepam0.04 mg/kg IV, pethidine 1 mg/kg IV (POD 1-3) | | 20 | | 15/5 | | 75.9 (4.5) | | 1(5%) | |
|  |  |  |  |  |  |  |  |  |  |  |  | No treatment | | 20 | | 11/9 | | 76.2 (4.1) | | 7(35%) | |
| 8 | | Rubino 2010 | | GA | | Aortic dissection surgery | | DSM-IV | | Postoperative period | | clonidine 0.5 mg/kg IV, then 1–2 mg/kg/h during weaning phase | | 15 | | 10/5 | | 63.9(8.9) | | 6(40%) | |
|  |  |  |  |  |  |  |  |  |  |  |  | placebo | | 15 | | 8/7 | | 61.3(6.3) | | 5(33%) | |
| 9 | | Andjelkovic 2019 | | GA | | Laparoscopic intestine resection | | DOS | | 2d | | dexmedetomidine 0.5 mcg/kg/h IV intraop | | 19 | | 9/10 | | 68 (range 42-83) | | 0(0%) | |
|  |  |  |  |  |  |  |  |  |  |  |  | lidocaine 1.5 mg/kg/h IV intraop | | 20 | | 12/8 | | 63.5 (36-79) | | 1(5%) | |
|  |  |  |  |  |  |  |  |  |  |  |  | placebo | | 20 | | 10/10 | | 58 (36-85) | | 1(5%) | |
| **ID** | | **Study** | | **Type of anesthesia** | | **Type of surgery** | | **Delirium assessment tool** | | **Delirium endpoint** | | **Interventions** | | **No in each arm** | | **M/F** | | **Age (year)** | | **Incidence of delirium, n (%)** | |
| 10 | | Azeem 2018 | | GA | | Cardiac surgery | | CAM-ICU | | 7d | | Dexmedetomidine 1 μg/kg IV, then 0.2–0.7 μg/kg/h IV | | 30 | | 13/17 | | 65.3(4.8) | | 1(3%) | |
|  |  |  |  |  |  |  |  |  |  |  |  | Morphine 10–50 μg/kg/h IV, midazolam 0.05–0.2 mg/kg IV | | 30 | | 15/15 | | 66.7(5.6) | | 2(7%) | |
| 11 | | Balkanay 2015 | | GA | | CABG with CPB | | NA | | NA | | Dexmedetomidine 0.04-0.50 μg/kg/h IV | | 60 | | 65/23 | | 60.5(8.6) | | 0(0%) | |
|  |  |  |  |  |  |  |  |  |  |  |  | Placebo | | 28 | |  |  |  |  | 1(4%) | |
| 12 | | Chang 2017 | | GA | | Modified radical mastectomy | | NA | | 24h | | Dexmedetomidine 0.5 μg/kg IV after induction, 6 μg/h after surgery | | 29 | | 0/29 | | 48(10.8) | | 0(0%) | |
|  |  |  |  |  |  |  |  |  |  |  |  | Placebo | | 28 | | 0/28 | | 47(11.5) | | 0(0%) | |
| 13 | | Chang YF 2018 | | GA | | Major abdominal surgery | | CAM-ICU | | 24h | | Dexmedetomidine 0.1–0.7 μg/kg/h IV | | 31 | | 19/12 | | 71(12) | | 0(0%) | |
|  |  |  |  |  |  |  |  |  |  |  |  | Propofol 0.3–1.6 mg/kg/h IV | | 29 | | 16/13 | | 70(10) | | 0(0%) | |
| 14 | | Cheng XQ 2019 | | GA | | Gastro-intestinal laparotomy | | NA | | During PACU stay | | Dexmedetomidine 0.5μg/kg, then 0.4μg/kg/h IV | | 269 | | 194/75 | | 71(65-89) | | 14(5%) | |
|  |  |  |  |  |  |  |  |  |  |  |  | Placebo | | 266 | | 198/68 | | 70(65-85) | | 27(10%) | |
| 15 | | Corbett 2005 | | GA | | CABG | | NA | | NA | | Dexmedetomidine 1μg/kg, then 0.4μg/kg/h IV | | 43 | | 35/8 | | 63.6(10.1) | | 1(2%) | |
|  |  |  |  |  |  |  |  |  |  |  |  | Propofol 5–75  μg/kg/min IV | | 46 | | 38/8 | | 32.4(10.7) | | 1(2%) | |
| 16 | | Deiner 2017 | | GA | | Major noncardiac surgery | | CAM-ICU | | 5d | | Dexmedetomidine 0.5 μg/kg/h IV | | 189 | | 92/97 | | 74(71-78) | | 34(18%) | |
|  |  |  |  |  |  |  |  |  |  |  |  | Placebo | | 201 | | 98/103 | | 74(71-78) | | 35(17%) | |
| 17 | | Djaiani 2016 | | GA | | Cardiac surgery with CPB | | CAM, CAM-ICU | | 5d | | Dexmedetomidine 0.4μg/kg, then 0.2–0.7μg/kg/h IV | | 91 | | 68/23 | | 72.7(6.4) | | 16(18%) | |
|  |  |  |  |  |  |  |  |  |  |  |  | Propofol 25–50μg/kg/min IV | | 92 | | 70/22 | | 72.4(6.2) | | 29(32%) | |
| 18 | | Guo 2015 | | GA | | Oral cancer radical surgery | | CAM-ICU | | 3d | | Dexmedetomidine 0.2μg/kg/h IV | | 78 | | 41/37 | | 71.9(5.1) | | 6(8%) | |
|  |  |  |  |  |  |  |  |  |  |  |  | Placebo | | 78 | | 39/39 | | 70.7(4.6) | | 21(27%) | |
| 19 | | Gupta 2011 | | ketamine anesthesia | | Elective surgery | | NA | | 1d (during PACU stay) | | dexmedetomidine 1 μg/kg IV | | 40 | | 15/25 | | 32.8(8.5) | | 0(0%) | |
|  |  |  |  |  |  |  |  |  |  |  |  | Midazolam 0.02 mg/kg IV | | 40 | | 11/29 | | 30.8(9.2) | | 0(0%) | |

| **ID** | | **Study** | | **Type of anesthesia** | | **Type of surgery** | | **Delirium assessment tool** | | **Delirium endpoint** | | **Interventions** | | **No in each arm** | | **M/F** | | **Age (year)** | | **Incidence of delirium, n (%)** | |
| --- | --- | --- | --- | --- | --- | --- | --- | --- | --- | --- | --- | --- | --- | --- | --- | --- | --- | --- | --- | --- | --- |
| 20 | | He 2018 | | GA | | Surgery of vertebral fracture | | CAM | | 1d | | Dexmedetomidine 0.5 μg/kg IV, then 0.4 μg/kg/h IV | | 30 | | 16/14 | | 82.5 (5.4) | | 2 (7%) | |
|  |  |  |  |  |  |  |  |  |  |  |  | Midazolam 0.03 mg/kg IV | | 30 | | 19/11 | | 81.9 (6.2) | | 14 (47%) | |
|  |  |  |  |  |  |  |  |  |  |  |  | Placebo | | 30 | | 17/13 | | 83.2 (5.1) | | 6 (20%) | |
| 21 | | Huang 2014 | | GA | | Elective major surgery | | CAM-ICU | | NA | | Dexmedetomidine 0.2-0.7 μg/kg/h | | 54 | | 32/22 | | 73.7 (3.1) | | 2 (4%) | |
|  |  |  |  |  |  |  |  |  |  |  |  | Propofol 0.3-0.4 mg/kg/h | | 54 | | 30/24 | | 74.2 (4.2) | | 5 (9%) | |
| 22 | | Huyan 2019 | | GA | | Radical pulmonary resection | | ICDSC | | 1d | | Dexmedetomidine 0.5 μg/kg IV, then 0.1 μg/kg/h IV | | 173 | | 89/84 | | 70 (5) | | 27 (16%) | |
|  |  |  |  |  |  |  |  |  |  |  |  | Placebo | | 173 | | 94/79 | | 71 (6) | | 46 (27%) | |
| 23 | | Kang 2018 | | GA | | Valve replacement surgery | | Neurology physician’s diagnosis | | 3d | | Dexmedetomidine 0.6 μg/kg, then 0.2 μg/kg/h IV | | 50 | | 16/34 | | 54.9 (8.6) | | 0 (0%) | |
|  |  |  |  |  |  |  |  |  |  |  |  | Control (no treatment) | | 47 | | 18/29 | | 56.5 (6.9) | | 1 (2%) | |
| 24 | | Kim 2019 | | GA | | VATS lobectomy/segmentectomy | | CAM, CAM-ICU | | 3d | | Dexmedetomidine 0.5 μg/kg/h IV | | 60 | | 28/32 | | 63 (58-68) | | 15 (25%) | |
|  |  |  |  |  |  |  |  |  |  |  |  | Placebo | | 60 | | 30/30 | | 59 (56-65) | | 15 (25%) | |
| 25 | | Lee 2018 | | GA | | laparoscopic major non-cardiac surgery | | CAM | | 5d | | Dexmedetomidine 1 μg/kg IV and/or 0.2–0.7 μg/kg/h IV | | 209 | | 94/115 | | 72.7 (6.0) | | 30 (14%) | |
|  |  |  |  |  |  |  |  |  |  |  |  | Placebo | | 109 | | 47/62 | | 73.8 (6.1) | | 27 (25%) | |
| 26 | | Li 2017 | | GA | | Cardiac surgery | | CAM, CAM-ICU | | 5d | | Dexmedetomidine 0.6 μg/kg, then 0.4 μg/kg/h (intraop), 0.1 μg/kg/h IV (postop) | | 142 | | 95/47 | | 66.4 (5.4) | | 7 (5%) | |
|  |  |  |  |  |  |  |  |  |  |  |  | Placebo | | 143 | | 102/41 | | 67.5 (5.3) | | 11 (8%) | |
| 27 | | Liu X 2016 | | GA | | Cardiac valve  surgery with CPB | | CAM-ICU | | During ICU stay | | Dexmedetomidine 0.2-1.5 μg/kg/h IV | | 29 | | 10/19 | | 53 (48-63) | | 0 (0%) | |
|  |  |  |  |  |  |  |  |  |  |  |  | Propofol 5-50 μg/kg/min IV | | 32 | | 15/17 | | 55 (48-62) | | 2 (6%) | |
| 28 | | Liu Y 2016 | | GA | | Total hip joint/knee joint/shoulder joint replacement  surgery | | CAM | | 7d | | Dexmedetomidine 0.2-0.4µg/kg/h IV | | 99 | | 44/55 | | 71.9 (8.1) | | 15 (15%) | |
|  |  |  |  |  |  |  |  |  |  |  |  | Placebo | | 98 | | 52/46 | | 73.8 (8.7) | | 43 (44%) | |
| 29 | | Ma 2013 | | GA | | Orthopedic surgery | | CAM | | 3d | | Dexmedetomidine 1 μg/kg, 0.5 μg/kg/h IV | | 30 | | 10/20 | | 69.1 (7.0) | | 2 (7%) | |
|  |  |  |  |  |  |  |  |  |  |  |  | Ketamine 0.5 mg/kg IV | | 30 | | 16/14 | | 66.2 (8.1) | | 8 (27%) | |
|  |  |  |  |  |  |  |  |  |  |  |  | Placebo | | 30 | | 18/12 | | 67.6 (7.5) | | 3 (10%) | |
| **ID** | | **Study** | | **Type of anesthesia** | | **Type of surgery** | | **Delirium assessment tool** | | **Delirium endpoint** | | **Interventions** | | **No in each arm** | | **M/F** | | **Age (year)** | | **Incidence of delirium, n (%)** | |
| 30 | | Maldonado 2009 | | GA | | Cardiac valve operation | | DSM-IV-TR | | 3d | | Dexmedetomidine 0.4 μg/kg, then 0.2-0.7 μg/kg/h IV | | 40 | | 26/14 | | 55 (16) | | 4 (10%) | |
|  |  |  |  |  |  |  |  |  |  |  |  | Midazolam 0.5-2 mg/h IV | | 40 | | 27/13 | | 60 (16) | | 17 (43%) | |
|  |  |  |  |  |  |  |  |  |  |  |  | Propofol 25-50 μg/kg/min IV | | 36 | | 22/16 | | 58 (18) | | 16 (44%) | |
| 31 | | Massoumi 2019 | | GA | | CABG | | CAM-ICU | | 3d | | Dexmedetomidine 1 μg/kg, 0.2-0.7 μg/kg/h IV | | 44 | | 72/16 | | 61.8 (7.9) | | 4 (9%) | |
|  |  |  |  |  |  |  |  |  |  |  |  | Placebo | | 44 | |  |  | 61.3 (8.9) | | 9 (20%) | |
| 32 | | Mei 2018 | | Nerve block | | Total hip arthroplasty | | CAM | | 3d | | Dexmedetomidine 0.8-1.0 μg/kg, then 0.1-0.5 μg/kg/h IV | | 148 | | 64/84 | | 76 (7) | | 11 (7%) | |
|  |  |  |  |  |  |  |  |  |  |  |  | Propofol target-controlled infusion with effect site concentration 0.8-1.0 μg/mL | | 148 | | 71/77 | | 74 (6) | | 24 (16%) | |
| 33 | | Mishina 2018 | | LA | | Hernia surgery | | NA | | 1d | | Dexmedetomidine 3 μg/kg/h for 10 min, then 0.4 μg/kg/h IV | | 99 | | 98/1 | | 66.5 (11.7) | | 1 (1%) | |
|  |  |  |  |  |  |  |  |  |  |  |  | Midazolam 2 mg IV | | 97 | | 91/6 | | 65.2 (11.6) | | 2 (2%) | |
| 34 | | Naik 2016 | | GA | | Thoracic, lumbar spine surgery | | CAM-ICU | | 1d | | Dexmedetomidine 1 μg/kg, then 0.5 μg/kg/h IV | | 63 | | NA | | 64 (57-69.5) | | 1 (2%) | |
|  |  |  |  |  |  |  |  |  |  |  |  | Placebo | | 68 | | NA | | 65 (56-70) | | 3 (4%) | |
| 35 | | Susheela 2017 | | GA | | Cardiac surgery | | CAM | | NA | | Dexmedetomidine 0.1–1.0 μg/kg/hr IV | | 3 | | NA | | NA | | 2 (67%) | |
|  |  |  |  |  |  |  |  |  |  |  |  | Propofol 25–100 μg/kg/min IV infusion | | 3 | | NA | | NA | | 2 (67%) | |
| 36 | | Park 2014 | | GA | | Cardiac surgery | | CAM-ICU | | 3d | | Dexmedetomidine 0.5 μg/kg loading; then 0.2-0.8 μg/kg/hr IV | | 67 | | 39/28 | | 51.1 (16.1) | | 6 (9%) | |
|  |  |  |  |  |  |  |  |  |  |  |  | Remifentanil 1000-2500 μg/hr IV | | 75 | | 40/35 | | 54.4 (14.0) | | 17 (23%) | |
| 37 | | Priye 2015 | | GA | | Cardiac surgery | | RASS | | Postoperative period | | Dexmedetomidine 0.4 μg/kg/h IV for 12 h | | 32 | | 17/15 | | 45.1 (14.7) | | 1 (3%) | |
|  |  |  |  |  |  |  |  |  |  |  |  | Placebo | | 32 | | 16/16 | | 41.4 (11.9) | | 5 (16%) | |

| **ID** | **Study** | **Type of anesthesia** | **Type of surgery** | **Delirium assessment tool** | **Delirium endpoint** | **Interventions** | **No in each arm** | **M/F** | **Age (year)** | **Incidence of delirium, n (%)** |
| --- | --- | --- | --- | --- | --- | --- | --- | --- | --- | --- |
| 38 | Shehabi 2009 | GA | Cardiac surgery | CAM-ICU | 5d | Dexmedetomidine 0.1-0.7 μg/kg/h IV | 152 | 114/38 | 71.5 (66-76) | 13 (9%) |
|  |  |  |  |  |  | Morphine 10-70 μg/kg/h IV | 147 | 111/36 | 71.0 (65-75) | 22 (15%) |
| 39 | Sheikh 2018 | GA | Cardiac surgery | Clinical features | Postoperative period | Dexmedetomidine 1 μg/kg IV loading; then 0.2–0.6 μg/kg/h IV infusion | 30 | NA | 33.6 (11.8) | 1 (3%) |
|  |  |  |  |  |  | Propofol 0.25–1 mg/kg/h IV infusion | 30 | NA | 33.6 (9.5) | 7 (23%) |
| 40 | Shi 2019 | GA | Cardiac surgery | CAM | 5d | Dexmedetomidine 0.4–0.6 mg/kg/h IV | 84 | 63/21 | 74.7 (7.2) | 33 (39%) |
|  |  |  |  |  |  | Without dexmedetomidine | 80 | 56/24 | 74.2 (7.7) | 21 (26%) |
| 41 | Shu 2017 | GA | Cardiac valve replacement surgery | DRS | 7d | Dexmedetomidine 1.0 μg/kg, then 0.5 μg/kg/h IV | 30 | 18/12 | 47.7 (8.7) | 4 (13%) |
|  |  |  |  |  |  | Placebo | 30 | 16/14 | 46.8 (7.4) | 7 (23%) |
| 42 | Su 2016 | GA or Epidural+GA | Noncardiac surgery | CAM-ICU | 7d | Dexmedetomidine 0.1 μg/kg/h IV | 350 | NA | NA | 32 (9%) |
|  |  |  |  |  |  | Placebo | 350 | NA | NA | 79 (23%) |
| 43 | Sun 2019 | GA | Major elective noncardiac surgery | CAM, CAM-ICU | 5d | Dexmedetomidine 0.1 μg/kg/h IV | 281 | 161/120 | 68 (66-73) | 33 (12%) |
|  |  |  |  |  |  | Placebo | 276 | 154/122 | 69 (65-74) | 38 (14%) |
| 44 | Tang 2018 | GA | Embolization of intracranial  aneurysms | Modified CAM-S | 24h | Dexmedetomidine 1.0 μg/kg, then 0.3 μg/kg/h IV | 54 | 30/24 | 62 (7.3) | 8 (15%) |
|  |  |  |  |  |  | Placebo | 52 | 26/26 | 61.1 (8.5) | 12 (23%) |
| 45 | Wan 2011 | GA | Abdominal, thoracic, spine, lower limb surgery | NA | NA | Dexmedetomidine 3.06 μg/kg IV for 10 hr | 102 | NA | NA | 4 (4%) |
|  |  |  |  |  |  | Midazolam 0.34 mg/kg IV for 11 hr | 98 | NA | NA | 31 (32%) |
| 46 | Wang 2015 | GA | Spine surgery | CAM | 3d | Dexmedetomidine 3 μg/kg and sufentanil 2 μg/kg via IV PCA | 75 | 39/36 | 67.2 (5.6) | 3 (4%) |
|  |  |  |  |  |  | Sufentanil 2 μg/kg via IV PCA | 77 | 38/39 | 68.3 (5.2) | 8 (10%) |

| **ID** | **Study** | **Type of anesthesia** | **Type of surgery** | **Delirium assessment tool** | **Delirium endpoint** | **Interventions** | **No in each arm** | **M/F** | **Age (year)** | **Incidence of delirium, n (%)** |
| --- | --- | --- | --- | --- | --- | --- | --- | --- | --- | --- |
| 47 | Wu 2016 | GA or GA+Regional | Noncardiac surgery | CAM | 7d | Dexmedetomidine 0.1 μg/kg/h IV for 15h | 38 | 24/14 | 76 (6) | 2 (5%) |
|  |  |  |  |  |  | Placebo | 38 | 20/18 | 74 (5) | 3 (8%) |
| 48 | Xie 2018 | GA | Hip surgery | CAM | 4d | Dexmedetomidine 0.5 μg/kg, then 0.4 μg/kg/h IV | 70 | 38/32 | 68.2 (5.6) | 3 (4%) |
|  |  |  |  |  |  | Placebo | 70 | 36/34 | 69.7 (5.3) | 12 (17%) |
| 49 | Xuan 2018 | GA | Joint replacement surgery | CAM, CAM-ICU | 7d | Dexmedetomidine 0.1 μg/kg/h | 227 | 96/131 | 66.6 (7.5) | 30 (13%) |
|  |  |  |  |  |  | Placebo | 226 | 101/125 | 66.9 (5.1) | 64 (28%) |
| 50 | Yang 2015 | GA | Maxillofacial surgery with microvascular free flap reconstruction | CAM-ICU | 5d | Dexmedetomidine 0.5 μg/kg/h for 1 h, then 0.2-0.7 μg/kg/h IV | 39 | 21/18 | 50.3 (15) | 2 (5%) |
|  |  |  |  |  |  | Placebo | 40 | 21/19 | 50.6 (12.3) | 5 (12.5%) |
| 51 | Lee 2020 | GA | Liver transplantation | CAM-ICU | Postoperative period | Dexmedetomidine 0.1 μg/kg/h IV | 100 | 71/29 | 56 (52-61) | 9 (9%) |
|  |  |  |  |  |  | Placebo | 101 | 73/28 | 55 (50-62) | 6 (5.9%) |
| 52 | Yu 2017 | GA | Thoracic surgery | CAM | 3d | Dexmedetomidine 0.2-0.7 μg/kg/h IV | 46 | 25/21 | 69 (4) | 3 (6.5%) |
|  |  |  |  |  |  | Midazolam Maleate 0.05 μg/kg/h, then 0.02-0.08 μg/kg/h IV | 46 | 26/20 | 69 (5) | 10 (22%) |
| 53 | Dighe 2014 | SA | Total knee arthroplasty | Validated medical chart abstraction tool | NA | Gabapentin 600 mg PO preop, gabapentin 200 mg tid for 4 days postop | 83 | 39/44 | 62.5 (6.5) | 10 (12%) |
|  |  |  |  |  |  | Placebo | 78 | 41/37 | 62.9 (7.2) | 7 (9%) |
| 54 | Leung 2006 | GA | Spine surgery | CAM | 3d | Gabapentin 900 mg PO preop, and 900 mg qd PO for 3 days | 9 | 4/5 | 57.2 (10.3) | 0 (0%) |
|  |  |  |  |  |  | Placebo | 12 | 7/5 | 61.4 (11.3) | 5 (42%) |
| 55 | Leung 2017 | GA or neuraxial | Spine surgery or hip or knee arthroplasty | CAM | 3d | Gabapentin 900 mg PO preop, and 300 mg tid PO for 3 days | 350 | 157/193 | 73 (6) | 78 (22%) |
|  |  |  |  |  |  | Placebo | 347 | 189/158 | 73 (6) | 73 (21%) |

| **ID** | **Study** | **Type of anesthesia** | **Type of surgery** | **Delirium assessment tool** | **Delirium endpoint** | **Interventions** | **No in each arm** | **M/F** | **Age (year)** | **Incidence of delirium, n (%)** |
| --- | --- | --- | --- | --- | --- | --- | --- | --- | --- | --- |
| 56 | Fukata 2016 | GA or SA | Abdominal surgery or orthopedic surgery | NEECHAM confusion scale | 10d | Haloperidol 5mg qd IV for postoperative 5 days | 101 | 50/51 | 82 (4.4) | 18 (18%) |
|  |  |  |  |  |  | Placebo | 100 | 51/49 | 81.3 (4.3) | 32 (32%) |
| 57 | Kalisvaart 2005 | NA | Hip surgery | CAM, DSM-IV | Postoperative period | Haloperidol 0.5mg tid PO for postoperative 3 days | 212 | 40/172 | 78.7 (6) | 32 (15%) |
|  |  |  |  |  |  | Placebo | 218 | 47/171 | 79.8 (6) | 36 (17%) |
| 58 | Kaneko 1999 | GA | Gastrointestinal surgery | DSM-III-R | 5d | Haloperidol 5mg qd IV for postoperative 5 days | 38 | 24/38 | 72.4 (8.2) | 4 (11%) |
|  |  |  |  |  |  | Placebo | 40 | 26/40 | 73.1 (9.3) | 13 (33%) |
| 59 | Khan 2018 | GA | Thoracic surgery | CAM-ICU | Postoperative period | Haloperidol 0.5mg tid IV for a total of 11 doses | 68 | 46/22 | 60 (52-68) | 15 (22%) |
|  |  |  |  |  |  | Placebo | 67 | 54/13 | 62.3 (53-69) | 19 (28%) |
| 60 | Wang 2012 | GA or GA+Regional | Noncardiac surgery | CAM-ICU | 7d | Haloperidol 0.5mg IV, then 0.1 mg/h IV for 12 hrs | 229 | 145/84 | 74 (5.8) | 35 (15%) |
|  |  |  |  |  |  | Placebo | 228 | 143/85 | 74.4 (7) | 53 (23%) |
| 61 | Avidan 2017 | GA or GA+Regional | Major cardiac or noncardiac surgery | CAM, CAM-ICU | 3d | Ketamine 0.5-1.0 mg/kg IV after induction | 437 | NA | 70 (7) | 85 (19%) |
|  |  |  |  |  |  | Placebo | 217 | 135/87 | 70 (7) | 43 (20%) |
| 62 | Du 2011 | GA | Laparoscopic surgery | NA | 1h | Ketamine 0.25 mg/kg IV before induction | 20 | 0/20 | 35.4 (7.9) | 0 (0%) |
|  |  |  |  |  |  | Placebo | 20 | 0/20 | 39.1 (11.5 | 0 (0%) |
| 63 | Gecaj-Gashi 2010 | GA | Surgery | NA | NA | Ketamine 0.5 mg/kg IV 20min before completion of surgery | 33 | NA | NA | 0 (0%) |
|  |  |  |  |  |  | Placebo | 33 | NA | NA | 0 (0%) |
| 64 | Hudetz 2009 | GA | Cardiac surgery | ICDSC | 5d | Ketamine 0.5 mg/kg IV | 29 | 29/0 | 68 (8) | 1 (3.4%) |
|  |  |  |  |  |  | Placebo | 29 | 29/0 | 60 (8) | 9 (31%) |
| 65 | Dewinter 2017 | GA | Posterior spinal arthrodesis | NA | NA | Lidocaine 1.5 mg/kg IV, then 1.5 mg/kg/h IV for 6h | 35 | 14/21 | 49 (15-56) | 1 (3%) |
|  |  |  |  |  |  | Placebo | 34 | 8/26 | 46 (16-57) | 0 (0%) |
| 66 | de Jonghe 2014 | GA or neuraxial | Surgical treatment of hip fracture | DSM-IV | 7d | Melatonin 3mg qd PO for 5 d | 186 | 53/133 | 84.1 (8) | 55 (30%) |
|  |  |  |  |  |  | Placebo | 192 | 62/130 | 83.4 (7.5) | 49 (25.5%) |

| **ID** | **Study** | **Type of anesthesia** | **Type of surgery** | **Delirium assessment tool** | **Delirium endpoint** | **Interventions** | **No in each arm** | **M/F** | **Age (year)** | **Incidence of delirium, n (%)** |
| --- | --- | --- | --- | --- | --- | --- | --- | --- | --- | --- |
| 67 | Yamaguchi 2014 | NA | Total knee arthroplasty | ICDSC | 4d | Ramelteon 8 mg/day PO for 4d | 21 | NA | ≥ 70 | 0 (0%) |
|  |  |  |  |  |  | Placebo | 21 | NA | ≥ 70 | 2 (9.5%) |
| 68 | Clemmesen 2018 | GA or neuraxial | Hip fracture surgery | CAM-S | 3d | Methylprednisolone 125 mg IV (preop) | 59 | 22/37 | 79 (8) | 10 (17%) |
|  |  |  |  |  |  | Placebo | 58 | 20/38 | 81 (9) | 19 (33%) |
| 69 | Whitlock 2015 | GA | Cardiac surgery | CAM | 3d | Methylprednisolone 250 mg IV at induction, 250 mg at  CPB initiation | 3755 | 2257/1498 | 67.5 (13.6) | 295 (7.9%) |
|  |  |  |  |  |  | Placebo | 3752 | 2280/1472 | 67.3 (13.8) | 289 (7.7%) |
| 70 | Li YN 2017 | GA | Spine surgery | Nu-DESC | 7d | Nimodipine 7.5 μg/kg/h for 30min before induction | 30 | 11/19 | 69 (4) | 2 (7%) |
|  |  |  |  |  |  | Placebo | 30 | 13/17 | 70 (4) | 5 (17%) |
| 71 | Larsen 2010 | GA or GA plus regional | Joint replacement surgery | DSM-III-R | 4d | Olanzapine 5mg IV (immediately before surgery) | 196 | 102/94 | 73.4 (6.1) | 28 (14%) |
|  |  |  |  |  |  | Placebo | 204 | 81/123 | 74.0 (6.2) | 82 (40%) |
| 72 | Papadopoulos 2014 | GA | Femoral or hip fracture rehabilitation surgery | CAM | 2d | Ondansetron 8mg/d IV for 5d | 51 | NA | 71.9 (12.5) | 18 (35%) |
|  |  |  |  |  |  | Placebo | 55 | NA | 70.7 (13.3) | 29 (53%) |
| 73 | Mu 2017 | Combined spinal-epidural | Total hip or knee  replacement surgery | CAM | 5d | Parecoxib 40mg IV q12h for 72h | 310 | 81/229 | 69.6 (6.5) | 19 (6%) |
|  |  |  |  |  |  | Placebo | 310 | 83/227 | 70.5 (6.9) | 34 (11%) |
| 74 | Farlinger 2018 | SA | Total hip arthroplasty | Validated medical chart abstraction tool | Postoperative period | Pregabalin 150mg PO preoperatively and 75mg bid postoperatively for 7 days | 84 | 43/41 | 60.1 (9.3) | 1 (1.2%) |
|  |  |  |  |  |  | Placebo | 79 | 40/39 | 60.0 (8.9) | 0 (0%) |
| 75 | Royse 2011 | GA | Coronary artery bypass surgery | CAM | 1d | Propofol target concentration infusion 1.5-3 μg/ml | 89 | 80/9 | 63.8 (10.6) | 7 (8%) |
|  |  |  |  |  |  | Desflurane for maintenance of anesthesia | 91 | 73/18 | 61.8 (10.4) | 12 (13%) |
| 76 | Tanaka 2017 | GA | Total knee arthroplasty | CAM | 48h | Propofol for maintenance of anesthesia | 45 | 15/30 | 71 (69-72) | 1 (2%) |
|  |  |  |  |  |  | Desflurane for maintenance of anesthesia | 45 | 25/20 | 70 (69-71) | 0 (0%) |

| **ID** | **Study** | **Type of anesthesia** | **Type of surgery** | **Delirium assessment tool** | **Delirium endpoint** | **Interventions** | **No in each arm** | **M/F** | **Age (year)** | **Incidence of delirium, n (%)** |
| --- | --- | --- | --- | --- | --- | --- | --- | --- | --- | --- |
| 77 | Moscarelli 2018 | GA | Minimally invasive mitral valve repair | NA | Postoperative period | Propofol 2mg/kg IV for induction, 0.1-0.5 mg/kg/min for maintence | 31 | 15/16 | 68.3 (9.5) | 1 (3%) |
|  |  |  |  |  |  | Sevoflurane for maintenance of anesthesia | 31 | 13/18 | 60.7 (12.5) | 0 (0%) |
| 78 | Hakim 2012 | GA | Cardiac surgery | ICDSC | Until discharge | Risperidone 0.5mg PO q 12h, max dose 4 mg/day | 51 | 33/18 | ≥ 65 | 7 (14%) |
|  |  |  |  |  |  | Placebo | 50 | 36/14 | ≥ 65 | 17 (34%) |
| 79 | Prakanrattana 2007 | GA | Cardiac surgery | CAM-ICU | Postoperative period | Risperidone 1mg PO | 63 | 36/27 | 61.3 (9.7) | 7 (11%) |
|  |  |  |  |  |  | Placebo | 63 | 38/25 | 60.7 (9.8) | 20 (32%) |
| 80 | Subramaniam 2019 | GA | Cardiac surgery | CAM, CAM-ICU | During hospital stay | Dexmedetomidine 0.5-1 μg/kg IV, then 0.1-1.4 μg/kg/h IV | 30 | 25/5 | 69 (63-74) | 8 (27%) |
|  |  |  |  |  |  | Propofol 20-100 μg/kg/min IV | 30 | 26/4 | 71 (64-79) | 9 (30%) |
| 81 | Dieleman 2012 | GA | Cardiac surgery | Need for treatment with neuroleptic drugs | Postoperative period | Dexamethasone 1mg/kg IV | 2235 | 1622/613 | 66.2 (11) | 205 (9%) |
|  |  |  |  |  |  | Placebo | 2247 | 1628/619 | 66.1 (10.7) | 262 (12%) |
| 82 | Mardani 2012 | GA | Cardiac surgery | DSM‐IV | 1d(out of 3d) | Dexamethasone 8mg IV before surgery, then 8mg IV q8h for 3d | 43 | 36/7 | 64.55 (11.1) | 4 (9.3%) |
|  |  |  |  |  |  | Placebo | 50 | 44/6 | 60.04 (12.77) | 13 (26%) |
| 83 | Sakic 2015 | SA | Femur fracture surgery | CAM | NA | Dexamethasone 8mg intrathecally | 17 | NA | 83 (73-95) | 3 (18%) |
|  |  |  |  |  |  | No treatment | 11 | NA | 78 (54-91) | 8 (73%) |
| 84 | Sauer 2014 | GA | Cardiac surgery | CAM-ICU | 4d | Dexamethasone 1mg/kg IV | 367 | 255/112 | 67 (12) | 52 (14%) |
|  |  |  |  |  |  | Placebo | 370 | 225/145 | 66 (12) | 55 (15%) |
| 85 | Nickkholgh 2011 | GA | Liver resection | NA | 7d | Melatonin 50 mg/kg through gastric tube | 25 | 17/8 | 59 (10) | 0 (0%) |
|  |  |  |  |  |  | Placebo (microcrystalline cellulose) | 23 | 11/12 | 56 (11) | 1 (4%) |
| 86 | Sultan 2010 | SA | Hip arthroplasty | AMT(abbreviated mental test | 3d | Midazolam 7.5mg PO | 50 | 26/24 | 69.9 (8.2) | 22 (44%) |
|  |  |  |  |  |  | Clonidine 100μg PO | 51 | 27/24 | 71.5 (6.8) | 19 (37%) |
|  |  |  |  |  |  | Melatonin 5mg PO | 53 | 24/29 | 70.4 (7.1) | 5 (9%) |
|  |  |  |  |  |  | No treatment | 49 | 22/27 | 72.3 (6.4) | 16 (33%) |

GA: General Anesthesia; SA: Spinal Anesthesia; LA: Local Anesthesia; DSM: Diagnostic and Statistical Manual of Mental Disorders; DSI: Delirium Symptom Interview; DOS: Delirium Observation Screening; NEECHAM confusion scale: Neelon and Champagne confusion scale; CABG: coronary artery bypass graft; CPB: Cardiopulmonary bypass; ICU: Intensive Care Unit; PACU: Post-Anesthesia Care Unit; RASS: Richmond Agitation and Sedation Scale; CAM: Confusion Assessment Method; CAM-ICU: CAM for Intensive care unit; ICDSC: Intensive care delirium screening checklist; DRS: Delirium Rating Scale; Nursing delirium screening score (Nu-DESC); NA: Not available.

**Supplemental Table S2**. Quality of evidence evaluated by GRADE approach for the incidence of delirium.

GRADE summary of findings table with quality of evidence and absolute anticipated benefits for all agents over placebo.

Using GRADE to rate quality of evidence from a network meta-analysis involved several steps.

(1) We rated quality of evidence for direct comparisons.

(2) We rated quality of evidence for indirect estimates (starting at the lowest rating of the two pairwise direct estimates that contribute as first-order loops to the indirect estimate, which can be rated down further for imprecision or intransitivity). Intransitivity means any dissimilarity between studies in terms of clinical or methodological characteristics.

(3) We rated quality of evidence for the network combining direct and indirect estimates. In this step, if direct and indirect estimates from second-order comparisons are similar, the higher of the ratings was assigned to the network meta-analysis estimates.

Ratings High quality (⊕⊕⊕⊕)—We are very confident that the true effect lies close to that of the estimate of the effect.

Moderate quality (⊕⊕⊕O)—We are moderately confident in the effect estimate: the true effect is likely to be close to the estimate of the effect, but there is a possibility that it is substantially different.

Low quality (⊕⊕OO)—Our confidence in the effect estimate is limited: the true effect may be substantially different from the estimate of the effect.

Very low quality (⊕OOO)—We have very little confidence in the effect estimate: the true effect is likely to be substantially different from the estimate of effect.

|  | Direct evidence | | Indirect evidence | | Network evidence | |
| --- | --- | --- | --- | --- | --- | --- |
| Comparison | OR (95% CI) | GRADE | OR (95% CI) | GRADE | OR (95% CI) | GRADE |
| Acetaminophen vs. control | 2.96 (0.12-71.2) | Low quality (⊕⊕OO)^1.3^ | 3.00 (0.09-97.91) | Low quality (⊕⊕OO)^1.3^ | 3.00 (0.11-82.8) | Low quality (⊕⊕OO)^1.3^ |
| Acetylcholinesterase inhibitor vs. control | 0.78 (0.54-1.12) | Moderate quality (⊕⊕⊕O)^3^ | 0.67 (0.23-1.95) | Low quality (⊕⊕OO)^1.3^ | 0.67 (0.34-1.30) | Moderate quality (⊕⊕⊕O)^3^ |
| Benzodiazepine vs. control | 1.26 (0.84-1.89) | Low quality (⊕⊕OO)^1.3^ | 2.21 (0.82-5.95) | Low quality (⊕⊕OO)^1.3^ | 2.21 (1.28-3.81) | Low quality (⊕⊕OO)^1.3^ |
| Clonidine vs. control | 0.14 (0.02-1.06) | Moderate quality (⊕⊕⊕O)^2^ | 1.64 (0.49-5.44) | Low quality (⊕⊕OO)^1.2^ | 1.64 (0.70-3.86) | Low quality (⊕⊕OO)^1.2^ |
| Dexmedetomidine vs. control | 0.59 (0.52-0.68) | Moderate quality (⊕⊕⊕O)^3^ | 0.51 (0.22-1.21) | Moderate quality (⊕⊕⊕O)^3^ | 0.51 (0.40-0.66) | Moderate quality (⊕⊕⊕O)^3^ |
| Steroid vs. control | 0.88 (0.79-0.98) | Moderate quality (⊕⊕⊕O)^3^ | 0.67 (0.26-1.70) | Low quality (⊕⊕OO)^1.3^ | 0.67 (0.43-1.04) | Moderate quality (⊕⊕⊕O)^3^ |
| Gabapentin, Pregabalin vs. control | 1.04 (0.80-1.36) | Moderate quality (⊕⊕⊕O)^3^ | 1.06 (0.36-3.14) | Low quality (⊕⊕OO)^1.3^ | 1.06 (0.53-2.12) | Moderate quality (⊕⊕⊕O)^3^ |
| Haloperidol vs. control | 0.68 (0.55-0.86) | High quality (⊕⊕⊕⊕) | 0.59 (0.23-1.53) | Moderate quality (⊕⊕⊕O)^1^ | 0.59 (0.37-0.95) | High quality (⊕⊕⊕⊕) |
| Ketamine vs. control | 0.94 (0.69-1.28) | High quality (⊕⊕⊕⊕) | 1.02 (0.34-3.03) | Moderate quality (⊕⊕⊕O)^1^ | 1.02 (0.51-2.05) | Moderate quality (⊕⊕⊕O)^1^ |
| Lidocaine vs. control | 1.65 (0.22-12.05) | Moderate quality (⊕⊕⊕O)^1^ | 1.55 (0.19-12.68) | Moderate quality (⊕⊕⊕O)^1^ | 1.55 (0.23-10.31) | Moderate quality (⊕⊕⊕O)^1^ |
| Melatonin vs. control | 0.90 (0.66-1.21) | Moderate quality (⊕⊕⊕O)^3^ | 0.62 (0.20-1.88) | Low quality (⊕⊕OO)^1.3^ | 0.62 (0.30-1.28) | Moderate quality (⊕⊕⊕O)^3^ |
| Atypical antipsychotics  (Olanzapine, Risperidone) vs. control | 0.36 (0.26-0.50) | High quality (⊕⊕⊕⊕) | 0.27 (0.09-0.77) | Moderate quality (⊕⊕⊕O)^1^ | 0.27 (0.14-0.51) | High quality (⊕⊕⊕⊕) |
| Propofol vs. control | None. | Moderate quality (⊕⊕⊕O)^3^ | 1.34 (0.49-3.64) | Low quality (⊕⊕OO)^1.3^ | 1.34 (0.76-2.35) | Low quality (⊕⊕OO)^1.3^ |
| Nimodipine vs. control | 0.40 (0.08-1.90) | Moderate quality (⊕⊕⊕O)^3^ | 0.36 (0.04-2.95) | Low quality (⊕⊕OO)^1.3^ | 0.36 (0.05-2.40) | Low quality (⊕⊕OO)^1.3^ |
| Ondansetron vs. control | 0.67 (0.43-1.05) | High quality (⊕⊕⊕⊕) | 0.49 (0.12-2.00) | Moderate quality (⊕⊕⊕O)^1^ | 0.49 (0.16-1.50) | Moderate quality (⊕⊕⊕O)^1^ |
| Parecoxib vs. control | 0.60 (0.33-0.96) | High quality (⊕⊕⊕⊕) | 0.53 (0.14-1.96) | Moderate quality (⊕⊕⊕O)^1^ | 0.53 (0.20-1.43) | High quality (⊕⊕⊕⊕) |
| Opioid vs. control | None | High quality (⊕⊕⊕⊕) | 1.17 (0.35-3.91) | Moderate quality (⊕⊕⊕O)^1^ | 1.17 (0.49-2.78) | Moderate quality (⊕⊕⊕O)^1^ |
| Volatile anesthetics vs. control | None | Moderate quality (⊕⊕⊕O)^3^ | 1.62 (0.35-7.43) | Low quality (⊕⊕OO)^1.3^ | 1.62 (0.46-5.69) | Low quality (⊕⊕OO)^1.3^ |

^1^ Very serious imprecision since 95% confidence interval crosses unity and with wide confidence interval suggesting probability of harm,^2^ Inconsistency, ^3^ Unclear or high risk of bias.

**Supplemental Table S3**. Adjusted SUCRA values and probability to the best and worst.

|  | SUCRA value | Probability to be the best | Probability to be the worst |
| --- | --- | --- | --- |
| Acetaminophen | 25.2 | 5.8 | 0.1 |
| Acetylcholinesterase inhibitor | 61.2 | 1.3 | 0.1 |
| Benzodiazepine | 13.1 | 0.0 | 45.0 |
| Clonidine | 23.5 | 0.0 | 7.1 |
| Dexmedetomidine | 92.1 | 38.1 | 0.0 |
| Steroid | 62.0 | 10.5 | 0.0 |
| Gabapentin, Pregabalin | 39.9 | 0.2 | 1.2 |
| Haloperidol | 67.4 | 31.3 | 0.0 |
| Ketamine | 42.2 | 0.2 | 0.8 |
| Lidocaine | 31.7 | 2.2 | 1.4 |
| Melatonin | 64.5 | 12.0 | 0.1 |
| Atypical antipsychotics  (Olanzapine, Risperidone) | 74.1 | 36.7 | 0.0 |
| Propofol | 30.1 | 0.0 | 16.1 |
| Nimodipine | 46.4 | 5.1 | 0.9 |
| Ondansetron | 7.21 | 10.1 | 0.2 |
| Parecoxib | 69.6 | 6.4 | 0.1 |
| Opioid | 36.4 | 0.1 | 2.2 |
| Volatile anesthetics | 26.6 | 0.2 | 10.9 |
| Placebo | 41.7 | 0.0 | 13.7 |

SUCRA = surface under the cumulative ranking analysis.

**Supplemental Table S4**. PRISMA Network meta-analysis Checklist of items to include when reporting a systematic review involving a network meta-analysis. The checklist was reported according to the Preferred Reporting Items for Systemic Reviews and Meta-Analyses (PRISMA) extension statements for network meta-analysis.^1^

| **Section/Topic** | **Item #** | **Checklist Item** | **Reported on Page #** |
| --- | --- | --- | --- |
| **TITLE** |  |  |  |
| Title | 1 | Identify the report as a systematic review *incorporating a network meta-analysis (or related form of meta-analysis).* | ***1*** |
|  |  |  |  |
| **ABSTRACT** |  |  |  |
| Structured summary | 2 | Provide a structured summary including, as applicable:  **Background:** main objectives  **Methods:** data sources; study eligibility criteria, participants, and interventions; study appraisal; and *synthesis methods, such as network meta-analysis.*  **Results:** number of studies and participants identified; summary estimates with corresponding confidence/credible intervals; *treatment rankings may also be discussed. Authors may choose to summarize pairwise comparisons against a chosen treatment included in their analyses for brevity.*  **Discussion/Conclusions:** limitations; conclusions and implications of findings.  **Other:** primary source of funding; systematic review registration number with registry name. | 2 |
|  |  |  |  |
| **INTRODUCTION** |  |  |  |
| Rationale | 3 | Describe the rationale for the review in the context of what is already known*, including mention of why a network meta-analysis has been conducted.* | ***3*** |
| Objectives | 4 | Provide an explicit statement of questions being addressed, with reference to participants, interventions, comparisons, outcomes, and study design (PICOS). | 3 |
|  |  |  |  |
| **METHODS** |  |  |  |
| Protocol and registration | 5 | Indicate whether a review protocol exists and if and where it can be accessed (e.g., Web address); and, if available, provide registration information, including registration number. | 12 |
| Eligibility criteria | 6 | Specify study characteristics (e.g., PICOS, length of follow-up) and report characteristics (e.g., years considered, language, publication status) used as criteria for eligibility, giving rationale. *Clearly describe eligible treatments included in the treatment network, and note whether any have been clustered or merged into the same node (with justification).* | ***12-13*** |
| Information sources | 7 | Describe all information sources (e.g., databases with dates of coverage, contact with study authors to identify additional studies) in the search and date last searched. | 13 |
| Search | 8 | Present full electronic search strategy for at least one database, including any limits used, such that it could be repeated. | Text S3 |
| Study selection | 9 | State the process for selecting studies (i.e., screening, eligibility, included in systematic review, and, if applicable, included in the meta-analysis). | 13 |
| Data collection process | 10 | Describe method of data extraction from reports (e.g., piloted forms, independently, in duplicate) and any processes for obtaining and confirming data from investigators. | 13,14 |
| Data items | 11 | List and define all variables for which data were sought (e.g., PICOS, funding sources) and any assumptions and simplifications made. | 5 |
| **Geometry of the network** | **S1** | Describe methods used to explore the geometry of the treatment network under study and potential biases related to it. This should include how the evidence base has been graphically summarized for presentation, and what characteristics were compiled and used to describe the evidence base to readers. | ***15, Figure 2*** |
| Risk of bias within individual studies | 12 | Describe methods used for assessing risk of bias of individual studies (including specification of whether this was done at the study or outcome level), and how this information is to be used in any data synthesis. | 14-15, Figure S6 |
| Summary measures | 13 | State the principal summary measures (e.g., risk ratio, difference in means). *Also describe the use of additional summary measures assessed, such as treatment rankings and surface under the cumulative ranking curve (SUCRA) values, as well as modified approaches used to present summary findings from meta-analyses.* | 15,16 |
| Planned methods of analysis | 14 | Describe the methods of handling data and combining results of studies for each network meta-analysis. This should include, but not be limited to:   - *Handling of multi-arm trials;* - *Selection of variance structure;* - *Selection of prior distributions in Bayesian analyses; and* - *Assessment of model fit.* | 15,16 |
| **Assessment of Inconsistency** | **S2** | Describe the statistical methods used to evaluate the agreement of direct and indirect evidence in the treatment network(s) studied. Describe efforts taken to address its presence when found. | 15,16 |
| Risk of bias across studies | 15 | Specify any assessment of risk of bias that may affect the cumulative evidence (e.g., publication bias, selective reporting within studies). | **14,15** |
| Additional analyses | 16 | Describe methods of additional analyses if done, indicating which were pre-specified. This may include, but not be limited to, the following:   - Sensitivity or subgroup analyses; - Meta-regression analyses; - *Alternative formulations of the treatment network; and* - *Use of alternative prior distributions for Bayesian analyses (if applicable).* | ***16,17*** |
|  |  |  |  |
| **RESULTS†** |  |  |  |
| Study selection | 17 | Give numbers of studies screened, assessed for eligibility, and included in the review, with reasons for exclusions at each stage, ideally with a flow diagram. | 4, Figure 1 |
| **Presentation of network structure** | **S3** | Provide a network graph of the included studies to enable visualization of the geometry of the treatment network. | ***Figure 2*** |
| **Summary of network geometry** | **S4** | Provide a brief overview of characteristics of the treatment network. This may include commentary on the abundance of trials and randomized patients for the different interventions and pairwise comparisons in the network, gaps of evidence in the treatment network, and potential biases reflected by the network structure. | ***4,5, Table 1*** |
| Study characteristics | 18 | For each study, present characteristics for which data were extracted (e.g., study size, PICOS, follow-up period) and provide the citations. | Table S1 |
| Risk of bias within studies | 19 | Present data on risk of bias of each study and, if available, any outcome level assessment. | Figure S6 |
| Results of individual studies | 20 | For all outcomes considered (benefits or harms), present, for each study: 1) simple summary data for each intervention group, and 2) effect estimates and confidence intervals. *Modified approaches may be needed to deal with information from larger networks.* | ***Figure 3, Table S1*** |
| Synthesis of results | 21 | Present results of each meta-analysis done, including confidence/credible intervals. *In larger networks, authors may focus on comparisons versus a particular comparator (e.g. placebo or standard care), with full findings presented in an appendix. League tables and forest plots may be considered to summarize pairwise comparisons.* If additional summary measures were explored (such as treatment rankings), these should also be presented. | ***4,5,6,***  ***Figure 3,***  ***Table 2*** |
| **Exploration for inconsistency** | **S5** | Describe results from investigations of inconsistency. This may include such information as measures of model fit to compare consistency and inconsistency models, *P* values from statistical tests, or summary of inconsistency estimates from different parts of the treatment network. | ***5,6,***  ***Figure S1, Table S5*** |
| Risk of bias across studies | 22 | Present results of any assessment of risk of bias across studies for the evidence base being studied. | 5,6,  Table S2 |
| Results of additional analyses | 23 | Give results of additional analyses, if done (e.g., sensitivity or subgroup analyses, meta-regression analyses*, alternative network geometries studied, alternative choice of prior distributions for Bayesian analyses,* and so forth). | **5,6,7,**  **Figure S2, Figure S7-S18.** |
|  |  |  |  |
| **DISCUSSION** |  |  |  |
| Summary of evidence | 24 | Summarize the main findings, including the strength of evidence for each main outcome; consider their relevance to key groups (e.g., healthcare providers, users, and policy-makers). | 8 |
| Limitations | 25 | Discuss limitations at study and outcome level (e.g., risk of bias), and at review level (e.g., incomplete retrieval of identified research, reporting bias). *Comment on the validity of the assumptions, such as transitivity and consistency. Comment on any concerns regarding network geometry (e.g., avoidance of certain comparisons).* | 10,11 |
| Conclusions | 26 | Provide a general interpretation of the results in the context of other evidence, and implications for future research. | 11 |
|  |  |  |  |
| **FUNDING** |  |  |  |
| Funding | 27 | Describe sources of funding for the systematic review and other support (e.g., supply of data); role of funders for the systematic review. This should also include information regarding whether funding has been received from manufacturers of treatments in the network and/or whether some of the authors are content experts with professional conflicts of interest that could affect use of treatments in the network. | ***1, 18*** |

PICOS = population, intervention, comparators, outcomes, study design.

* Text in italics indicates wording specific to reporting of network meta-analyses that has been added to guidance from the PRISMA statement.

† Authors may wish to plan for use of appendices to present all relevant information in full detail for items in this section.

^1^Hutton, B. et al. The PRISMA extension statement for reporting of systematic reviews incorporating network meta-analyses of health care interventions: checklist and explanations. Annals of internal medicine 162, 777-784 (2015).

**Supplemental Table S5**. Evaluation of the model fit.

| Model assumption | Dbar | pD | DIC | Data points |
| --- | --- | --- | --- | --- |
| Incidence of delirium |  |  |  |  |
| Fixed effect model | 251.40 | 99.89 | 351.29 | 176.00 |
| Random effect model | 175.81 | 129.12 | 304.93 | 176.00 |

The variables were calculated using gemtc package for R.

**Posterior mean of deviance (Dbar)** measures the model fit, and is used to check formally whether a model’s fit is satisfactory. This is the posterior mean of the deviance under the current model minus the deviance for the saturated mode. We can then compare the value of Dbar to the number of independent data points to check if the model fit can be improved.

**Leverage (pD)** measures the complexity of a model that reasonably describes the data. The pD also is termed the effective number of parameters, and is calculated as the posterior mean of the residual deviance minus the deviance at the posterior mean of the fitted values.

**Deviance Information Criterion (DIC)** is particularly useful for comparing different parameter models for the same likelihood and data, for example fixed and random effects models or fixed effect models with and without covariates. The model with smaller DIC is estimated to be the model that best and most parsimonious predicts the data observed. As shown in above table, the random effect model was preferred than the fixed effect model.
